# Genetic and Biochemical Characterization of Halogenation and Drug Transportation Genes Encoded in the Albofungin Biosynthetic Gene Cluster

**DOI:** 10.1128/aem.00806-22

**Published:** 2022-08-24

**Authors:** Zhe-Chong Wang, I-Wen Lo, Kuan-Hung Lin, An Ning Cheng, Saeid Malek Zadeh, Yen-Hua Huang, Tsung-Lin Li

**Affiliations:** a Genomics Research Center, Academia Sinica, Taipei, Taiwan; b Institute of Biomedical Informatics, National Yang Ming Chiao Tung University, Taipei, Taiwan; c Biotechnology Center, National Chung Hsing University, Taichung City, Taiwan; University of Manchester

**Keywords:** albofungin, antibiotic detoxification, antibiotic resistance, biosynthetic gene cluster, halogenase, transporter

## Abstract

Albofungin, a hexacyclic aromatic natural product, exhibits broad-spectrum antimicrobial activity. Its biosynthesis, regulation, and resistance remain elusive. Here, we report the albofungin (*abf*) biosynthetic gene cluster (BGC) from its producing strain Streptomyces tumemacerans JCM5050. The nascent *abf* BGC encodes 70 putative genes, including regulators, transporters, type II polyketide synthases (PKSs), oxidoreductase, and tailoring enzymes. To validate the intactness and functionality of the BGC, we developed an Escherichia coli-*Streptomyces* shuttle bacterial artificial chromosome system, whereby the *abf* BGC was integrated into the genome of a nonproducing host via heterologous conjugation, wherefrom albofungin can be produced, confirming that the BGC is in effect. We then delimited the boundaries of the BGC by means of *in vitro* CRISPR-Cas9 DNA editing, concluding a minimal but essential 60-kb *abf* BGC ranging from *orfL* to *abf58*. The *orfA* gene encoding a reduced flavin adenine dinucleotide (FADH_2_)-dependent halogenase was examined and is capable of transforming albofungin to halogen-substituted congeners *in vivo* and *in vitro*. The *orfL* gene encoding a transporter was examined *in vivo*. The presence/absence of *orfA* or *orfL* demonstrated that the MIC of albofungin is subject to alteration when an extracellular polysaccharide intercellular adhesin was formed. Despite that halogenation of albofungin somewhat increases binding affinity to transglycosylase (TGase), albofungin with/without a halogen substituent manifests similar *in vitro* antimicrobial activity. Halogenation, however, limits overall dissemination and effectiveness given a high secretion rate, weak membrane permeability, and high hydrophobicity of the resulting products, whereby the functions of *orfA* and *orfL* are correlated with drug detoxification/resistance for the first time.

**IMPORTANCE** Albofungin, a natural product produced from Streptomycetes, exhibits bioactivities against bacteria, fungi, and tumor cells. The biosynthetic logic, regulations, and resistance of albofungin remain yet to be addressed. Herein, the minimal albofungin (*abf*) biosynthetic gene cluster (BGC) from the producing strain Streptomyces tumemacerans JCM5050 was precisely delimited using the Escherichia coli*-Streptomyces* shuttle bacterial artificial chromosome system, of which the gene essentiality was established *in vivo* and *in vitro*. Next, we characterized two genes *orfA* and *orfL* encoded in the *abf* BGC, which act as a reduced flavin adenine dinucleotide (FADH_2_)-dependent halogenase and an albofungin-congeners transporter, respectively. While each testing microorganism exhibited different sensitivities to albofungins, the MIC values of albofungins against testing strains with/without *orfA* and/or *orfL* were subject to considerable changes. Halogen-substituted albofungins mediated by OrfA manifested overall compromised dissemination and effectiveness, revealing for the first time that two functionally distinct proteins OrfA and OrfL are associated together, exerting a novel “belt and braces” mechanism in antimicrobial detoxification/resistance.

## INTRODUCTION

**FIG 1:**
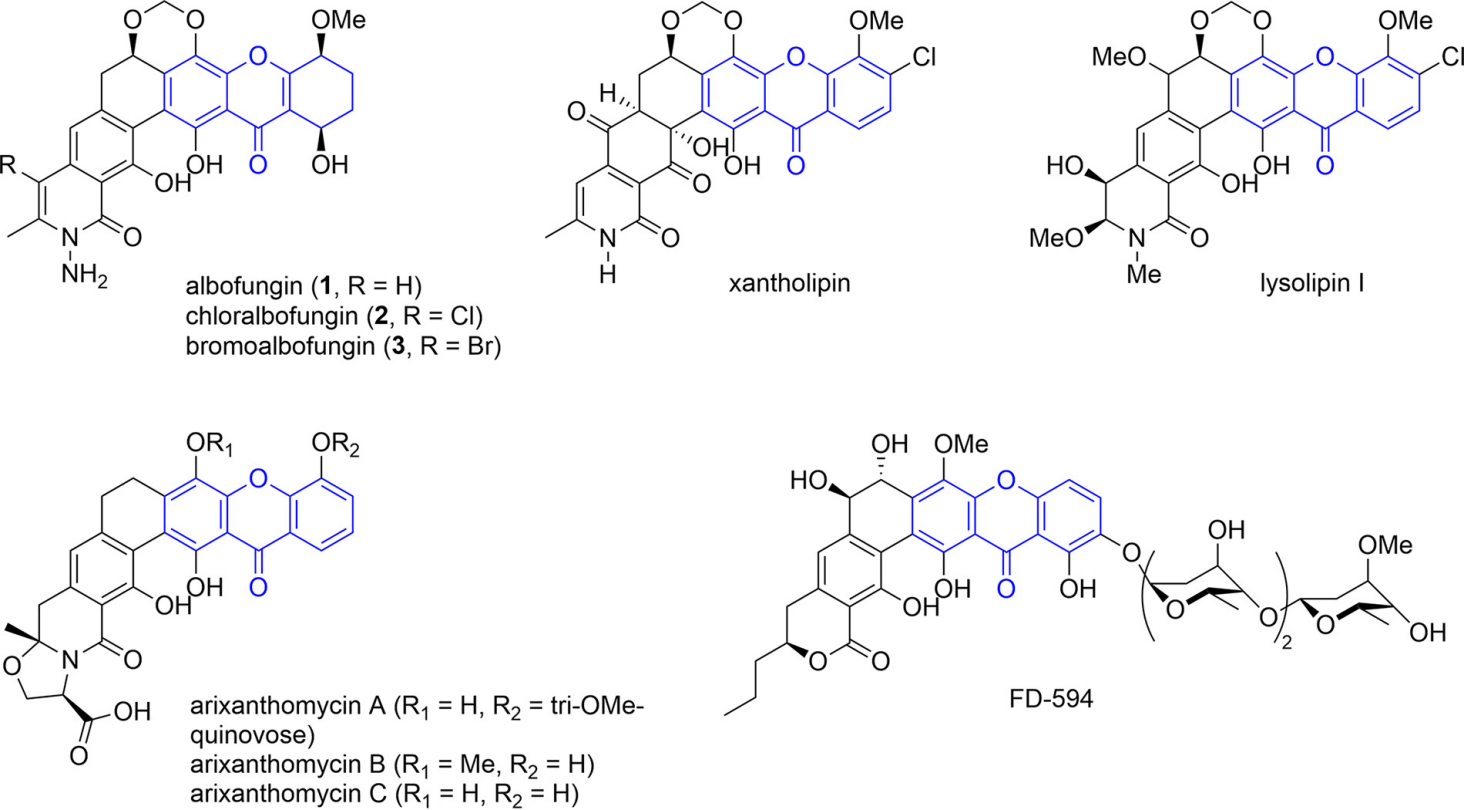
Chemical structures of albofungin congeners (compounds 1 to 3) and selected xanthone-containing analogs (xantholipin, lysolipin I, arixanthomycins, and FD-594). The xanthone moiety is colored blue.

Multidrug-resistant microbes evolve and spread at an unimaginable speed; therefore, discovering/developing new and more effective antibiotics becomes an urgent task. The World Health Organization (WHO) recently issued a list of alarming drug-resistant pathogens, and antibiotic resistance is one of the biggest global public health threats (1). The natural product albofungin (compound 1) is the focal point of the current study, as it displays promising antibacterial activities against a broad spectrum of pathogenic bacteria with MIC values in submicromolar to nanomolar ranges. Albofungin binds to the transglycosylase (TGase) domain of penicillin-binding proteins (PBPs) required in cell wall biosynthesis (2). A new albofungin analog turbinmicin, identified from marine *Micromonospora* sp., manifested potent *in vitro* and mouse model efficacy against the pandrug-resistant fungus Candida auris (3). Over the years, a number of albofungin analogs (for example, xantholipin from *Streptomyces* sp. [4], lysolipin from Streptomyces violaceoniger Tü96 [5], arixanthomycins A to C from a heteroexpressed *S. albus* containing a selected biosynthetic gene cluster [BGC] from the environmental DNA [eDNA] library [6], and FD-594 from *Streptomyces* sp. TA-0256 [7] [Fig. 1]) with desired activities to a wide extent have been reported and touted from time to time.

Albofungin (compound 1) is an angular aromatic polyketide natural product featuring a hexacyclic ring system and an unusual hydrazine moiety that is not found in other polycyclic xanthone congeners. It was first isolated in 1959 and confirmed as a metabolite of Actinomyces tumemacerans (8). Previous investigations suggested that the polycyclic xanthone backbone is derived from a single polyketide (PK) chain catalyzed by type II polyketide synthases (PKSs) at the expense of 12 or 13 molecules of malonyl-CoA. The polyketide chain subsequently undergoes cyclization, reduction, and dehydration in sequence, resulting in the unique angular aromatic framework. Tailoring enzymes, such as oxygenase, reductase, methyltransferase, halogenase, and glycosyltransferase, come next in sequence to transform the polyketide framework into mature polycyclic xanthone-polyketides (9, 10). A 72-kb albofungin BGC (*alb* BGC) from Streptomyces chrestomyceticus BCC 24770 was recently reported (11), but the boundaries of *alb* BGC were not defined, and the gene product functions were not validated.

In this study, we first sequenced the genome of *S. tumemacerans* JCM5050 by next-generation sequencing (NGS) and identified the given BGC using antibiotics & Secondary Metabolite Analysis Shell (antiSMASH), a popular tool for mining microbial secondary metabolite BGCs (12). Our initial analysis identified a putative BGC (72,519 nucleotides [nt]) containing a characteristic type II polyketide synthase that may mediate the formation of the polycyclic xanthone backbone. Despite that both BGCs (*abf* from *S. tumemacerans* JCM5050 and *alb* from *S. chrestomyceticus* BCC 24770) share 97% identity, the true boundaries of the BGCs were not delimited and validated. As such, we systematically constructed a host of heterologous expression strains from Streptomyces albus J1074::ermE*-*crp*_SC_ with *abf*-containing bacterial artificial chromosomes (BACs) of various lengths edited using CRISPR-Cas9 technology (13). A minimal 60-kb *abf* BGC (GenBank accession number ON399210) was thus determined, ranging from *orfL* to *abf58*, in which two genes *orfA* and *orfL* were characterized in relation to self-protection/resistance other than genes encoding tetracycline resistance repressors, TetR-like proteins.

Gene analysis revealed two genes, *orfA* and *orfL*, that respectively encode a flavin-dependent halogenase and a proton-dependent oligopeptide transporter (POT) (14). Flavin-dependent halogenases that take effect need to reduce their flavin prosthetic group to activate molecular oxygen (15). A halide ion is presumed to attack the distal oxygen atom of a 4α-flavin hydroperoxide resultant so that a hypohalite intermediate is formed and transferred to an active-site lysine residue. This halogen-amine adduct is capable of halogenating an appropriate nearby substrate by an electrophilic substitution reaction (16–18). However, OrfL is a proton-dependent amino acid/oligopeptide transporter in the major facilitator superfamily (MFS; POT), a transmembrane protein in reference to chemiosmotic permeability for transportation of oligopeptides or antibiotics (19, 20). Two multidrug-resistance families, MFS and the ATP-binding cassette (ABC) superfamily, are frequently recruited in all classes of organisms for recognizing and exporting a given type of substrates through cell membranes concomitantly in exchange with protons. Several lines of evidence have underscored that MFS-deficient mutants increase antibiotic sensitivity (21).

We performed *in vivo* and *in vitro* assays concluding that OrfA is a halogenase halogenating albofungin to increase binding affinity with TGase *in vitro*, while the antimicrobial activity against the testing bacteria is offset to some extent because of weak membrane permeability resulting from reduced hydrophilicity. In parallel, OrfL was determined to be a transporter capable of pumping out albofungin and congeners thereof, highlighting that *orfL* is an intrinsic drug detoxification/resistance gene in the producing strains and/or the gene-acquired microorganisms. Given that *orfA* and *orfL* are two genes that likely contribute to the modification and secretion of albofungin, we were thus prompted to illustrate their mutual connection.

## RESULTS

### Identification and delimitation of *abf* BGC.

**FIG 2:**
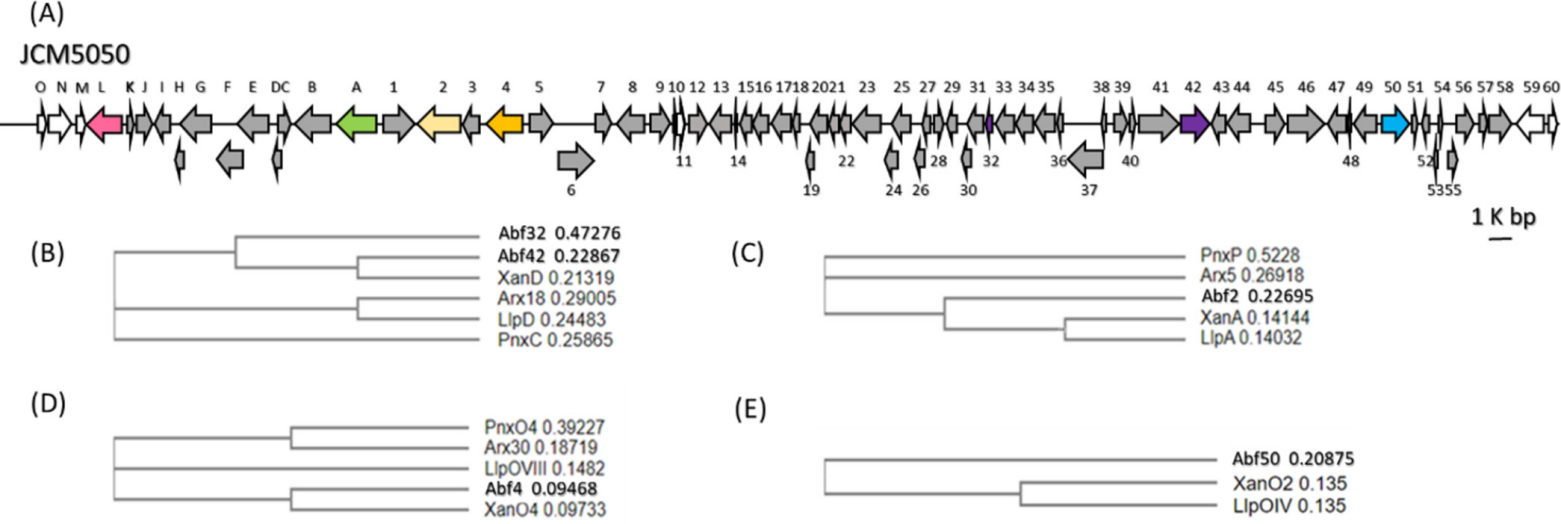
Gene organization of the albofungin biosynthetic gene cluster (*abf* BGC) in *S. tumemacerans* JCM5050. (A) Map of the *abf* BGC. (B to E) Phylogenetic analyses for the min-PKS and selected tailoring enzymes between the *abf* BGC and other selected BGCs. Abf32 and Abf42 are two distinguishable ACP proteins (B). Abf2 (C), Abf4 (D), and Abf50 (E) are three essential modifier enzymes required for albofungin (compound 1) maturation, with high sequence similarity to homologous enzymes encoded in other BGCs. The multisequence was aligned using ClustalW version 2.0, and the phylogenetic tree was generated by the neighbor-joining tree method without distance corrections.

Albofungin (compound 1) exhibits a core framework akin to lysolipin and xantholipin; all of them share a xanthone-containing hexacyclic aromatic backbone (4, 5). Because of promising biomedical implications, many BGCs of angular aromatic polyketide natural products have been made available, including xantholipin (*xan*; 48% gene similarity), lysolipin I (*lip*; 54% gene similarity), arixanthomycins A to C (*arx*; 57% gene similarity), and FD-594 (*pnx*; 34% gene similarity). For identifying the albofungin BGC in *S. tumemacerans* JCM5050, its genomic DNA (gDNA) was isolated and sequenced by NGS. Assembly of the sequencing data yielded 30 contigs, covering 10,250,488 bp in total (data not shown). A 72,519-nt DNA fragment containing 81 open reading frames (ORFs; Fig. 2 and Table S3 in the supplemental material) that includes the characteristic type II PKS (T2PKS)-type of secondary metabolite cluster was identified by antiSMASH and named *abf*. In terms of type II polyketide biosynthesis, the initial step is generally executed by a minimal polyketide synthase (min-PKS). A min-PKS is composed of two ketosynthases, KS_α_ and KS_β_, alongside one acyl carrier protein (ACP) for the iterative condensation of malonyl-CoA to an unreduced polyketide chain ranging from 16 to 30 carbons in length (6). The min-PKSs encoded in *abf* are *abf23*, *abf24*, *abf25*, and *abf32*, encoding a ketosynthase β/chain length factor, a ketosynthase α, an ACP synthase, and an acyl carrier protein, and are known as a whole to assemble a polyketide precursor with high sequence similarity to typical T2PKSs, whereby a C_26_ polyketide chain is formed at the expense of 13 molecules of malonyl-CoA. Several tailoring enzymes encoded in the *abf* BGC for the post-PKS modifications were further identified with counterparts in the *xan* BGC. For example, *abf2* (1,866 bp) encodes an asparagine synthetase sharing 54% sequence identity to *xanA* for the formation of the δ-lactam ring; *abf4* (1,584 bp) encodes a flavin adenine dinucleotide (FAD)-dependent monooxygenase with 81% sequence identity to *xanO4*, which executes a Baeyer-Villiger oxidation reaction for formation of the xanthone moiety (22); and *abf50* (1,200 bp) encodes a cytochrome P450 enzyme with 59% sequence identity to *xanO2* for the formation of the 17,19-methylenedioxy moiety (4). These specialized enzymes are illustrated in Fig. 2, where the evolutionary relationship is established.

During preparation of the manuscript, a 72-kb albofungin BGC (*alb* BGC) in the strain *S. chrestomyceticus* BCC 24770 was independently reported by She et al. (11), of which neither the boundaries nor the functions of biosynthetic genes encoded were determined and verified. Although sequence similarity is as high as 97%, several dissimilarities indeed exist. For instance, *abf11* (243 bp) encoding a transposase family protein, *abf12* (783 bp) encoding a methyltransferase, and *abf13* (942 bp) encoding a sugar kinase are not found in *alb* (Fig. 2), suggesting that the fermentation of *S. chrestomyceticus* BCC 24770 will not give rise to *N*-methyl or glycosyl albofungin.

**FIG 3:**
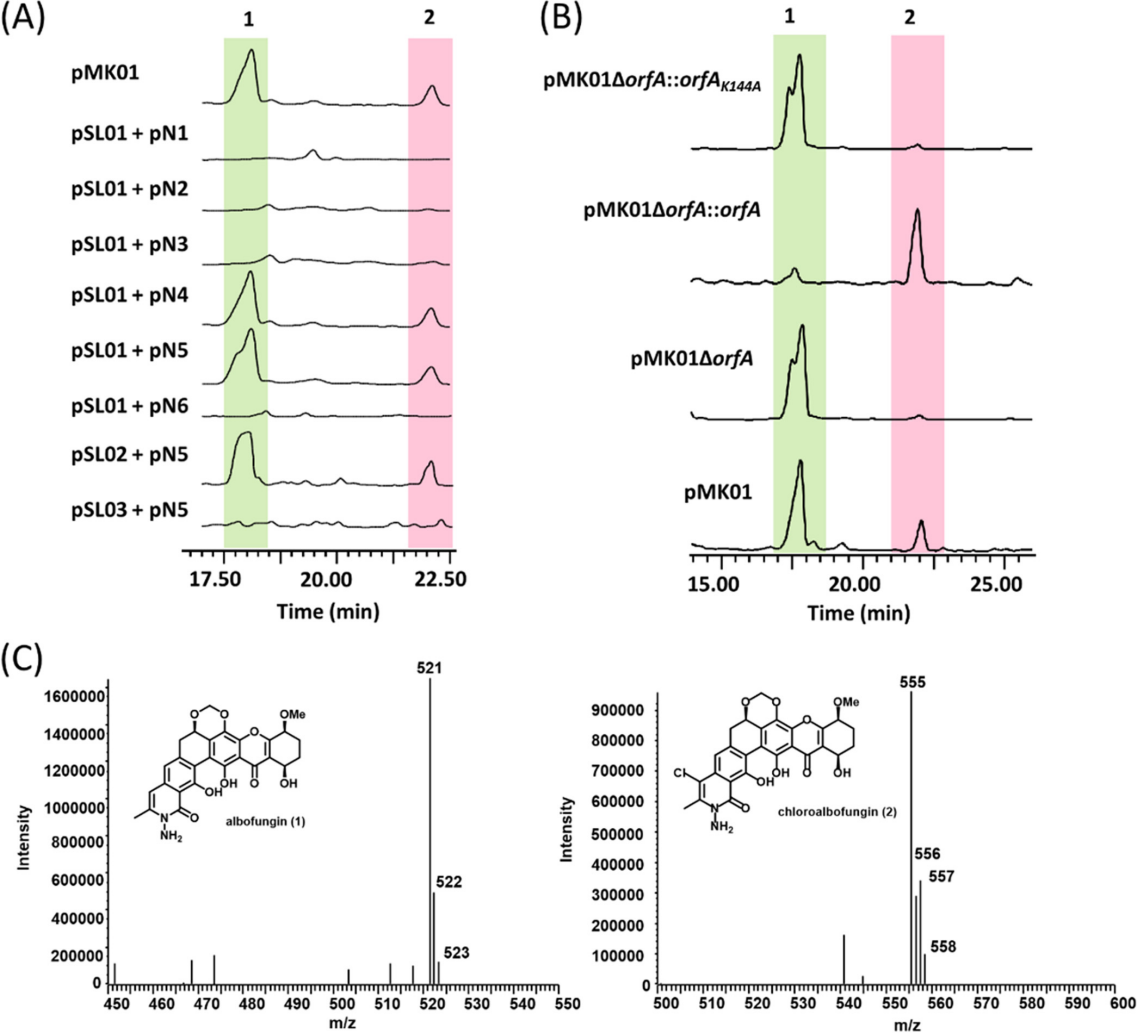
LC-MS analyses for albofungin (compound 1) and chloroalbofungin (compound 2). (A) LC-MS chromatographs (UV_280_ absorption) of compounds 1 and 2 from the cultures of pMK01 and six BAC constructs coexpressed with pSL01+pN1 (*abf2* to *abf61*), pSL01+pN2 (*orfB* to *abf61*), pSL01+pN3 (*orfI* to *abf61*), pSL01+pN4 (*orfO* to *abf61*), pSL01+pN5 (*orfL* to *abf61*), pSL01+pN6 (*orfK* to *abf61*), pSL02+pN5 (*orfL* to *abf58*), or pSL03+pN5 (*orfL* to *abf56*). (B) LC-MS chromatographs of compounds 1 and 2 for four BAC strains, pMK01, pMK01Δ*orfA*, pMK01Δ*orfA*::*orfA*, and pMK01Δ*orfA*::*orfA*_K144A_. (C) MS spectra of compounds 1 and 2.

To validate that the *abf* BGC identified from the *S. tumemacerans* JCM5050 strain is functional and intact, a BAC system was designed and constructed by creating an Escherichia coli-*Streptomyces* shuttle vector to carry the newly identified *abf* BGC from *S. tumemacerans* JCM5050 through allelic exchange. In brief, the original selection marker in an *abf*-containing BAC clone was replaced with a different selection marker for further CRISPR-Cas9 editing (see Materials and Methods and Fig. S2). After intergeneric conjugation and transformation of pmk01 into a host strain *S. albus* J1074::ermE*-*crp*_SC_, the bacterial transconjugant *S. albus* J1074::ermE*-*crp*_SC__pmk01 (pMK01) was cultured for production of albofungin (compound 1). By liquid chromatography-mass spectrometry (LC-MS) analysis (Fig. 3C), both albofungin (compound 1) and chloroalbofungin (compound 2) can be detected from the cultures of pMK01, indicating that the *abf* BGC is valid and intact.

To delimit the boundaries of the *abf* BGC, two series of BAC clones (pSL01 to pSL03 and pN1 to pN6) carrying various lengths of *abf* BGC were individually constructed and expressed in the host strain *S. albus* J1074::erm ermE*-*crp*_SC_. In brief, pSL01, pSL02, and pSL03 carry *abf5* to *abf61*, *abf5* to *abf58*, and *abf5* to *abf56* fragments of *abf* BGC, respectively. In contrast, clones pN1 to pN6 carry *abf2* to *abf4*, *orfB* to *abf4*, *orfI* to *abf4*, *orfO* to *abf4*, *orfL* to *abf4*, and *orfK* to *abf4* fragments of *abf* BGC, respectively. LC-MS analysis of these clones (Fig. 3A) revealed that only compounds 1 and 2 were produced from clones coexpressing pSL01/pSL02 and pN4/pN5, suggesting the *abf* BGC is delimited between *orfL* and *abf58*. In terms of directionality, the 60-kb minimal *abf* BGC starts from *orfL* (encoding an MFS transporter, 1,506 bp) and ends at *abf58* (encoding an FAD-binding monooxygenase, 1,053 bp).

### The role of OrfA in the albofungin biosynthetic pathway.

Because both albofungin (compound 1) and chloroalbofungin (compound 2) were isolated together with a ratio of 10:1 from the culture of pMK01, the *orfA* gene (1,752 bp) in the *abf* BGC encoding a tryptophan (Trp)-7-like halogenase was proposed to commit the chlorination of compound 1 at C-24 to compound 2. Sequence alignment amid OrfA, XanH (the halogenase encoded in *xan* BGC), and LlpH (the halogenase encoded in *lip* BGC), however, showed that OrfA has low sequence similarity to XanH (35%) and LlpH (34%), while both XanH and LipH share high sequence similarity (70%). Nevertheless, all of them belong to the FAD-dependent halogenase (FDH) family in contrast to the nonheme iron-dependent halogenases (23).

**FIG 4:**
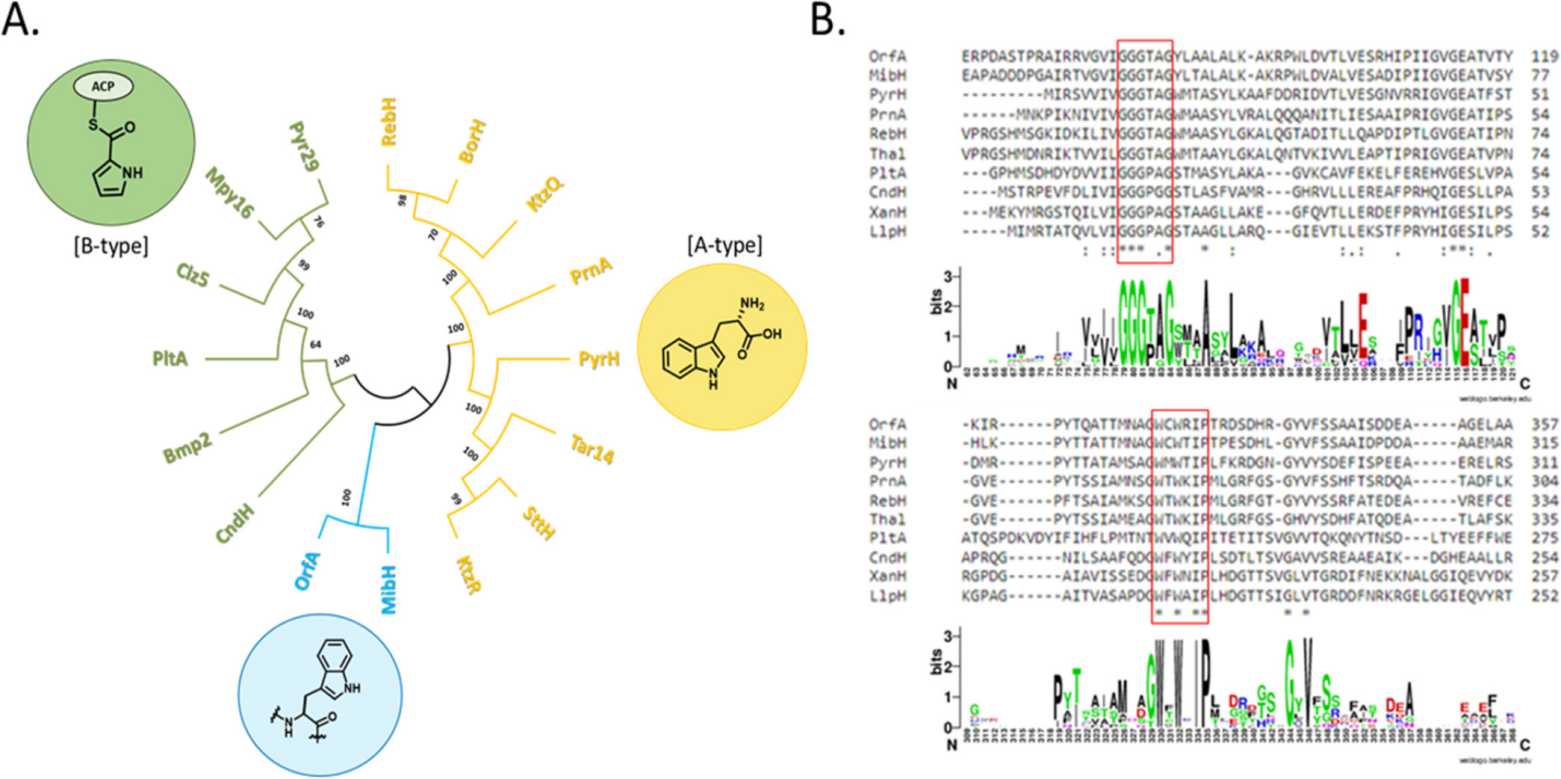
*In silico* analysis of the FAD-dependent halogenase (FDH) OrfA. (A) The ML tree for FDHs in different clades. Each clade is given by bootstrap percentage values, and each terminal branch corresponds to a given enzyme in FDHs. Color stands for a specific substrate for one type of FDH; blue, peptides; yellow, free Trp; green, phenyl, alkenyl, and alkyl groups of substrates tethered to a carrier protein (tethered substrates). (B) Multisequence alignment of OrfA with selected FDHs, MibH, PyrH, PrnA, RebH, ThaI, PltA, CndH, XanH, and LlpH. The red frames highlight two conserved motifs, GXGXXG and WXWXIP, in FDHs, which are also presented in sequence logos. The numbering for two logos does not reflect the correct individual sequence number. The multisequence alignment was generated by ClustalW version 2.0, and the sequence logos were generated by WebLogo version 2.8.2.

In *in silico* analysis, OrfA is categorized into a clade of the FDH family (Fig. 4A) according to the maximum likelihood phylogenetic unrooted (ML) tree (24). All members of the FDH family share a conserved Rossmann domain with a GXGXXG motif for flavin binding and a WXWXIP motif for preventing a monooxygenase-like reaction from occurring by keeping substrates a distance from flavin (Fig. 4B) (25, 26). Based on the multisequence alignment, the highly conserved Lys/Glu pair in the active site was identified as catalytically relevant to the halogenation reaction for FDHs, namely, Lys-144 and Glu-397 in OrfA (Fig. S4).

To verify that OrfA is the committed FDH transforming compound 1 to compound 2 *in vivo*, we constructed three pMK01-derived strains: an *orfA*-deficient strain, pMK01Δ*orfA*, and two *orfA*-complement strains, pMK01Δ*orfA*::*orfA* and pMK01Δ*orfA*::*orfA*_K144A_. LC-MS analysis revealed that compound 1 is present in all of the crude extracts from the fermentation medium of pMK01, pMK01Δ*orfA*, pMK01Δ*orfA*::*orfA*, and pMK01Δ*orfA*::*orfA*_K144A_. As expected, compound 2 was not detected in the fermentation extracts from the OrfA-deleted and OrfA-inactivated strains, pMK01Δ*orfA* and pMK01Δ*orfA*::*orfA*_K144A_, by LC-MS. In contrast, the production of compound 2 was fully restored in the *orfA*-complement strain pMK01Δ*orfA*::*orfA*; compound 2 remained absent in the *orfA*-complement strain pMK01Δ*orfA*::*orfA*_K144A_, indicating that Lys-144 of OrfA is an essential residue in the OrfA-mediated halogenation reactions (Fig. 3B). All of these *in vivo* consequences converge to the fact that OrfA is an intrinsic halogenase responsible for the transformation of compound 1 to compound 2 in the late stage of albofungin biosynthesis.

### Enzymatic activity and kinetics of OrfA.

**FIG 5:**
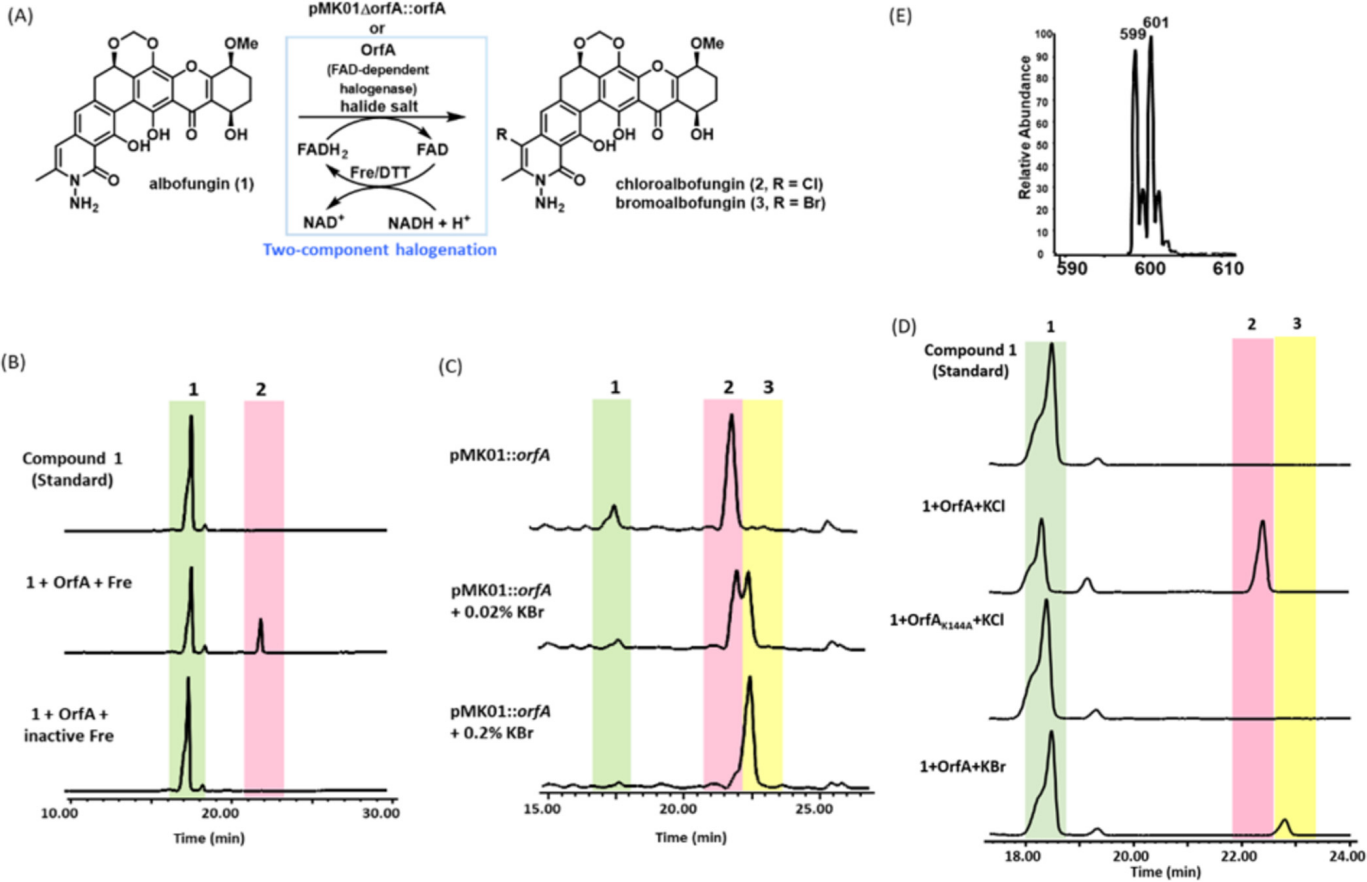
Halogenation reactions catalyzed by OrfA. (A) The OrfA-mediated halogenation reactions were conducted with the supplementation of KF, KCl, KBr, or KI, whereby only reactions supplemented with KCl or KBr can produce the corresponding product. (B) LC-MS traces for the *in vitro* assays conducted in the presence of OrfA alongside Fre or inactive Fre. Fre was inactivated by heating for 10 min at 95°C. (C) LC-MS traces for the production of bromoalbofungin (compound 3) in *S. albus* J1074::ermE*-*crp*_SC__pmk01Δ*orfA*::*orfA*, the *orfA*-complement strain, with 0.02% or 0.2% KBr. (D) The LC traces of the *in vitro* reaction of OrfA with FADH_2_, O_2_, and KCl or KBr. (E) MS spectrum of compound 3.

Although FDHs exhibit some substrate promiscuity, often introducing various halide anions into substrates contingent on their relative level in the environment, FDHs normally prefer a given halide anion (25, 27–29). To assess the preference, one could use an enzymatic or fermentation assay supplemented with selected or mixed halogens in reaction solutions or fermentation medium (30–32). In this study, we performed a halide salt-feeding assay with the *orfA*-complement strain pMK01Δ*orfA*::*orfA* and the producing strain *S. tumemacerans* JCM5050 alongside cell-free *in vitro* enzymatic halogenation reactions to explore the substrate scope of OrfA (Fig. 5A). Six selected halide salts, potassium fluoride (KF), potassium chloride/sodium chloride (KCl/NaCl), potassium bromide/sodium bromide (KBr/NaBr), and potassium iodide (KI), were individually added to the fermentation medium or reaction buffer solutions in the presence of *orfA*/OrfA, of which only the media/reactions fed with KCl/KBr can be expected to yield products to some extent, and iodo-substituted albofungin can barely be detected for the media/reactions fed with KI (Fig. 5C and D and Fig. S5 to S7). Given that OrfA and MibH (14, 15) belong to the same clade in the ML tree analysis (Fig. 4A), the flavin cofactor is required to reduce FADH_2_ in OrfA to activate molecular oxygen in the first place. For this reason, we cloned and purified the flavin reductase (Fre) from E. coli (33) to render FADH_2_ *in situ* in *in vitro* enzyme-coupled reactions. Given the coupled reactions with OrfA and Fre, halogenated products emerged corresponding to the presence of KCl in the solution (Fig. 5B), confirming that OrfA is a two-component halogenase requiring an auxiliary partner reductase Fre. The *in vitro* enzymatic reactions of OrfA with FADH_2_, O_2_, and KCl or KBr also generated the same halogenated products as the coupled reactions (Fig. 5D). The yield of chlorinated product 2 apparently outweighed that of brominated product 3 on the same basis (Fig. 5D and E), suggesting that the Cl ion is relatively more favorable than the Br ion in the OrfA-mediated halogenation reactions.

The basic kinetic parameters of OrfA were determined under the pseudofirst-order condition with saturated KCl and 1,4-dithiothreitol (DTT) in place of Fre (Fig. S8), whereby the turnover rate (*k*_cat_, min^−1^), Michaelis constant (*K*_M_, μM), and enzyme specificity (*k*_cat_/*K*_m_, μM^−1^min^−1^) were estimated to be 1.44, 42, and 0.034, respectively, which are comparable to those reported previously (Trp halogenases act on different positions of the indole ring) (Table 1).

**TABLE 1:** Kinetic comparison of OrfA and other FDHs

Enzymes (position, reference)	*k*_cat_ (min^−1^)	*K*_M_ (μM)	*k*_cat_/*K*_M_ (μM^−1^min^−1^)
OrfA (C-7, this work)	1.44 ± 0.25	42.02 ± 0.93	0.034 ± 0.003
RebH (C-7, 76)	0.6 ± 0.1	28.7 ± 1.3	0.02 ± 0.004
PrnA (C-7, 76)	1.1 ± 0.1	20.7 ± 0.1	0.05 ± 0.005
KtzR (C-7, 76)	0.4 ± 0.1	34.1 ± 2.1	0.01 ± 0.003
SttH (C-6, 76)	1.7 ± 0.1	25.3 ± 3.2	0.07 ± 0.01
Tar14 (C-6, 76)	0.42 ± 0.05	12.0 ± 1.9	0.04 ± 0.01
PyrH (C-5, 76)	2.4 ± 0.4	15.2 ± 4.2	0.16 ± 0.05

### Structural elucidation of compounds 1 to 3.

Compounds 1 and 2 can be directly isolated from the cultures of pMK01 under typical conditions, while compound 3 can only be isolated from the same condition with the extra addition of KBr. Compounds 1 to 3 were determined by nuclear magnetic resonance (NMR) and MS to be albofungin, chloroalbofungin, and bromoalbofungin, respectively (Fig. 5A and Tables S4 and S5), in agreement with those reported previously (2, 11, 34). Compound 3 has a molecular formula of C_27_H_23_BrN_2_O_9_ (17 degrees of unsaturation [DOU] and the characteristic isotopic profile) given the pseudomolecular ion *m*/*z* 599.40 [M+H]^+^ from triple quadrupole mass spectrometer (TQ-MS). Compared with chloroalbofungin (compound 2), the proton signal at H-24 is likewise absent, and the chemical shifts/patterns are virtually identical to those of compounds 2 and 3 except for some minor alterations near C-24; namely, H-22 (δ_H_ 7.33, s/7.32, s), H_3_-26 (δ_H_ 2.66, s/2.74, s), C-1 (δ_C_ 162.7/162.2), C-2 (δ_C_ 108.7/108.8), C-3 (δ_C_ 157.1/158.0), C-4 (δ_C_ 114.6/114.4), C-21 (δ_C_ 141.7/141.6), C-22 (δ_C_ 111.5/114.2), C-23 (δ_C_ 133.6/134.6), C-24 (δ_C_ 109.9/100.2), C-25 (δ_C_ 139.8/141.2), and C-26 (δ_C_ 16.3/20.3), because the aromatic hydrazine ring system (A and B rings) is somewhat influenced by a given halogen substituent. In conjunction with correlation spectroscopy (COSY) and heteronuclear multiple-bond coherence (HMBC) correlations (Fig. S3), compound 3 was determined to be a C-24 bromine-substituted albofungin.

### Effect of halogenation on antibacterial and anticancer activities.

**FIG 6:**
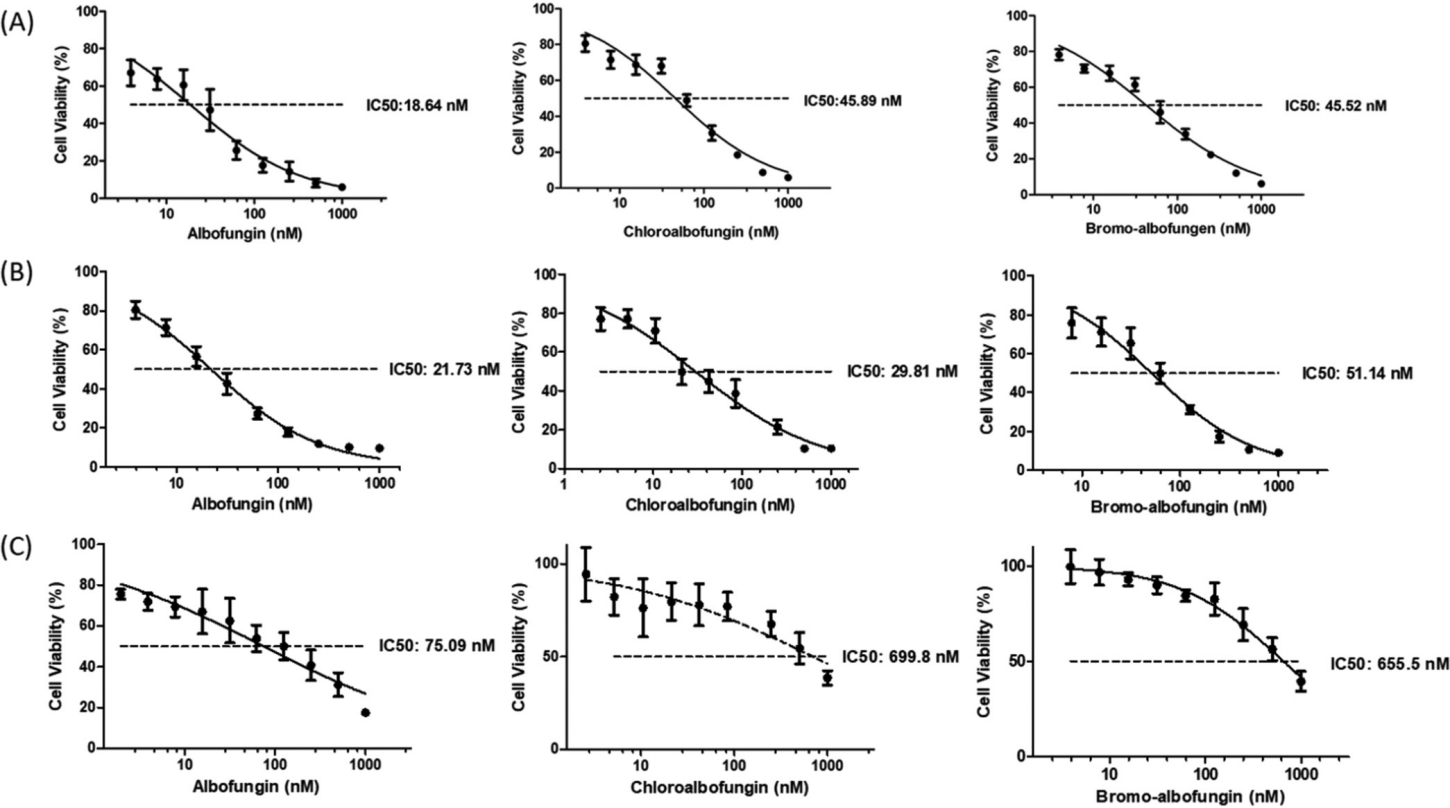
Anticancer activities (IC_50_) of compounds 1 to 3. (A) Bioevaluation of compounds 1 to 3 against the human lung cancer cell line A549. (B) Bioevaluation of compounds 1 to 3 against the human cervical cancer cell line HeLa. (C) Bioevaluation of compounds 1 to 3 against the human colon cancer cell line HCT116.

Compounds 1 to 3 were then subjected to biological examinations to evaluate their antibacterial and anticancer activities, of which the results are shown in Tables 2 and 3 and Fig. 6. All three compounds exhibit strong bactericidal capabilities effective against selected Gram-positive strains at the nanomolar level and selected Gram-negative strains at the micromolar level (Table 2). Of note, compounds 2 and 3 have higher MIC values than compound 1, suggesting that halogenated compounds 2 and 3 are relatively less effective against Gram-negative Klebsiella pneumoniae NTUH-K2044 and Pseudomonas aeruginosa PAO1. Analogously, this diminished bioactivity is also observed in the anticancer assay, where compounds 2 and 3 demonstrate decreased anticancer activity, with a maximum diminution against the human colon cancer cell line HCT116 (Table 3 and Fig. 6C). The pharmacologic effectiveness modulated by the presence of a halogen substituent can be attributed to its electron negativity, steric hindrance, and/or lipophilicity while interacting with the intra- and/or extracellular drug targets (35–37)

**TABLE 2:** MIC of compounds 1 to 3

	Compounds^*a*^		
Bacteria	1	2	3
K. pneumoniae NTUH-K2044	6.125 μM	98 μM	>98 μM
P. aeruginosa PAO1	12.25 μM	>98 μM	>98 μM
S. aureus ATCC 29213	6.125 nM	6.125 nM	12.25 nM
S. aureus ATCC 43300	6.125 nM	12.25 nM	12.25 nM
E. faecalis ATCC 33186	6.125 nM	12.25 nM	12.25 nM
E. faecalis ATCC 51575	6.125 nM	6.125 nM	12.25 nM
*S. albus* J1074::ermE*-*crp*_SC_	196 nM	784 nM	784 nM
WZC-*orfL*	784 nM	6272 nM	6272 nM
WZC-*orfA*	49 nM	784 nM	784 nM

a MIC is defined as the lowest concentration of analyte with no viable growth. Kanamycin was used as a positive control for Gram-negative strains, and vancomycin was used as a positive control for Gram-positive strains.

**TABLE 3:** Anticancer activities (IC_50_) of compounds 1 to 3

	Compounds^*a*^		
Cells	1	2	3
A549 (lung)	18.64 nM	45.89 nM	45.52 nM
Hela (cervix)	21.73 nM	29.81 nM	51.14 nM
HCT116 (colon)	75.09 nM	699.8 nM	655.5 nM

a IC_50_ value is the concentration of the compound resulting in 50% inhibition.

### Function and relationship between OrfA and OrfL.

On the basis of the *in silico* analysis, OrfL is a homologous protein of oligopeptides: H^+^ symporter (*S. chrestomyceticus*, 97.30% sequence identity), a major facilitator superfamily (MFS) peptide transporter (*Streptomyces* sp.,76.85% sequence identity), or a proton-dependent amino acid/oligopeptide transporter (POT; Actinomadura algeriensis, 51.33% sequence identity). These three transmembrane transporters all belong to the MFS superfamily (21). Therefore, OrfL was subjected to analysis with the transporter classification database (TCDB; http://www.tcdb.org) (38), where 21,848 membrane transport proteins were grouped in the MFS family, which is further divided into 16 different families and 89 subfamilies based on their phylogeny and function. OrfL was thereby classified as a POT protein (Fig. S9). MFS proteins are often related to metabolite transportation and anti-multidrug resistance in bacteria (39). In our boundary delimitation for the *abf* BGC (Fig. 2), *orfL* placed at the edge of the upstream boundary has been ascertained to be essential, underlining that OrfL is an indispensable protein likely acting as a transporter in albofungin biosynthesis.

To know whether OrfL acts as an MFS efflux pump to transport or detoxicate albofungin produced in cells, we examined OrfL using a rapid permeability-related susceptibility assay (40, 41), where the MIC was determined for the heterologous expression host *S. albus* J1074::ermE*-*crp*_SC_ and the OrfL-expressed strain *S. albus* J1074::ermE*-*crp*_SC__wzc-*orfL* (WZC-*orfL*) in the presence of a selected compound (compounds 1 to 3). Compared with the control group (the heterologous expression host), WZC-*orfL* displayed significantly increased MIC values for compounds 1 to 3 (4- to 8-fold), agreeing with the assumption that OrfL is a transmembrane transporter toward albofungins (Table 2). The extremely high MIC values measured for the WZC-*orfL* strain treated with compound 2 or 3 suggested that WZC-*orfL* makes the strain relatively less sensitive to halogenated compounds 2 and 3. Moreover, *in vitro* antimicrobial susceptibility examinations showed that *S. albus* J1074::ermE*-*crp*_SC_ is much less sensitive to halogenated albofungins than Staphylococcus aureus and Enterococcus faecalis. Unexpectedly, *in vitro* MIC assays showed that halogenated albofungins are less effective against *S. albus* J1074::ermE*-*crp*_SC_ or *S. albus* J1074::ermE*-*crp*_SC__wzc-*orfA* (WZC-*orfA*) than compound 1; provided compound 1, the MIC of WZC-*orfA* is relatively lower than that of *S. albus* J1074::ermE*-*crp*_SC_, suggesting that halogenation negatively affects the antimicrobial activity of albofungin by interfering with uptake or association with microorganisms. It has been known that reduced permeability toward antibiotics can render antimicrobial drug resistance (42), as antimicrobial drug susceptibility is positively correlated with membrane permeability. OrfL is an MFS efflux pump-like protein presumably pumping out albofungin and the halogenated albofungin in particular produced in the albofungin-producing strains. OrfL can thus be regarded as a drug-detoxicating gene in the producing strain or a drug-resistance gene in an *orfL*-acquired species for self-protection. The decreased drug susceptibility of bacteria treated with halogenated compounds 2 and 3 suggests that OrfA-mediated halogenation intrinsically alters the compounds’ chemical, physical, and biological properties that collectively change their molecular recognition, thereby facilitating OrfL-mediated transportation. To this end, both OrfA and OrfL may exert a “belt and braces” mechanism to safeguard the producing strains from self-toxification.

### Influences of exopolysaccharides on uptake of albofungins.

Extracellular matrix (ECM) is closely related to the formation of biofilm, which plays an important role in the drug resistance of the Gram-positive Staphylococcus species. The major component of the extracellular polymeric substances in the staphylococcal biofilm is the polysaccharide intercellular adhesion (PIA), which has been regularly used to gauge the penetration ability of antibiotics (43, 44). Compounds 1 to 3 were individually examined on S. aureus ATCC 29213, where the MIC values observed under normal conditions were similar to those shown in Table 2, while the values were increased (compound 3 in particular) under biofilm-enhanced conditions (Table 4).

**TABLE 4:** MIC values (nM) of compounds 1 to 3 on PIA formation

	TSB broth			TSB broth + 0.5% glucose		
Bacterium	1	2	3	1	2	3
S. aureus ATCC 29213	12.25	12.25	24.50	49	196	392

**FIG 7:**
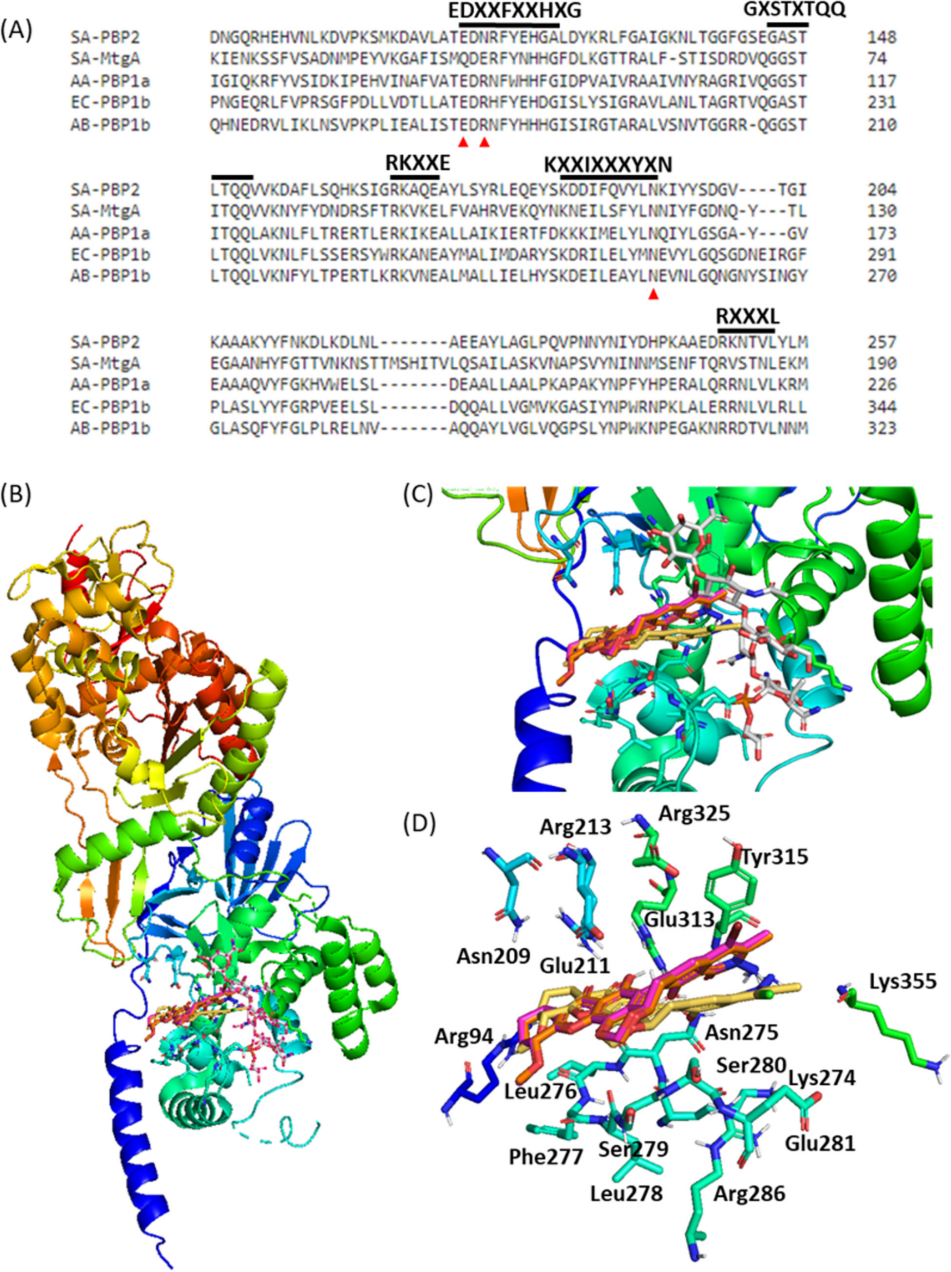
*In silico* simulation of E. coli PBP1b (PDB ID: 3VMA) in complex with albofungins 1 to 3. (A) Conserved motifs are highlighted on the top of the sequence alignment for TGase domains of monofunctional TGase and bifunctional TGase domains of S. aureus MtgA (SA-MtgA), S. aureus PBP2 (SA-PBP2), *A. aeolicus* PBP1a (AA-PBP1a), A. baumannii PBP1b (AB-PBP1b), and E. coli PBP1b (EC-PBP1b). Black bold lines underscore conserved motifs, and red triangles stand for key residues forming hydrogen bonds to albofungins. The multisequence alignment was generated by using ClustalW version 2.0. (B) Superposition of compounds 1 to 3 and moenomycin A, the only antibiotic that binds to the TGase domain in PBP1b. (C) Compounds 1 to 3 and moenomycin A in the active site. (D) Compounds 1 to 3 interact with key active-site residues; gray, moenomycin A; orange, albofungin (compound 1); yellow, chloroalbofungin (compound 2); pink, bromoalbofungin (compound 3). The default analytical distance is set at <5Å for the ligand with its interacting residues. Figures were prepared by using PyMOL.

Structurally, the PIA structure is a polymer composed of repeating units of β(1→6)-*N*-acetylglucosamine, of which biosynthesis is mediated by the *icaADBC* locus (45). In brief, *icaA* encodes an *N*-acetylglucosaminyltransferase that catalyzes the formation of PIA oligomers at the expense of UDP-*N*-acetylglucosamine. IcaD is an accessory protein assisting IcaA to reach optimal efficiency. IcaC is responsible for the externalization of nascent polysaccharides. IcaB is an *N*-deacetylase that partially deacetylates PIA. IcaA in biofilm formation is analogous to TGase in peptidoglycan (PG) formation. There is no significant difference in terms of TGase binding amid albofungin congeners (Fig. 7C), thus suggesting that compounds 1 to 3 have similar antimicrobial activities against Gram-positive strains under general conditions where TGase is accessible as opposed to their ineffectiveness in biofilm formation under conditions where TGase becomes inaccessible.

### Effects of substituted halogen on the interaction between albofungins and TGase.

In terms of MIC values (Table 2), compounds 1 to 3 display various antimicrobial activities against Gram-negative and Gram-positive test strains. In general, all compounds show relatively high antibacterial capabilities toward Gram-positive pathogens. This discrepancy may result from the innate physiological architectures on the cell wall; namely, Gram-negative bacteria contrast themselves from Gram-positive bacteria with a thinner peptidoglycan (PG) cell wall and an additional outer membrane (a semipermeable barrier) (46). Other than unidentified intracellular targets, albofungin (compound 1) was recently reported in a position to associate with the TGase domain of PBPs, which are essential enzymes required for the biosynthesis of PG (2). Moenomycin A (MonA) was the only known naturally occurring antimicrobial that suppresses bacterial growth by targeting TGases (47, 48). In the TGase domain, there are five conserved motifs taking part in MonA binding, as revealed from the complexed structures of monofunctional and bifunctional TGase domains alongside a primary sequence alignment from S. aureus MtgA (SA-MtgA), S. aureus PBP2 (SA-PBP2), Aquifex aeolicus PBP1a (AA-PBP1a), Acinetobacter baumannii PBP1b (AB-PBP1b), and E. coli PBP1b (EC-PBP1b) (Fig. 7A and Fig. S10). Taking advantage of this information, compounds 1 to 3 were docked and optimized in the MonA complexed with PBP1b structure (Protein Data Bank [PDB] ID: 3VMA) for structural examination (Fig. 7B and C). This examination illustrated that the binding site of compounds 1 to 3 is likely situated near the polymerization junction of the peptidoglycan precursors or the MonA-binding site (Fig. 7D and Fig. S11). Compounds 1 to 3 interact mainly with several conserved residues in the binding site of MonA through a constellation of hydrogen bonds in a tight binding manner (Fig. 7A), in agreement with our MIC results that compounds 1 to 3 have similar antibacterial activity against Gram-positive bacteria. The halogen substituent lacks well-defined hydrophilic bonding with nearby residues; instead, it may interact with them through hydrophobic forces. Given the PBP1b-albofungin (compound 1) structural model (Fig. S11), the hydrogen bond interactions between the TGase domain and albofungin (compound 1) are profiled here: the δ-C=O of Glu-211 to 6-OH in 4.1 Å; the δ-OH of Glu-211 to 6-OH and C-8 ketone in 3.1 and 3.1 Å; the C=O of Asn-275 to 3-OH, 6-OH, and 10-OH in 3.6, 3.3, and 2.8 Å, respectively; the γ-NH_2_ of Asn-275 to C-1 ketone, 3-OH, 6-OH, and N-NH_2_ in 2.3 (2.5), 2.5 (3.7), 4.4, and 3.9 (4.7) Å, suggesting that albofungin and its halogenated congeners act equivalently to MonA binding to the donor site of TGases, preventing lipid II from chain polymerization.

The binding affinity between albofungins and TGase was estimated following the approach designed by Wu et al. (2), where TGase-specific inhibiters are captured by TGases that are immobilized on beads by chelation with nickel ions. Having removed unbound compounds, the level of the test compound recovered from the TGase beads should be proportional to its antimicrobial activity. As shown in Table S5, the decreased quantities of bound compounds 1 to 3 are proportional to each individual compound at prepared ranges of concentrations. The recoveries of compounds 1 to 3 have similar leanings. Interestingly, halogenated compounds display a relatively high affinity to TGases at low concentrations likely because of innate hydrophobicity/insolubility of the halogenated congeners. In general, the result of the substrate-ligand interaction assay agreed with that of the *in silico* simulation.

## DISCUSSION

The *abf* BGC in *S. tumemacerans* JCM5050 was identified in this study, where the boundaries of the BGC were precisely delimited by means of CRISPR-Cas9 editing into a minimal but functional DNA fragment of 60 kb. The polycyclic xanthone antibiotics are commonly assembled by core enzymes, including minimal polyketide synthases, cyclases, monooxygenases, and asparagine synthetases, into the given core scaffold, as exemplified by the cases of xantholipin, lysolipin, and albofungin (4, 5, 11). Albofungin makes itself a distinctive polycyclic xanthone as featuring a hydrazine modification, a unique methylation pattern, and a regioselective halogenation, likely in reference to its superb antitumor and antimicrobial functionalities (11). The hydrazine modification is intriguing because of its rarity (49). Three genes *orfDEF* encoding a cupin-like protein, a tRNA synthetase, and an FAD-dependent oxidoreductase alongside *abf6* encoding an l-lysine 6-monooxygenase may together catalyze the modification in a way similar to that in s56-p1 (50). Two transposase-like proteins (*abf10* and *abf11*) identified here imply that the *abf* BGC may be horizontally transferred from other species. Xanthene antibiotics, for example FD-594, pradimicins, benanomicins, MS901809, and BE-13973X, all are attached with sugars (7, 51, 52) but at odds with albofungin that lacks such a modification. The *abf* BCG, somehow, encodes one sugar kinase (*abf13*) and two glycosyltransferases (*abf15* and *abf16*), which are likely relics during the transposase-mediated gene transfer.

Two genes, *orfA* and *orfL*, in the BGC were determined to be related to halogenation and transportation of albofungin derivatives (1–3). OrfA is an FAD-dependent halogenase that transforms albofungin (compound 1) at C-24 to chloroalbofungin (compound 2) or bromoalbofungin (compound 3). ML tree analysis revealed that OrfA is analogous to XanH and LlpH that are specific to xantholipin and lysolipin, respectively, but not albofungin because of low sequence identities (35% to XanH and 34% to LlpH) in reference to OrfA (data not shown). Our biochemical and kinetic examinations showed that OrfA is a two-component FDH halogenase capable of transforming albofungin to halogenated analogs at the catalytic scope. Two-component FDHs normally require FADH_2_ provided by a specific or nonspecific flavin reductase partner to activate molecular oxygen into a C4a-hydroperoxy adduct. As exemplified by the formation of NAI-107, the flavin-dependent halogenase MibH is partnered with the specific NADH-dependent flavin reductase MibS (14). In contrast, there is no such specific partner for OrfA in the *abf* BGC, suggesting that OrfA makes use of endogenous flavin reductases to recruit the necessary FADH_2_. Given that the amino acid sequence of OrfA features both the conserved structural (WXWXIP) and FADH_2_-binding (GXGXXG) motifs of a typical flavin-dependent halogenase, it seems destined for this (53).

The halogenation of a compound may influence its interaction with its binding partners through a variety of ways, for example, altering the overall lipophilicity and/or nonspecific hydrophobic interactions with the N, O, and S atoms of specific/nonspecific targets (54, 55). Our assays demonstrated that halogenated albofungins show relatively less effective antimicrobial activities, likely because ECM diminishes their accessibility to bacteria (Gram-negative bacteria, for example, P. aeruginosa PAO1 and K. pneumoniae NTUH-K2044 in particular; Table 4), which contain relatively high surface polysaccharides (56, 57). PIA also armors staphylococci resistant to some clinically potent antimicrobials (e.g., oxacillin, gentamicin, ciprofloxacin, levofloxacin, cotrimoxazole, erythromycin, vancomycin, and lysostaphin) (43, 58–60). Our assays agreed that S. aureus cultured in different media exhibits a different level of antimicrobial susceptibility to albofungins (Tables 2 and 4), highlighting that the components of the media are critical (61). The increased PIA decreased the susceptibility of S. aureus to albofungins (especially compounds 2 and 3), as they may be trapped or entangled with the ECM by multiple unspecified physical forces, thereby compromising the overall activity.

**FIG 8:**
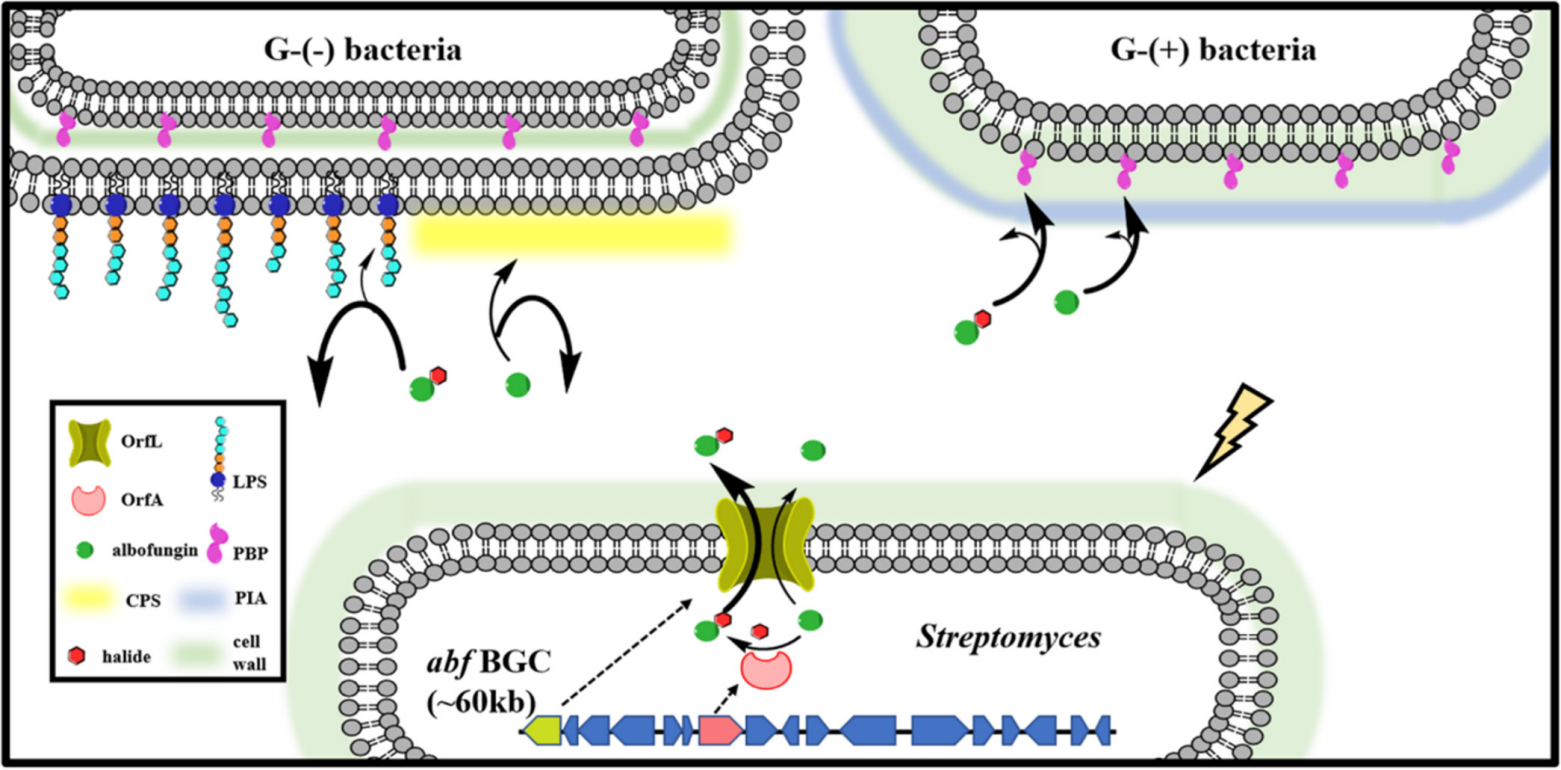
Schematic diagram for the functions of OrfA/OrfL and the influences of extracellular matrices. After triggering by a specific external signal, the biosynthesis of albofungin is turned on, wherein the flavin-dependent halogenase OrfA halogenates albofungin, and the MFS efflux pump-like protein OrfL transports the halogenated products outside the cell in a manner similar to the MFS multidrug efflux pumps reported by Pasqua et al. (39). ECM (e.g., lipopolysaccharide [LPS], PIA, and CPS) acting as an antibiotic physical barrier prevents cell wall damage wreaked by albofungins, especially the halogenated products because of high hydrophobicity and less diffusibility (43, 56, 57).

Bacteria typically use resistance mechanisms, such as limiting drug uptake, modifying cellular targets, inactivating drugs, and exporting toxic compounds, to counter the harmfulness of antimicrobials (62, 63). The MFS transporters are known to export a broad spectrum of nutrients and toxins out of cells. OrfL, an MFS-POT-like protein, does the job to efficiently export albofungins 1 to 3 out of cells. The OrfL-containing species showed that halogenated albofungins, compounds 2 and 3, have high MIC values relative to albofungin, suggesting that the OrfA-mediated halogenation on albofungin (compound 1) facilitates the OrfL-mediated pumping system to favorably export halogen-substituted albofungin and avoid feedback inhibition to biosynthesis as a result of ensemble alteration of albofungin’s chemical and physical properties. The cooperative effect of OrfA and OrfL in the presence of ECM is summarized in Fig. 8.

Compounds 1 to 3 showed encouraging antibacterial and anticancer activities at micromolar to nanomolar ranges. Based on antimicrobial and *in silico* analyses, compounds 1 to 3 fit well into the active site of the TGase domain, in which they may interact with conserved residues through hydrogen-bond networking and hydrophobic interactions (Fig. 7A), as does moenomycin (64–67). The halogen substituent at C-24 of albofungin has little influence on their binding to the cell membrane target TGase, in agreement with compounds 1 to 3 having similar MIC values against Gram-positive microbes in the absence of OrfL.

To this end, this study, for the first time, underscored the importance of chemiosmotic permeability regulation genes encoded in the albofungin biosynthetic gene cluster in *S. tumemacerans* JCM5050. We believe the information revealed herein should enhance our understanding of the biosynthetic logic and mode of action of the pentangular aromatic natural products to a significant extent and should be very conducive to designing and developing new albofungin congeners, for instance, for selectively targeting TGases multidrug-resistant pathogens with improved pharmacodynamics and pharmacokinetics.

## MATERIALS AND METHODS

### General experimental procedures.

DNA amplification was performed using a Bio-Rad C1000 Touch thermal cycler. Protein purification was performed using a GE AKTA pure 25L1 fast-performance liquid chromatographer (FPLC) installed with a Superdex S-200 column. Molecular weight analysis was performed using an LC-TQ-MS or LC-linear trap quadrupole (LTQ)-MS coupled with an Agilent HPLC 1200 system. Compound analysis/separation was performed using a high-performance liquid chromatographer (HPLC) equipped with a UV detector and installed with a Prodigy C_18_ column (250 mm × 4.6 mm, 5 μm) (Phenomenex). Nuclear magnetic resonance (NMR) spectra were recorded using a Bruker AV600 spectrometer (600 MHz) equipped with a three-channel system with a 5-mm triple resonance inverse (TCI) cryoprobe (a ^1^H/^13^C/^15^N triple-resonance probe head with cooled preamplifiers).

### Organisms, plasmids, and media.

Bacterial strains and plasmids used in this study are listed in Tables S1 in the supplemental material. E. coli DH5α and BL21(DE3) competent cells were used for routine cloning and protein expression. *S. albus* J1074::ermE*-*crp*_SC_ (13) was used as the host strain for heterologous expression. *S. albus* J1074::ermE*-*crp*_SC__pmk01 (pMK01) contains the full-length *abf* BGC from *orfL* to *abf58*. BAC clones that contain a pair of coexpressing plasmids from the combination of pSL01 to pSL03 and pN1 to pN6 to delimit the boundaries were constructed. The *orfA*-deficient strain *S. albus* J1074::ermE*-*crp*_SC__pmk01Δ*orfA* (pMK01Δ*orfA*) and two *orfA*-complement strains, *S. albus* J1074::ermE*-*crp*_SC__pmk01Δ*orfA*::*orfA* (pMK01Δ*orfA*::*orfA*) and *S. albus* J1074::ermE*-*crp*_SC__pmk01Δ*orfA*::*orfA*_K144A_ (pMK01Δ*orfA*::*orfA*_K144A_), were prepared for activity examination of OrfA. *S. albus* J1074::ermE*-*crp*_SC__wzc-*orfA* (WZC-*orfA*) and *S. albus* J1074::ermE*-*crp*_SC__wzc-*orfL* (WZC-*orfL*) were constructed for the microbial susceptibility assay. E. coli strains were grown at 37°C on LB agar plates/broth containing appropriate antibiotics, and liquid cultures were incubated on an orbital shaker at 200 rpm. mannitol soya flour (MS) agar and dextrin-soytone-baking yeasts-MOPS (DNPM) liquid/agar were used for the growth and production of albofungin derivatives, respectively (68, 69). Following the standard procedure, the model strain Streptomyces lividans TK64 that was used for protein expression was inoculated into yeast extract-malt extract (YEME) medium and incubated at 28°C (69). Antibiotics used included apramycin (50 μg/mL), neomycin (50 μg/mL), nalidixic acid (25 μg/mL), and thiostrepton (25 μg/mL).

### Genomic DNA isolation, sequencing, and bioinformatic analysis for the *abf* BGC.

The mycelium of *S. tumemacerans* JCM5050 was used for genomic DNA extraction. Briefly, bacteria were resuspended into *N*-tris(hydroxymethyl)methyl-2-aminoethanesulfonic acid (TES) buffer (20 mM Tris-HCl pH 7.5, 5 mM EDTA, and 100 mM NaCl) containing lysozyme and RNase A at 37°C for 30 min, and proteinase K and SDS were added into the bacterial solution at 37°C overnight. The genomic DNA was isolated sequentially by phenol-chloroform extraction and alcohol precipitation. Quantity and quality of extracted gDNA were determined spectrophotometrically. After dissolving gDNA in Tris-EDTA (TE) buffer, we followed the guidelines provided by PacBio for 10-kb template preparation. The gDNA sample was subjected to PacBio RSII circular consensus sequencing. After sequencing, the results were assembled using the PacBio Hierarchical Genome Assembly Process version 2 (HGAP_Assembly.2) (70). The resulting 30 contigs were annotated using antiSMASH to search for cryptic biosynthetic gene clusters.

### Cloning, expression, and purification of OrfA, OrfA_K144A_, E. coli flavin reductase (Fre), and TGase.

Primers used in this study are listed in Table S2. For the expression of OrfA, the gene was amplified from JCM5050 with primer set P901/P902 and cloned into the expression vector pET28a. The recombinant plasmid was amplified by PCR with P549/P550 primers, and the amplicons were introduced into pGM1202 vector, resulting in the pGM1202-His-*orfA* plasmid for protein expression. The resultant plasmid was transformed into S. lividans TK64, and the transconjugants were inoculated into YEME medium containing apramycin for 3 days at 28°C. The culture was transferred to fresh YEME medium for 48 h and induced with thiostrepton. After centrifugation, the harvested cells were resuspended in a binding buffer (50 mM NaH_2_PO_4_ pH 8.0, 300 mM NaCl, 10% glycerol, and 10 mM imidazole) and lysed using a sonicator. After removing the debris by centrifugation, all recombinant proteins were isolated from the supernatant by affinity chromatography at 4°C. The eluted proteins were concentrated and purified within a Superdex S-200 column equilibrated with gel filtration buffer (20 mM HEPES, 150 mM KCl, 1,4-dithiothreitol, pH 8.0). The gene encoding Fre from the genomic DNA of E. coli DH5α was amplified by PCR with the primer set P129/P130 and cloned into pET28a with NdeI and EcoRI restriction sites. The resultant plasmid pET28a-Fre was transformed into E. coli BL21(DE3), in which a single colony was picked and inoculated into LB broth supplemented with kanamycin (35 μg/mL) and induced with 0.5 mM isopropyl β-d-1-thiogalactopyranoside (IPTG) at an appropriate time course. The purification procedure for Fre is similar to the one described above. SDS-PAGE (10%) analysis of OrfA, OrfA_K144A_, E. coli Fre, and TGase from S. aureus ATCC 29213 is showed in Fig. S1. Protein oligomerization of OrfA/OrfA_K144A_ from S. lividans TK64 and OrfA from the E. coli BL21(DE3) chaperon coexpression system was checked using FPLC analysis. In this work, recombinant OrfA and OrfA_K144A_ from S. lividans TK64 were dominantly used for enzymatic assays given high homogeneity of the protein.

### Construction of full-length *abf* BGC for heteroexpression.

To isolate the putative albofungin biosynthetic gene cluster from the chromosome of JCM5050, a thiostrepton resistance gene from the pLUS970 plasmid (71) was amplified using P517 and P518 and introduced into a bacterial artificial chromosome pMKBAC02, which carries the origin of transfer region (*oriT*) and apramycin resistance gene *aacIII*(IV) amplified from plasmid pGUSRolRPA3 (72) using the primer set P325 and P326 and a partial DNA fragment from plasmid pBeloBAC11 (New England Biolabs [NEB]) using primer sets P327 and P328. The resulting recombinant BAC was named pMKBAC02-tsnR (Fig. S2). The DNA fragments containing the partial region of *abf* BGC were amplified with the primer set P498/P499 and cloned into pMKBAC02-tsnR using the PacI restriction enzyme to generate pMKBAC02-tsnR-H. Conjugation was subsequently performed to integrate pMKBAC02-tsnR-H into the chromosomal DNA of *S. tumemacerans* JCM5050 via homologous recombination. Next, the transconjugants were selected on a thiostrepton-containing MS agar plate, as previously described (69), and verified by PCR using the primer set P574/P577. The gDNA of this transconjugant was prepared and digested by the restriction enzyme HindIII. The digested DNA fragments were purified and concentrated by ethanol precipitation before self-ligation using T4 ligase (Thermo Fisher Scientific). The ligation mixture was transferred into 10GBAC-optimized electrocompetent cells (Lucigen) by electroporation. Recombinants were selected on apramycin-containing LB medium agar, after which, plasmids were verified by PCR using the primer set P574/P577. The BAC plasmid carrying the *abf* BGC was identified and named pMKBAC02-tsnR-Albo. The DNA fragment containing the attP-intΦC31 was amplified from the pGUSRolRPA3 plasmid using the primer set P329/P330. The amplicon was digested and cloned into pMKBAC02-tsnR-Albo using the AvrII restriction enzyme for generating pMKBAC02-tsnR-Albo-Int. For replacing the apramycin and thiostrepton resistance genes with the neomycin resistance gene, DNA segments containing the neo (Tn5) phosphotransferase gene amplified from the pNX24 plasmid (73) using the primer set P863/P864 were cloned into the NheI restriction enzyme-digested pMKBAC02-tsnR-Albo-Int plasmid, whereby the resulting BAC was named pmk01 (Table S1).

### Construction of truncated *abf* BGC for boundary determination.

To determine the precise boundaries of *abf* BGC, we constructed pSL01 that carries the *abf* BGC for heterologous expression. In brief, the DNA segments of pmk01 amplified with the primer set P469/P470 were introduced into the SpeI/NsiI-digested pmk01. We also constructed pSL02 and pSL03 BACs by using an *in vitro* RNA-guided Cas9 DNA-editing technique. In brief, the templates for transcription of single guide RNA (sgRNA) targeting at *abf57*, *abf59*, or *abf61* were amplified by overlap extension PCR with a target-specific primer (P389, P386, or P380) and two universal primers (P413 and P414) following the protocol reported previously (74). *In vitro* transcription of sgRNA using the HiScribe T7 quick high-yield RNA synthesis kit (NEB, E2050S) was performed according to the manufacturer’s instructions. The reaction mixture containing Cas9 protein (NEB, M0386S), sgRNA, and pSL01 BAC was prepared and incubated at 37°C for 2 h according to the manufacturer’s protocol. The resulting Cas9-digested DNA was recovered and self-ligated with T4 ligase (NEB, M0202S). BACs pN1 to pN6, were obtained by means of cloning six different segments of *abf* BGC amplified with primer sets P80/P81, P82/P83, P84/P85, P86/P87, P88/P89, P88/P175, or P88/P176 into pMKBAC07, which is a derived pMKBAC02 BAC containing a DNA segment amplified from the pGUSRolRPA3 plasmid using the primer set P329/P330, and six BAC plasmids that respectively contained *abf2* to *abf4*, *orfB* to *abf4*, *orfI* to *abf4*, *orfO* to *abf4*, *orfL* to *abf4*, and *orfK* to *abf4*.

### Construction of the *orfA*-deficient mutant.

The plasmid pCRISPomyces-2 was used for knocking out the *orfA* gene by following the instructions reported previously (75). Briefly, the insertion of a 20-nt spacer (annealing the primer set P877/P878) into the sgRNA scaffold of the pCRISPomyces-2 plasmid was achieved by using Golden Gate assembly. The 1.4-kb left and right homology arms of two editing templates were amplified from purified genomic DNA with primer sets P905/P906 and P907/P908 and ligated into the XbaI site of the selected plasmid after combining the two DNA fragments with overlap extension PCR. Correct plasmid assemblies were selected and confirmed by Sanger sequencing. The resulting plasmid, pC9-DA, was introduced into *S. albus* J1074::ermE*-*crp*_SC_ carrying the pmk01 BAC by conjugation (69). Individual exconjugant was cultured on MS agar plates (2% soy flour, 2% mannitol, and 2% agar) at 37°C, followed by replica plating on selective and nonselective plates for confirming clearance of pC9-DA plasmid. The *orfA*-deficient mutants were identified by PCR with the primer set P909/P910.

### Site-directed mutagenesis.

Site-directed mutagenesis was performed following the manufacturer’s protocol for QuikChange (Stratagene). In brief, the 5′-phosphorylated primers PK144A-F (5′-AACCTGATGCCCAGCGCCCAGGTGGGGCGCA-3′) and PK144A-R (5′-TGCGCCCCACCTGGGCGCTGGGCATCAGGTT-3′) were used for changing the amino acid lysine to alanine (position 144) from the wild-type pGM1202-His-*orfA* plasmid. The resulting mutation was confirmed by Sanger sequencing. The OrfA mutant was expressed and purified with the same protocol used for wild-type protein.

### HPLC-TQ-MS analysis of albofungins 1 to 3.

The products were extracted from cultures with ethyl acetate and dissolved in dimethyl sulfoxide (DMSO) after evaporation. The sample was subjected to HPLC-TQ-MS analysis with a Phenomenex Prodigy C_18_ column (250 mm × 4.6 mm, 5 μm). The mobile phase was set up with a linear gradient of 70% H_2_O + 0.1 formic acid (FA) and 30% acetonitrile (ACN) at a flow rate of 1.0 mL min^−1^ over 30 min. Separation was achieved by a linear gradient from H_2_O + 0.1% FA (A) to ACN + 0.1% FA (B) at a flow rate of 1 mL min^−1^ at 45°C. The gradient was initiated by a 2-min isocratic step at 30% B, followed by an increase to 98% B in 30 min to end up with a 5-min step at 98% B before reequilibration under the initial.

### NMR experiments.

Compounds 1 to 3 were dissolved in dimethyl sulfoxide-*d*_6_ (DMSO-*d*_6_) for ^1^H, ^13^C, COSY, heteronuclear single quantum coherence (HSQC), HMBC, and nuclear Overhauser effect spectroscopy (NOESY) NMR experiments. ^1^H-, ^13^C-NMR chemical shifts of compounds 1 to 3 are tabulated in Table S4, and structural elucidation of compounds 1 to 3 was established on the basis of COSY and HMBC correlations (Fig. S3).

### *In vivo* and *in vitro* enzymatic assay of OrfA.

To examine the halogenation activity of OrfA, we performed halide salt-feeding and *in vitro* enzymatic assays. For the former, four different halide salts, potassium fluoride (KF), potassium chloride (KCl), potassium bromide (KBr), and potassium iodide (KI), were individually added to the fermentation medium of *S. tumemacerans* JCM5050 and *S. albus* J1074::ermE*-*crp*_SC__pmk01Δ*orfA*::*orfA* (pMK01Δ*orfA*::*orfA*). The typical reaction solutions contained E. coli flavin reductase (Fre) and NADH for determining the enzymatic activity of OrfA. To simplify the reaction, 1,4-dithiothreitol (DTT) was used to replace Fre and NADH. Reactions were run in a 400-μL reaction solution (containing 1.5 μM OrfA, 100 μM FAD, 100 mM KCl/KBr, 50 mM DTT, and 8% DMSO in 20 mM potassium phosphate buffer, pH 8.0) in the presence of albofungin (compound 1). The reaction solutions were incubated at 30°C and quenched with butanol. After vaporization of the butanol fraction and redissolving with DMSO, the solutions were injected into HPLC-TQ-MS for analysis.

### Enzyme kinetics of OrfA.

A standard curve of albofungin (compound 1) was prepared on the basis of peak areas of compound 1 at concentrations of 3.125, 6.25, 12.5, 25, 50, 75, and 100 μM at UV_280_. The OrfA-mediated reactions were initiated by adding OrfA into a 400-μL enzymatic reaction solution containing compound 1 at concentrations of 12.5, 25, 50, 75, and 100 μM and quenched at time points of 0, 5, 10, 15, 30, and 60 min by adding 400 μL of butanol. The extracts were analyzed by HPLC. Data analysis and curve fitting were performed by using Prism version 7.04 (GraphPad software). According to Michaelis-Menten kinetics, *V*_max_, *K*_M_, and *k*_cat_ of OrfA were calculated from the Lineweaver-Burk equation, as shown in Fig. S8.

### MIC assay.

Compounds 1 to 3 were assayed for their *in vitro* antibacterial activities against six selected pathogenic bacteria, including two Gram-negative strains, K. pneumoniae NTUH-K2044 and P. aeruginosa PAO1, and four Gram-positive strains, Staphylococcus aureus ATCC 29213, methicillin-resistant S. aureus ATCC 43300, Enterococcus faecalis ATCC 33186, and multidrug-resistant E. faecalis ATCC 51575. MIC was determined according to the Clinical and Laboratory Standards Institute (CLSI) broth microdilution method. Isolated colonies of the tested strains were selected from 18- to 24-h cultured agar and freshly inoculated into broth medium until achieving a turbidity of a 0.5 McFarland standard. Each strain was respectively added with different concentrations of compounds 1 to 3 in 96-well plates. These plates were further incubated at 37°C for 24 h. The MIC endpoint was defined as the lowest compound concentration at which there was no visible growth in the well. In the microbial susceptibility assay, three BAC strains, *S. albus* J1074::ermE*-*crp*_SC,_ WZC-*orfL*, and WZC-*orfA*, were incubated in DNPM medium at 28°C with agitation for 24 h. In the polysaccharide intercellular adhesion (PIA) assay, S. aureus ATCC 29213 was grown in tryptic soy broth (TSB) medium with extra 5% glucose to increase staphylococcal biofilm.

### Cell viability assay.

The alamarBlue cell viability assay (Bio-Rad Laboratories) was performed against selected cell lines, including the human lung cancer cell line A549, the human cervical cancer cell line HeLa, and the human colon cancer cell line HCT116. These cells were cultured in Dulbecco’s modified Eagle’s essential medium (DMEM; Gibco, New York, NY, USA) supplemented with 10% fetal bovine serum (FBS; Invitrogen) and 50 U/mL streptomycin (Gibco, New York, NY, USA) at 37°C and 5% CO_2_. In 96-well plates, 10 × 10^3^ cells were seeded per well for overnight incubation and treated with different concentrations of compounds 1 to 3 for 48 h. Each concentration was performed in triplicate, and the 50% inhibitory concentration (IC_50_) value was analyzed using GraphPad Prism software.

### *In silico* molecular docking by PyRx.

Penicillin-binding proteins (PBPs), consisting of two enzymatic domains transglycosylase (TGase) and transpeptidase (TPase), play crucial roles in bacterial peptidoglycan (PG) biosynthesis essential for cell growth and division. Several crystal structures of apo-PBPs and PBP in complex with various substrates are available from the RCSB Protein Data Bank (PDB). In this study, E. coli PBP1b (PDB ID: 3VMA) was used in the *in silico* molecular-docking simulation. The molecular-docking studies were performed by using PyRx 0.8 software, a suite of open-source software, including AutoDock 4.2 and AutoDock Vina (docking software), AutoDock Tools (generating input files), and Open Babel (importing spatial data file [SDF] files, removing salts, and energy minimization). Compounds 1 to 3 were docked against PBP1b, where the protein was kept rigid during docking calculation. Best conformations and corresponding binding affinities were rendered on the basis of the default scoring system, where the distance of ligands with interacting residues was set at <5 Å. Protein and ligands were visualized and illustrated using the PyMOL molecular graphics system, version 2.0 (https://pymol.org/2/; Fig. S9).

### Quantification of ligand-TGase interaction by affinity-based ligand-screening methods.

Immobilized TGase was prepared following the previously reported method (2). In short, the DNA fragment containing the TGase domain was amplified with the primer set P571/P572 from the genomic DNA of S. aureus ATCC 29213 and cloned into the expression vector pET28a (Novagen). The plasmids were transformed into E. coli BL21(DE3), and protein expression was induced by adding 0.1 mM IPTG overnight at 16°C with vigorous shaking. Bacterial cells were resuspended in binding buffer (50 mM HEPES pH 7.4, 500 mM NaCl, and 10 mM imidazole) and lysed by a microfluidizer. After removing cell debris by centrifugation, the supernatant was directly immobilized onto nickel chelation beads. Immobilization was done by incubation of protein extracts with nickel chelation beads for 1 h at 4°C with gentle agitation. The immobilized TGase protein was confirmed by SDS-PAGE and stored in storage buffer (50 mM HEPES, pH 7.4) at 4°C. For quantifying the interaction between ligands and TGase, various concentrations (from 2 to 0.05 μM) of compounds 1 to 3 were mixed with the slurry (50 mM HEPES, pH 7.0, and 10% DMSO) containing 0.4 nmol of immobilized TGase. After incubation at 25°C for 1 h with vigorous shaking, beads were collected and washed three times with a buffer solution (50 mM HEPES, pH 7.0) to remove unbound molecules. Subsequently, the bound molecules were released by adding methanol to the beads, and the mixture was shaken for 10 min. The supernatant was dried and suspended in DMSO; the recovery yield of compounds was quantified by antibacterial activity determination compared with the MIC values of pure standard compounds.

### Data availability.

The nucleotide sequence data of the albofungin gene cluster identified in this study have been deposited in the GenBank database with the GenBank accession number ON399210.
