# Supplementary material for: Genetic and Biochemical Characterization of Halogenation and Drug Transportation Genes Encoded in the Albofungin Biosynthetic Gene Cluster
Source: Appl Environ Microbiol. 2022 Aug 24;88(17):e00806-22. doi: 10.1128/aem.00806-22 (PMC9469721; doi:10.1128/aem.00806-22)
Supplement: Supplemental file 1 — Supplemental material. Download aem.00806-22-s0001.pdf, PDF file, 6.8 MB [file aem.00806-22-s0001.pdf]

Supplemental Material

Genetic and biochemical characterization of halogenation and drug-transportation genes encoded in the albofungin biosynthetic gene cluster

Zhe-Chong Wang[a†], I-Wen Lo[a†], Kuan-Hung Lin[a], An Ning Cheng[a], Saeid Malek Zadeh[a], Yen-Hua Huang[b], Tsung-Lin Li[a,c,#]

[a] Genomics Research Center, Academia Sinica, Taipei 115, Taiwan

[b] Institute of Biomedical Informatics, National Yang Ming Chiao Tung University, Taipei 112, Taiwan

[c] Biotechnology Center, National Chung Hsing University, Taichung City 402, Taiwan

KEYWORDS: albofungin, chloroalbofungin, bromoalbofungin, halogenase, biosynthetic gene cluster, FAD-dependent flavoenzyme, transporter

[#]Address correspondence to Tsung-Lin Li, tlli@gate.sinica.edu.tw

[†] These two authors contributed equally to this work. Zhe-Chong Wang and I-Wen Lo implemented respectively the genetic and biochemical portions of the work.

Table S1. Strains and plasmids used in this study.

Bacterial strains and plasmids	Properties	Source or reference
E. coli DH5α	Competent cells for routine cloning	Thermo Fisher
E. coli BL21(DE3)	Competent cells for routine cloning	Thermo Fisher
10GBAC	Construction of high molecular weight BAC	Lucigen
E. coli ET12567	DNA methylation deficient strain	(1)
E. coli ET12567[pUZ8002]	Conjugative transfer of DNA	(1)
S. tumemacerans JCM5050	Albofungin producing strain	JCM Resources
S. albus J1074::erm*-*crp*$_{SC}$	Heterologous expression host	(2)
pMK01	*S. albus* J1074::erm*-*crp*$_{SC}$ carrying pmk01	This study
WZC-*orfL*	*S. albus* J1074::erm*-*crp*$_{SC}$ carrying wzc-*orfL*	This study
WZC-orfA	*S. albus* J1074::erm*-*crp*$_{SC}$ carrying wzc-*orfA*	This study
pMK01Δ*orfA*	*orfA* gene-deficient mutant of pMK01	This study
pMK01Δ*orfA*::*orfA*	pMK01Δ*orfA* carrying wzc-*orfA*	This study
pMK01Δ*orfA*::*orfA*$_{K144A}$	pMK01Δ*orfA* carrying wzc-*orfA*$_{K144A}$	This study
S. lividans TK64	Protein expression host	ATCC Resources
K. pneumoniae NTUH-K2044	MIC assay	(3)
P. aeruginosa PAO1	MIC assay	ATCC Resources
S. aureus ATCC 29213	MIC assay	ATCC Resources
S. aureus ATCC 43300	MIC assay	ATCC Resources
E. faecalis ATCC 33186	MIC assay	ATCC Resources
E. faecalis ATCC 51575	MIC assay	ATCC Resources
pET28a (+)	T7 vector for *E. coli* BL21(DE3)	Novagen
pBeloBAC11	Single-copy *E. coli* plasmid vector used for constructing *E. coli-Streptomyces* shuttle BAC	New England Biolabs (NEB)
pGUSRolRPA3	Promoter probe vector containing the *gusA* gene under the control of PA3-*rolO* promoter	(4)
pLUS970	*E. coli*–*Streptomyces* shuttle vector for the expression of genes	(1)
pNX24	Plasmid containing the promoter of *xysA* gene for expression in Streptomycetes	(5)
pMKBAC02	BAC for carrying truncated *abf* BGC segment	This study
pMKBAC02-tsnR	Modified BAC for capturing *abf* BGC	This study
pMKBAC02-tsnR-H	BAC for capturing *abf* BGC	This study
pMKBAC07	BAC for carrying truncated *abf* BGC segment	This study
pmk01	BAC carrying *abf* BGC segment *orfX*–*abf61*	This study
pSL01	BAC carrying *abf* BGC segment *abf5*– *abf61*	This study
pSL02	BAC carrying *abf* BGC segment *abf5*–*abf58*	This study

pSL03	BAC carrying *abf* BGC segment *abf5*–*abf56*	This study
pN1	BAC carrying *abf* BGC segment *abf2*–*abf4*	This study
pN2	BAC carrying *abf* BGC segment *orfB*–*abf4*	This study
pN3	BAC carrying *abf* BGC segment *orfI*–*abf4*	This study
pN4	BAC carrying *abf* BGC segment *orfO*–*abf4*	This study
pN5	BAC carrying *abf* BGC segment *orfL*–*abf4*	This study
pN6	BAC carrying *abf* BGC segment *orfK*–*abf4*	This study
pC9-DA	Construction of *orfA*-deficient mutant	This study
pGM1202	Vector for gene expression in *Streptomyces*	Addgene# 69615
pGM1202-*orfA*	For His_6-tagged OrfA expression	This study
pGM1202-$orfA_{K144A}$	For His_6-tagged $OrfA_{K144A}$ expression	This study
wzc-*orfL*	Plasmid for expressing *orfL* in *Streptomyces*	This study
wzc-*orfA*	Plasmid for expressing *orfA* in *Streptomyces*	This study
wzc -$orfA_{K144A}$	K144A mutant of wzc-*orfA*	This study
pET28a-*Fre*	For His_6-tagged Fre expression	This study
pET28a-*TGase*	For His_6-tagged TGase expression	This study

Table S2. Oligonucleotide primers used in this study.

Primers	Sequence 5'–3'
P80	TCCCTTCTCGTGGAGCGTGCTGAACA
P81	ATTATTATTCATATGTCAGTCCTGGCAGGCGGCCC
P82	ATTATTATTCATATGGGGCCGCCTGCCAGGACTGA
P83	ATTATTATTCAATTGTTACAGGTCGAGCGTGATGCCGTAGTG
P84	ATTATTATTCAATTGCACTACGGCATCACGCTCGACCTGTAA
P85	ATTATTATTTTAATTAACTACTGCGGAGCCTTCTGGTAGGC
P86	ATTATTATTTTAATTAAGCCTACCAGAAGGCTCCGCAGTAG
P87	ATTATTATTTTAATTAAGAGGGTTTCACCAGTCGATGACCGTGT
P88	ATTATTATTGGATCCACACGGTCATCGACTGGTGAAACCCTC
P89	ATTATTATTGGATCCGGTCTGCTGCAACTTCTTGAGCGCGAA
P129	ATTATTAAACATATGACAACCTTAAGCTGTAAAGTGACCTCGG
P130	ATTATTAAAGAATTCTCAGATAAATGCAAACGCATCGCCAAACAGG
P175	ATTATTATTCATATGAACGAGTTCTGTGTCGACTGAAGGCTCGCCGA
P176	ATTATTATTCATATGATCTGGACGCTCATGTAGAAGAGGGCGAA
P325	ATTACCACCTTAATTAAGGATCCGAATTCGAAGATCCTTTGATCTTTTC
P326	ATTATTATTCCTAGGAGCTTGCATGCCTGCAGGTCGA
P327	ATTATTATTCCTAGGGTTTAAACAGGGCTTCCCGGTATCAAC
P328	TTACCACCTTAATTAAAAGCTTGGTTACTCCGTTCTACAGGTTAC
P517	ATTATTATTGCTAGCGGGGATCGACCGCGCGGGT
P518	ATTATTATTGCTAGCTGATCATCACTGACGAATCGAGGTCGAGGAAC
P498	ATTATTACCTTAATTAAAGTGTCGCACCCGAAGACGTATAAGTGTTTAT
P499	ATTATTACCTTAATTAAACGCTCATGGACCAGCGCACTCAATAA
P574	TGCACATGAACCAAAAGGATCTAGGTGAAGATCCTTTTTGATAA
P577	CTACGTGACGGAGGGAGTGAACAGGAACAA
P329	ATTATTATTCCTAGGCTAGCGATTCCAGACGTCCCGAAG
P330	ATTATTATTCCTAGGAATTCCCCAATGTCAAGCACTTCCGG
P380	GATCACTAATACGACTCACTATAGGCGCGCCGGCTGTGTCGGGCGTTTTAGAGCTAGAAATA GCAA
P386	GATCACTAATACGACTCACTATAGGCGGCGGCGTGGCCACGGAGGTTTTAGAGCTAGAAATA GCAA
P389	GATCACTAATACGACTCACTATAGACCGCGGGTGGTAAGGGAAGGTTTTAGAGCTAGAAATA GCAA
P413	GTTTTAGAGCTAGAAATAGCAAGTTAAAATAAGGCTAGTC
P414	AAAAGCACCGACTCGGTGCCACTTTTTCAAGTTGATAACGGACTAGCCTTATTTTAACT
P571	ATTATTCATATGTATGCTTGGAAAGCACCTGCTTTTAC
P572	ATTATTCTCGAGTTATTCTTCAGGAGTACGATTTACTAAGTTCG

P863	ATTATTATTTCTAGAGAAGATCCTTTGATCTTTTCTACGGGGTCTGA
P864	ATTATTATTTCTAGACTAGCGATTCCAGACGTCCCGAAGG
P877	ACGCCCGAACAGCCCGAAGAAGGA
P878	AAACTCCTTCTTCGGGCTGTTCGG
P901	ATTATTATTATTATTAATATGGACCACAAGGTACTGAACAGCGGGC
P902	ATTATTATTATTCTCGAGCCACATCCCCATCCGCCGCGC
P905	ATTATTATTTCTAGATACTGGTAGGTGGTGCCGATATTTCCTCCTT
P906	ATGACGCTCTTCCTCATCGACTACCTGCTGCTGGGCCAGCA
P907	AGCAGGTAGTCGATGAGGAAGAGCGTCATGTACGTCACGGT
P908	ATATTAATATCTAGACGTCCTCGTGGTCGTTGGAGTAGTTCT
P909	TTCAGCATGTCCTGCACGAAGGCGTT
P910	TGGAAGCTGGGCATCAGGTTCGACT

Table S3. Deduced functions of ORFs in the minimal *abf* BGC from *S. tumemacerans* JCM5050.

**proteins (aa)**	**Putative product**	**Homologs**	**Identities**
OrfL (501)	proton-dependent oligopeptide transporter	WP_206505980.1	484/501(97%)
OrfK (107)	antibiotic biosynthesis monooxygenase	WP_031001543.1	107/107(100%)
OrfJ (236)	putative transcriptional regulator	WP_206505981.1	151/168(90%)
OrfI (227)	short-chain dehydrogenase	WP_206505983.1	214/227(94%)
OrfH (126)	hypothetical protein	WP_050510192.1	118/126(94%)
OrfG (460)	FAD-linked oxidase	WP_206505984.1	435/460(95%)
OrfF (369)	L-lysine 6-monooxygenase	WP_206505985.1	340/369(92%)
OrfE (457)	methionyl-tRNA synthetase	WP_206505986.1	426/457(93%)
OrfD (116)	cupin	WP_206505987.1	110/116(95%)
OrfC (171)	isochorismatase	WP_206505988.1	149/159(94%)
OrfB (518)	hypothetical protein	WP_206505989.1	454/518(88%)
OrfA (583)	tryptophan halogenase	WP_206505990.1	543/582(93%)
Abf1 (452)	glutamate-ammonia ligase	WP_206505991.1	437/452(97%)
Abf2 (614)	asparagine synthase	WP_206505992.1	608/621(98%)
Abf3 (232)	putative transcriptional regulator	WP_206505993.1	214/232(92%)
Abf4 (527)	2,4-dichlorophenol 6-monooxygenase	WP_206505994.1	511/527(97%)
Abf5 (337)	methyltransferase	WP_206505995.1	328/337(97%)
Abf6 (508)	lysine N6-hydroxylase	WP_206505996.1	422/442(95%)
Abf7 (217)	acetoacetyl-CoA reductase	WP_206505997.1	209/216(97%)
Abf8 (404)	monooxygenase	WP_206506268.1	376/404(93%)
Abf9 (284)	putative F420-dependent reductase	WP_206505998.1	276/284(97%)
Abf10 (31)	transposase-like protein	WP_096624216.1	31/31(100%)
Abf11 (80)	transposase IS4 family protein	WP_238545538.1	62/102(61%)
Abf12 (260)	methyltransferase	MBO0829390.1	134/257(52%)
Abf13 (313)	sugar kinase	KOT91395.1	196/296(66%)
Abf14 (33)	hypothetical protein	WP_211275163.1	22/33(67%)
Abf15 (175)	glycosyltransferase	WP_206505999.1	163/175(93%)
Abf16 (226)	UDP-glucuronosyltransferase	WP_206505999.1	214/226(95%)
Abf17 (287)	3-hydroxyisobutyrate dehydrogenase	WP_206506000.1	272/287(95%)
Abf18 (108)	heme-degrading monooxygenase	WP_031001509.1	98/103(95%)
Abf19 (107)	antibiotic biosynthesis monooxygenase	WP_031001507.1	101/107(94%)
Abf20 (250)	short-chain dehydrogenase	WP_206506001.1	237/250(95%)
Abf21 (151)	putative monooxygenase	WP_206506002.1	134/139(96%)
Abf22 (153)	polyketide cyclase	WP_031001503.1	144/153(94%)
Abf23 (410)	Minimal PKS chain-length factor	WP_206506003.1	387/410(94%)

Abf24 (194)	type II PKS ketosynthase alpha subunit	WP_206506004.1	184/194(95%)
Abf25 (274)	3-oxoacyl-(Acyl-carrier-protein) synthase	WP_206506004.1	225/227(99%)
Abf26 (144)	putative monooxygenase	WP_206506005.1	139/144(97%)
Abf27 (111)	polyketide synthase	WP_031001497.1	104/111(94%)
Abf28 (132)	SchA/CurD	WP_031001496.1	124/131(95%)
Abf29 (141)	putative membrane protein	WP_031001495.1	110/115(96%)
Abf30 (129)	SchA/CurD	WP_031001493.1	126/129(98%)
Abf31 (237)	short-chain dehydrogenase	WP_206506006.1	232/237(98%)
Abf32 (89)	phosphopantetheine-binding protein	WP_031001489.1	88/89(99%)
Abf33 (274)	AfsR family transcriptional regulator	WP_206506007.1	269/274(98%)
Abf34 (253)	thioesterase	WP_206506269.1	203/225(90%)
Abf35 (285)	4'-phosphopantetheinyl transferase	WP_206506270.1	255/270(94%)
Abf36 (87)	type I polyketide synthase	WP_206506008.1	81/87(93%)
Abf37 (514)	type I polyketide synthase component	WP_206506008.1	371/418(89%)
Abf38 (72)	hypothetical protein	WP_206506009.1	66/72(92%)
Abf39 (196)	hypothetical protein	WP_206506010.1	187/224(83%)
Abf40 (71)	Acyl-CoA carboxylase subunit epsilon	WP_206506011.1	58/71(82%)
Abf41 (579)	polyketide synthase	WP_206506012.1	565/579(98%)
Abf42 (413)	hypothetical protein	WP_206506013.1	375/413(91%)
Abf43 (204)	DNA-binding response regulator	WP_206506014.1	201/204(99%)
Abf44 (323)	histidine kinase	WP_206506015.1	308/322(96%)
Abf45 (264)	regulatory protein	WP_206506016.1	256/264(97%)
Abf46 (529)	methylmalonyl-CoA carboxyltransferase	WP_206506017.1	504/529(95%)
Abf47 (260)	hydroxyneurosporene-O-methyltransferase	WP_206506018.1	249/260(96%)
Abf48 (48)	hypothetical protein	WP_206506018.1	23/28(82%)
Abf49 (336)	carminomycin 4-O-methyltransferase	WP_206506019.1	315/336(94%)
Abf50 (399)	cytochrome P450	WP_206506020.1	389/402(97%)
Abf51 (80)	ferredoxin	WP_050510183.1	77/80(96%)
Abf52 (102)	transcriptional regulator	WP_238783483.1	101/102(99%)
Abf53 (65)	transcriptional regulator, MarR family	WP_063759224.1	36/37(97%)
Abf54 (54)	hypothetical protein	WP_206506021.1	41/44(93%)
Abf55 (126)	MarR family transcriptional regulator	WP_206506021.1	123/126(98%)
Abf56 (254)	NAD(P)-dependent dehydrogenase	WP_031001458.1	245/254(96%)
Abf57 (109)	FAD-binding monooxygenase	WP_206506022.1	75/77(97%)
Abf58 (350)	FAD-binding monooxygenase	WP_206506022.1	337/350(96%)

Table S4. ^{1}H-NMR (600 MHz) spectroscopic data of albofungins **1**−**3**. [a]

No.	**1** δ_H (mult, *J* in Hz)	**2**	**3**
10	4.82 (br t, 6.0)	4.83 (br dd, 7.7, 3.8)	4.83 (br dd, 7.9, 3.7)
11	1.81 (ddd, 13.8, 7.8, 3.5)	1.81 (m)	1.82 (m)
	1.71 (m, overlapping)	1.73 (m)	1.74 (m)
12	2.05 (dd, overlapping)	2.05 (m)	2.07 (overlapping)
	2.05 (dd, overlapping)	2.05 (m)	2.07 (overlapping)
13	4.43 (dd, 8.7, 6.8)	4.43 (br d, 6.8)	4.45 (dd, 8.6, 7.0)
14			
16			
17			
18			
19	4.96 (dd, 13.0, 4.6)	4.99 (dd, 13.1, 4.7)	5.00 (dd, 13.1, 4.7)
20	3.22 (dd, 13.0, 4.6)	3.37 (dd, 13.1, 4.7)	3.38 (overlapping)
	2.75 (t, 13.0)	2.82 (t, 13.1)	2.82 (t, 13.1)
21			
22	7.01 (s)	7.33 (s)	7.32 (s)
23			
24	6.61 (s)		
25			
26	2.44 (s)	2.66 (s)	2.74 (s)
27	3.55 (s)	3.56 (s)	3.57 (s)
28	5.60 (d, 5.9)	5.62 (d, 5.9)	5.63 (d, 5.9)
	5.42 (d, 5.9)	5.43 (d, 5.9)	5.44 (d, 5.9)
3-OH	13.57 (s)	13.76 (s)	13.82 (s)
6-OH	12.94 (s)	13.00 (s)	13.00 (s)
10-OH	5.14 (br s)	5.15 (d, 4.7)	5.15 (d, 4.6)
N-NH_2	5.90 (br s)	6.01 (s)	6.04 (s)

[a] Coupling constants (*J*) in Hz were given in parentheses. The assignments were determined by ^{1}H, ^{13}C, COSY, HMQC, and HMBC NMR spectra.

Table S5. ^{13}C-NMR (150 MHz) spectroscopic data of albofungins **1**−**3**.[a]

No.	**1** δ_C (type)	**2**	**3**
1	163.3 (C)	162.7 (C)	162.2 (C)
2	109.2 (C)	108.7 (C)	108.8 (C)
3	156.8 (C)	157.1 (C)	158.0 (C)
4	112.8 (C)	114.6 (C)	114.4 (C)
5	109.8 (C)	109.1 (C)	109.8 (C)
6	149.5 (C)	149.8 (C)	149.7 (C)
7	111.6 (C)	111.0 (C)	111.2 (C)
8	182.1 (C)	182.1 (C)	182.1 (C)
9	120.3 (C)	120.4 (C)	120.5 (C)
10	58.7 (CH)	58.8 (CH)	58.8 (CH)
11	27.9 (CH_2)	27.9 (CH_2)	27.9 (CH_2)
12	22.8 (CH_2)	22.8 (CH_2)	22.8 (CH_2)
13	74.7 (CH)	74.3 (CH)	74.5 (CH)
14	165.2 (C)	165.3 (C)	165.3 (C)
16	142.7 (C)	143.0 (C)	143.0 (C)
17	130.3 (C)	130.4 (C)	130.5 (C)
18	130.1 (C)	130.1 (C)	130.2 (C)
19	72.1 (CH)	71.9 (CH)	72.4 (CH)
20	35.9 (CH_2)	36.0 (CH_2)	36.4 (CH_2)
21	140.4 (C)	141.7 (C)	141.6 (C)
22	113.8 (CH)	111.5 (CH)	114.2 (CH)
23	136.3 (C)	133.6 (C)	134.6 (C)
24	105.2 (CH)	109.9 (C)	100.2 (C)
25	141.7 (C)	139.8 (C)	141.2 (C)
26	19.0 (CH_3)	16.3 (CH_3)	20.3 (CH_3)
27	57.8 (CH_3)	57.9 (CH_3)	57.9 (CH_3)
28	90.6 (CH_2)	90.7 (CH_2)	90.7 (CH_2)

[a] Carbon type were given in parentheses. The assignments were determined by ^{1}H, ^{13}C, COSY, HMQC, and HMBC NMR spectra.

Table S6. Recovery screening of immobilized TGase from *S. aureus* ATCC 29213[a]

Compound	Concentration (μM)	Recovery Concentration (nmol)
**1**	2.0	0.1176
	0.8	0.1176
	0.4	0.0588
	0.2	0.0294
	0.1	0.0147
	0.05	<0.0147
**2**	2.0	0.1176
	0.8	0.1176
	0.4	0.0588
	0.2	0.0588
	0.1	0.0147
	0.05	<0.0147
**3**	2.0	0.1176
	0.8	0.1176
	0.4	0.0588
	0.2	0.0588
	0.1	0.0147
	0.05	<0.0147

[a]TGase: 0.4 (nmol)

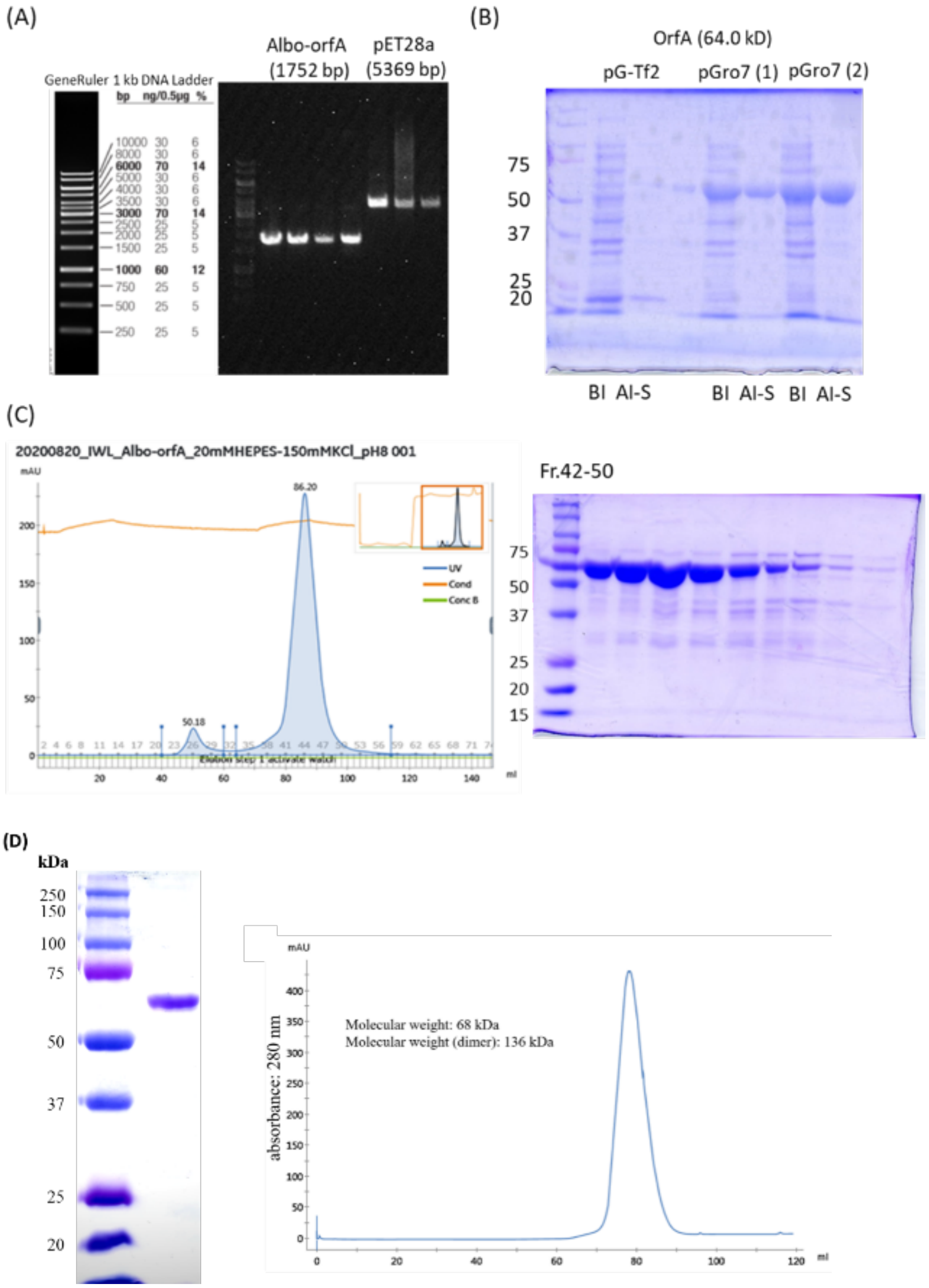
(A)
Albo-orfA
(1752 bp)
pET28a
(5369 bp)
GeneRuler 1 kb DNA Ladder
bp ng/0.5μg %
10000 30 6
8000 30 6
6000 70 14
5000 30 6
4000 30 6
3500 30 6
3000 70 14
2500 25 5
2000 25 5
1500 25 5
1000 60 12
750 25 5
500 25 5
250 25 5
(B)
OrfA (64.0 kD)
pG-Tf2
pGro7 (1)
pGro7 (2)
75
50
37
25
20
BI AI-S
BI AI-S
BI AI-S
(C)
20200820_IWL_Albo-orfA_20mMHEPES-150mMKCl_pH8 001
mAU
86.20
50.18
UV
Cond
Conc B
ml
Fr.42-50
75
50
37
25
20
15
(D)
kDa
250
150
100
75
50
37
25
20
mAU
absorbance: 280 nm
Molecular weight: 68 kDa
Molecular weight (dimer): 136 kDa
ml

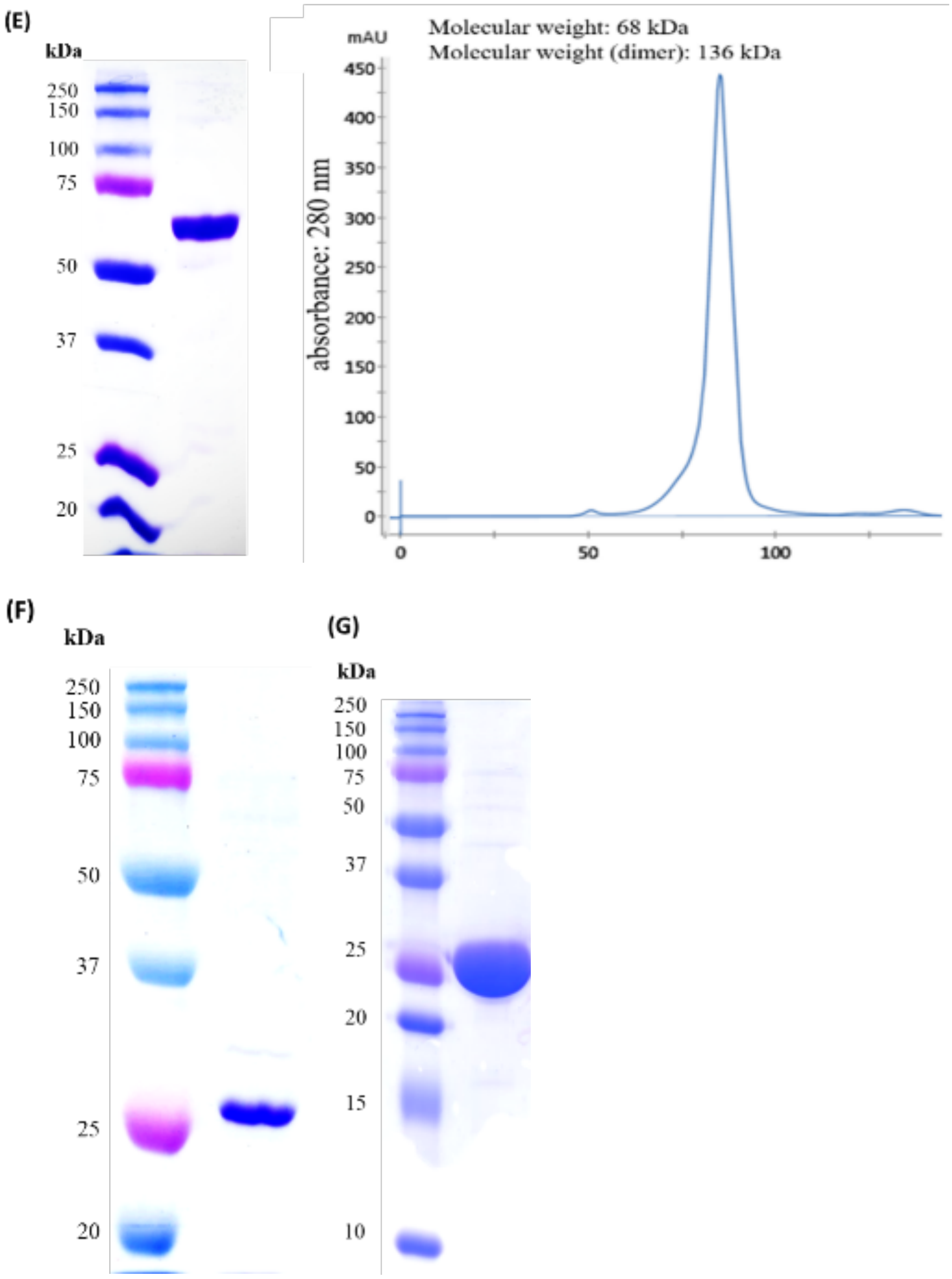

FIG S1. Construction of His$_6$-tagged OrfA. (A) Agarose gel of *orfA* amplicons and restriction enzyme digested pET28a vectors. (B) SDS-PAGE of His$_6$-tagged OrfA by *E. coli* BL21(DE3)-chaperone (groES/groEL) system. BI, protein expression before IPTG induction; AI-S, the supernatant of protein expression after IPTG induction. (C) FPLC (left) of His$_6$-tagged OrfA from the *E. coli* BL21(DE3) chaperone co-expression (groES/groEL) system and SDS-PAGE (right) of fractions 42–50. (D) SDS-PAGE (left) and FPLC (right) of the His$_6$-tagged OrfA from *S. lividans* TK64 [pGM1202-*orfA*]. (E) SDS-PAGE (left) and FPLC (right) of the His$_6$-tagged OrfA$_{K144A}$ (an inactive OrfA) from *S. lividans* TK64 [pGM1202-*orfA*$_{K144A}$]. (F) SDS-PAGE of the His$_6$-tagged TGase of *S. aureus* ATCC 29213 from *E. coli* BL21(DE3). (G) SDS-PAGE of the His$_6$-tagged Fre protein from *E. coli* BL21(DE3). DNA

ladder: GeneRuler 1kb (Thermo Scientific); Protein ladder: Precision Plus Protein^{TM} Dual Color standards (Bio-Rad).

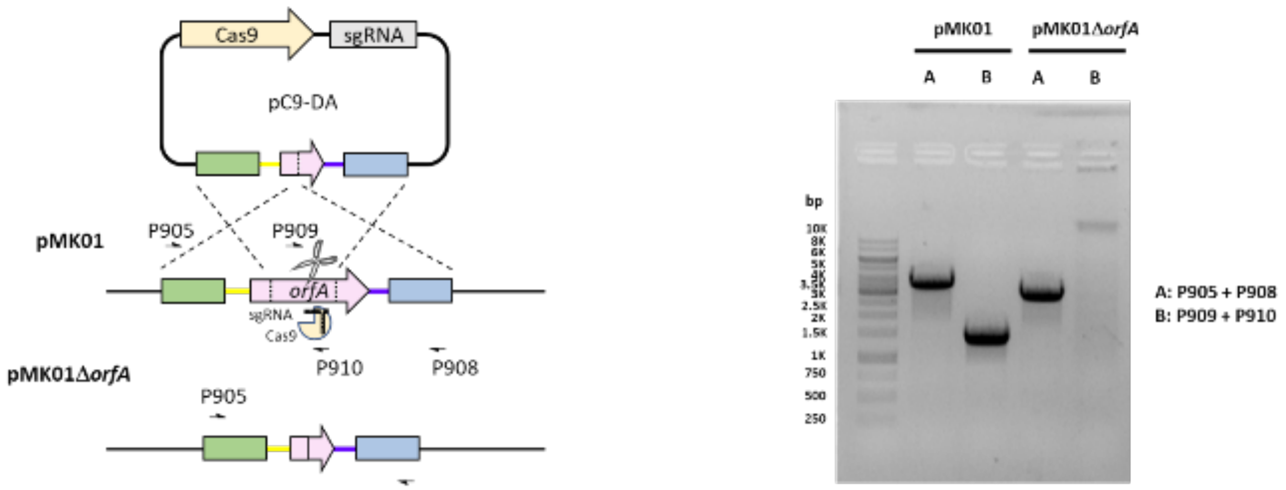

FIG S2. Construction of pMK01Δ*orfA* mutant strains used in this study. (A) Scheme for the construction of *orfA*-deficient mutants using pC9-DA plasmids via *in vivo* CRISPR-Cas9 genome editing and homology directed repairing. P905/P908/P909/P910 are primers used for verifying the mutants. (B) Amplifying DNA fragments from the genome of *S. albus* J1074::erm*-crp_{SC} integrated with pMK01 or pMK01Δ*orfA* BAC and analyzing the amplicons with 1% agarose gel. Amplicons of 3.8 and 2.7 kb were produced from the heterologous host carrying pMK01 and pMK01Δ*orfA* BAC using primer sets P905/P908, respectively. 1.2-kb PCR fragments and no specific amplicons were produced from pMK01 and mutant strain individually using primer sets P909/P910.

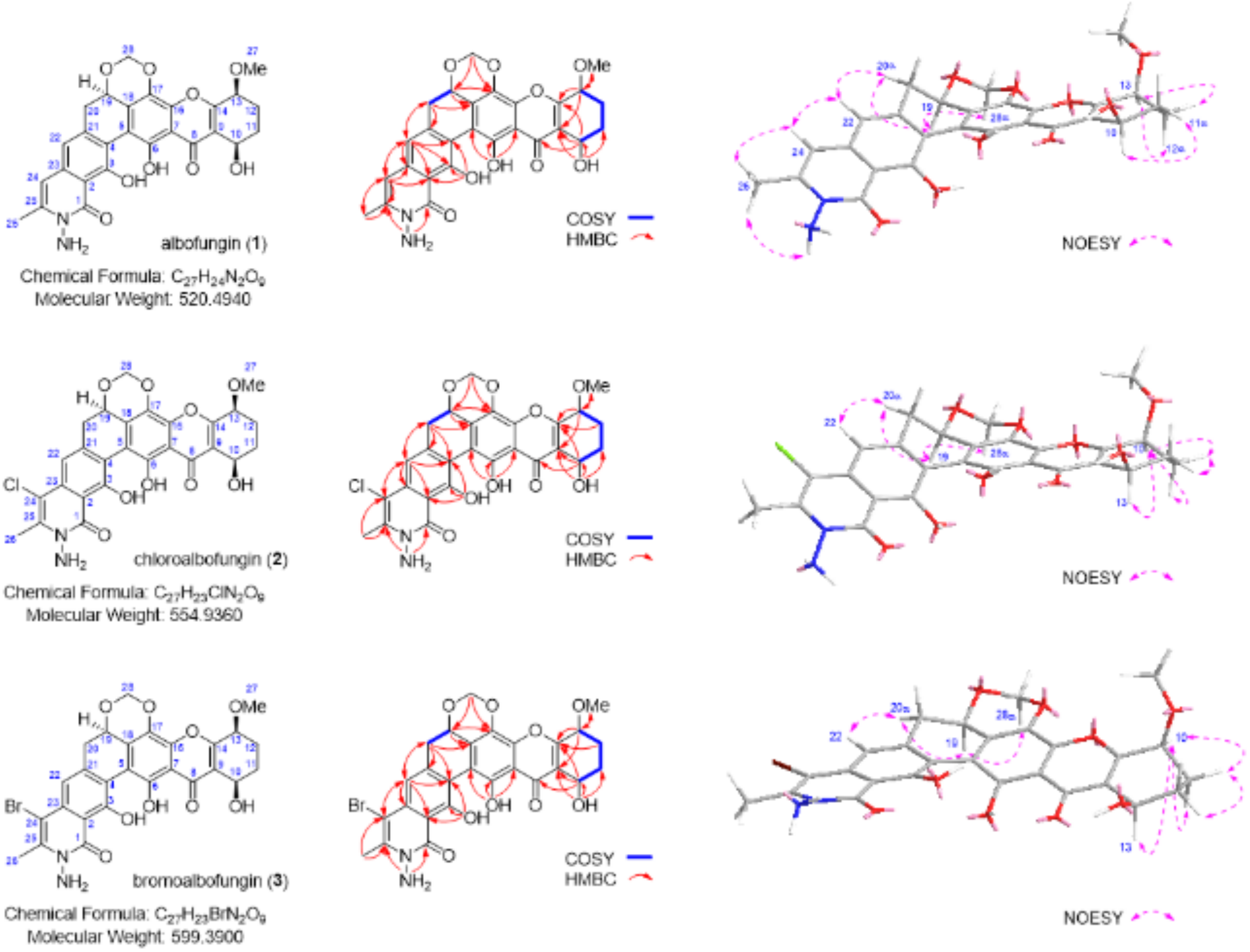

FIG S3. Chemical data of albofungins **1**−**3**. (A) Chemical information of compounds **1**−**3**, including the assignment numbering, structure formulas, and molecular weights. (B) COSY and HMBC correlations of compounds **1**−**3**. COSY correlations, the bold blue lines; HMBC correlations, the red arrows. (C) NOESY correlations of compounds **1**−**2**. NOESY correlations, pink double-head dashed arrows.

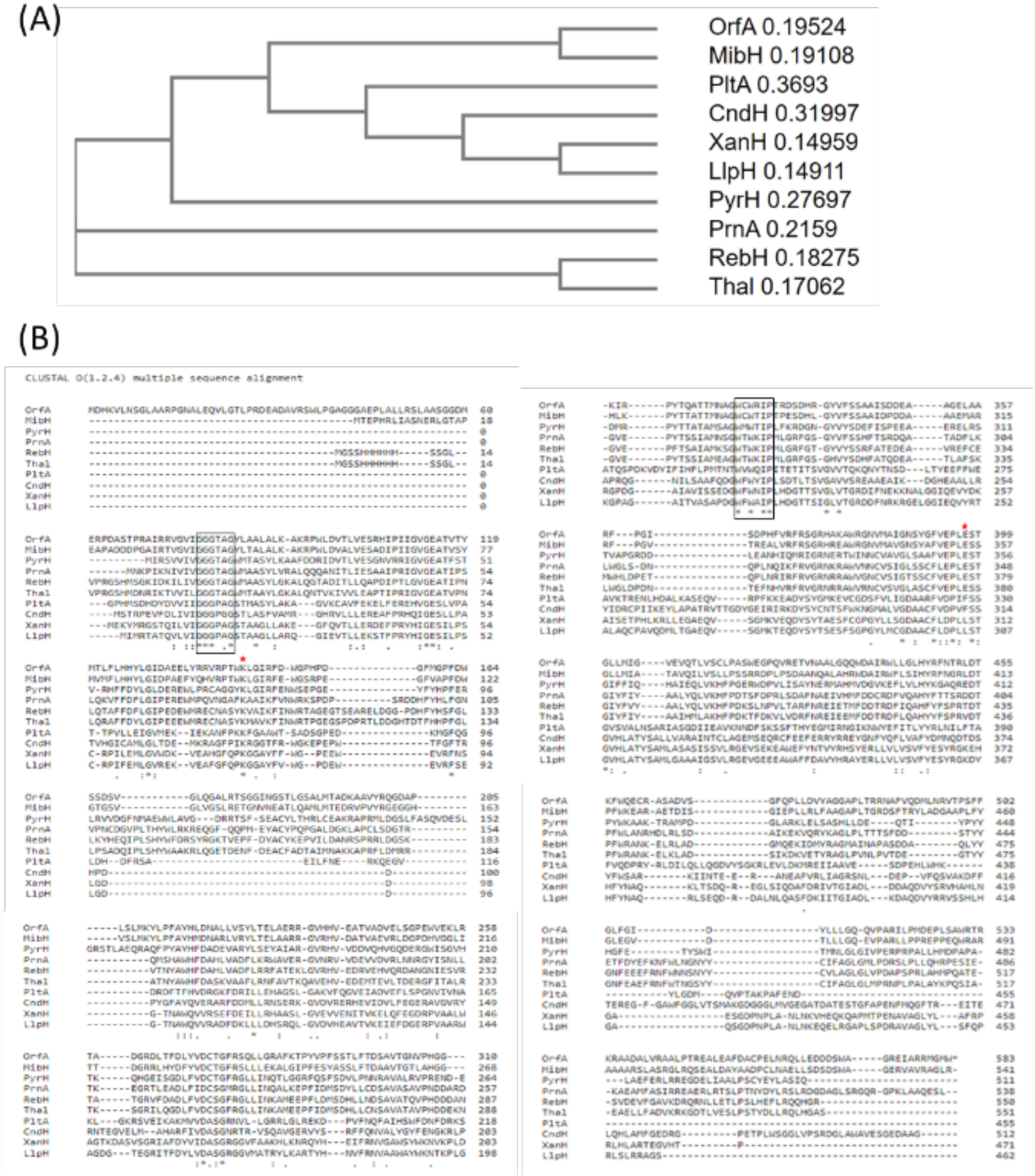

FIG S4. Bioinformatic analysis of OrfA and nine selected FAD-dependent halogenases (FDHs), MibH, PyrH, PrnA, RebH, ThaI, PltA, CndH, XanH, and LlpH. (A) The evolutionary relationship of OrfA with nine selected FDHs shown in phylogenetic tree. (B) Overall multi-sequence alignment of OrfA and nine selected FDHs. The red stars indicate catalytic residues K144 and E397 in OrfA.

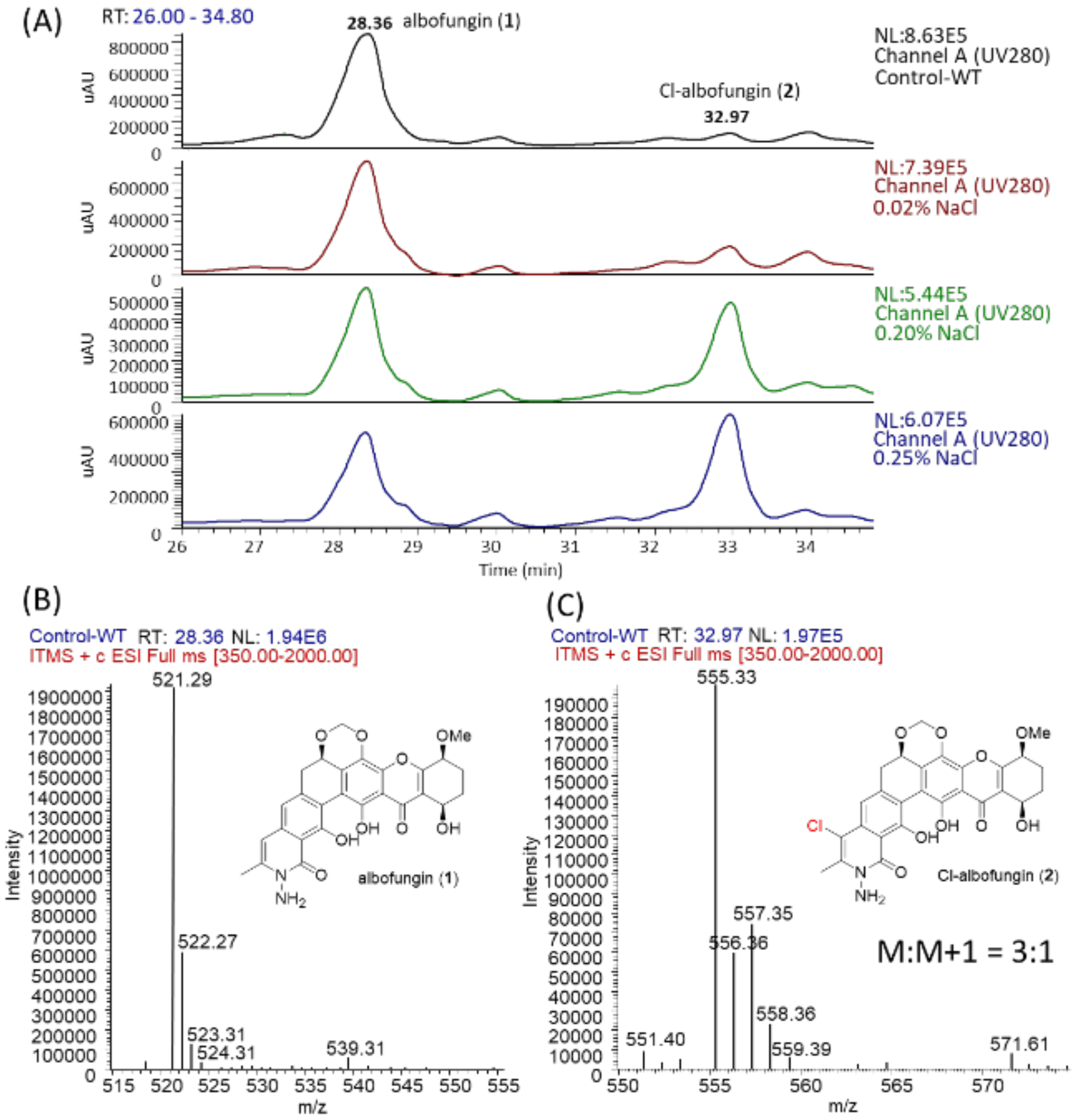

FIG S5. LC-MS spectra of crude extracts from NaCl-feeding fermentations of *S. tumemacerans* JCM5050. (A) LC traces of broth extracts at a given NaCl concentration, 0.02%, 0.20%, or 0.25%. (B) MS of albofungin (**1**). (C) MS of chloroalbofungin (**2**).

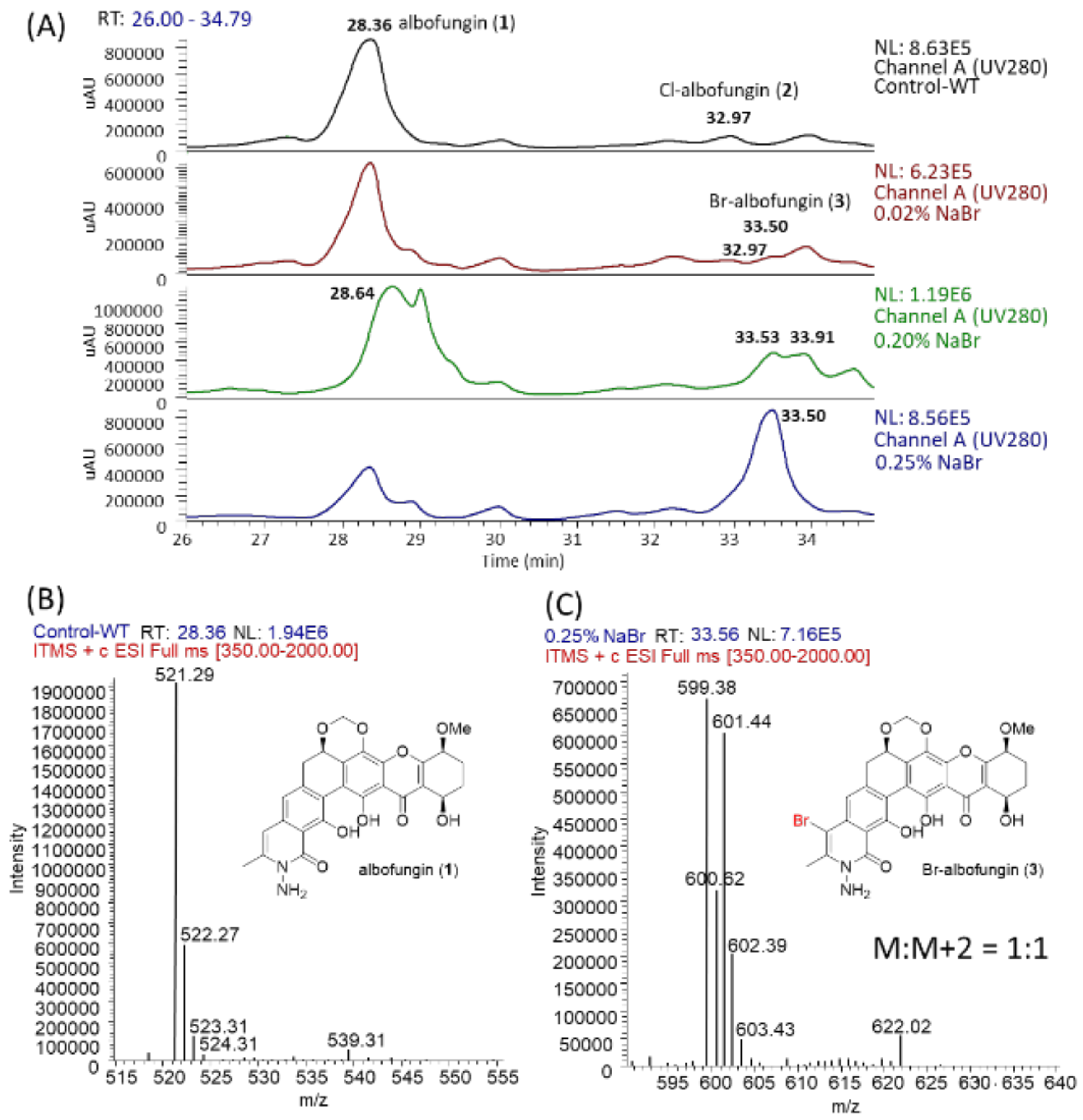

FIG S6. LC-MS spectra of crude extracts from NaBr-feeding fermentations of *S. tumemacerans* JCM5050. (A) LC traces of broth extracts at a given NaBr concentration, 0.02%, 0.20%, or 0.25%. (B) MS of albofungin (**1**). (C) MS of bromoalbofungin (**3**).

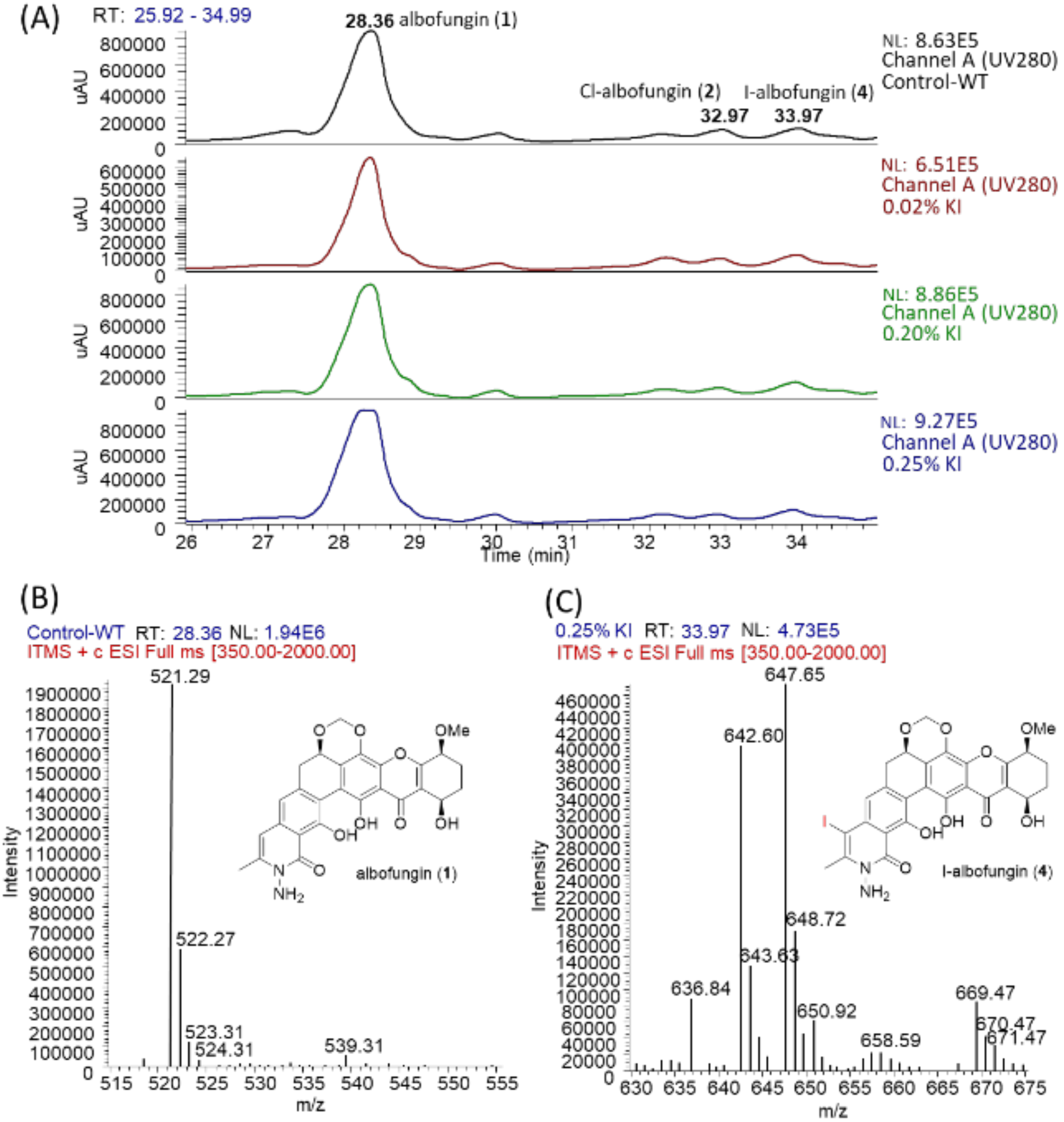

FIG S7. LC-MS spectra of crude extracts from KI-feeding fermentations of *S. tumemacerans* JCM5050. (A) LC traces of broth extracts at a given KI concentration, 0.02%, 0.20%, or 0.25%. (B) MS of albofungin (**1**). (C) MS of iodoalbofungin (**4**). The mass intensity ratio of halide albofungins Cl/I = 10:1 in the 0.25% KI condition, suggesting the iodination is low.

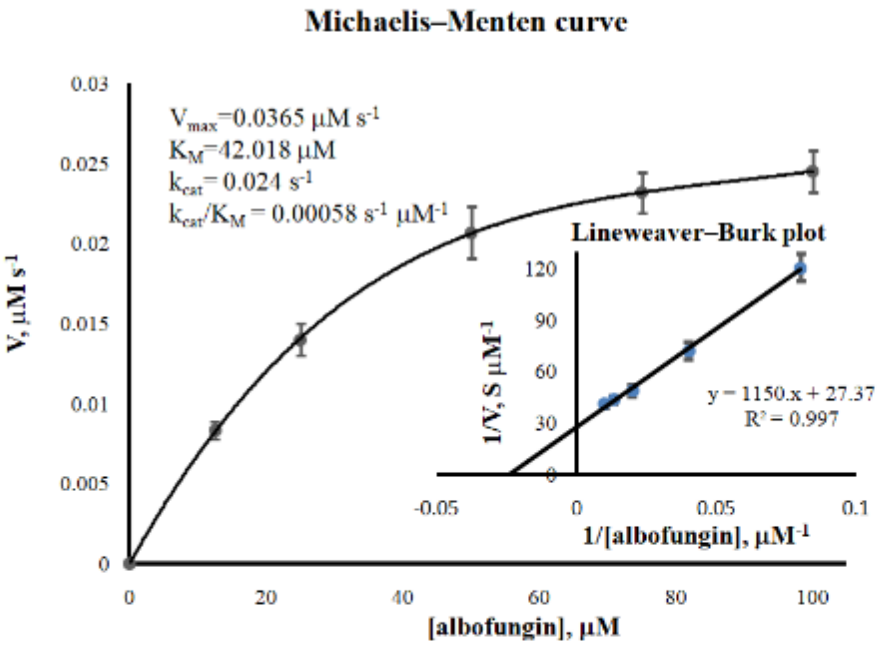

FIG S8. Kinetics of OrfA with varying concentrations of albofungin (**1**).

TC-BLAST RESULTS

heere

**Version:**	blastp
**Reference:**	Altschul, Stephen F., Thomas L. Madden, Alejandro A. Schaffer, Jinghui Zhang, Zheng Zhang, Webb Miller, and David J. Lipman (1997), "Gapped BLAST and PSI-BLAST: a new generation of protein database search programs", *Nucleic Acids Res.* 25:3389-3402.
**Query:**	>OrfL
**Query Length:**	502
**# of TMSs in Query (HMMTOP):**	14
**Perform PSI-BLAST with iterations**	2 PSI-Blast

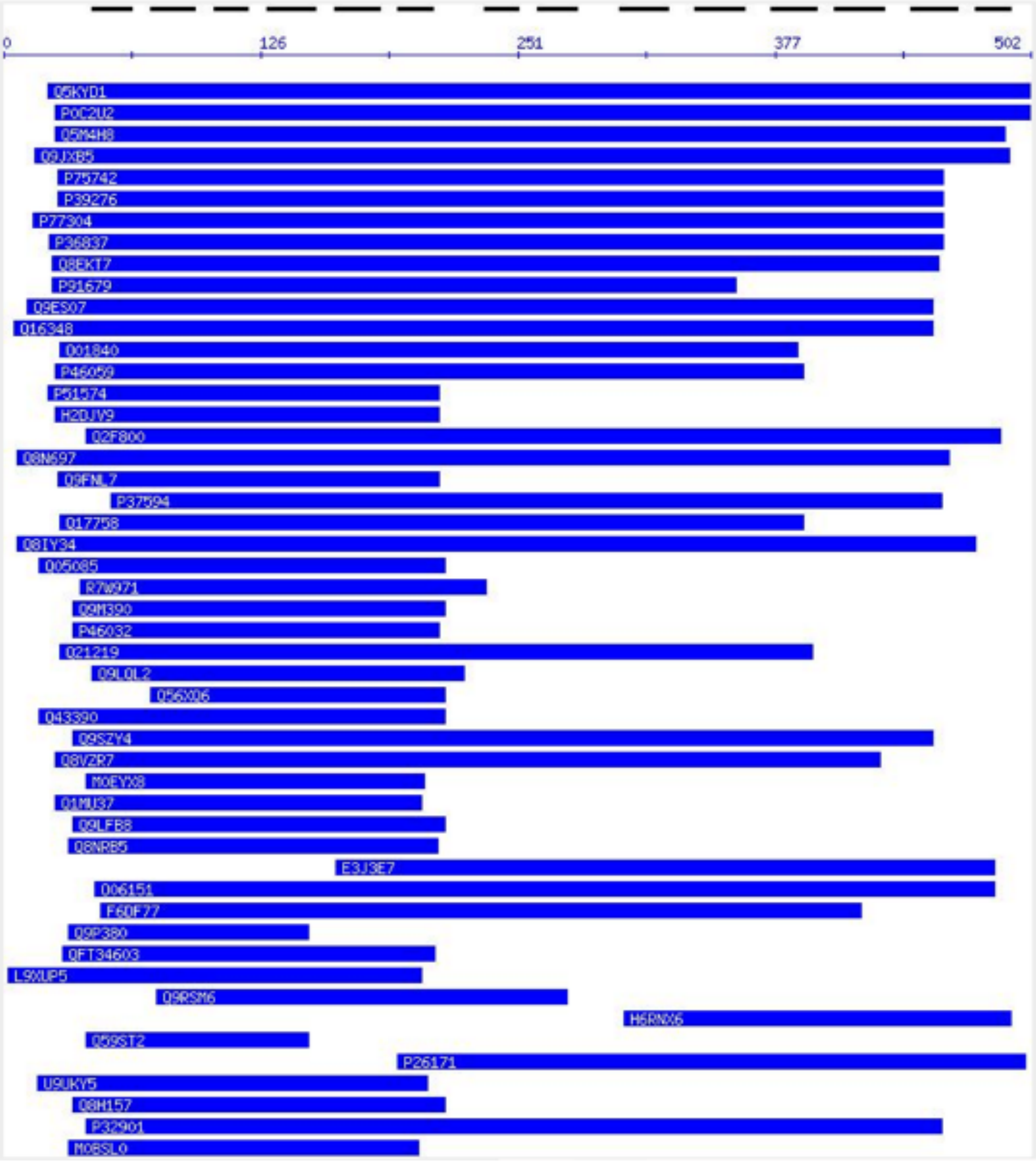

Q5KYD1	14 TMSs	2.A.17.1.7	Di-tripeptide ABC transporter (Per...	862	e-109
P0C2U2	13 TMSs	2.A.17.1.1	Di-/tripeptide transporter - Lacto...	800	e-100
Q5M4H8	14 TMSs	2.A.17.1.6	Di-or tripeptide:H+ symporter OS=S...	741	e-91
Q9JXB5	14 TMSs	2.A.17.1.8	Peptide transporter OS=Neisseria m...	646	e-77
P75742	14 TMSs	2.A.17.1.4	Inner membrane transporter ybgH OS...	491	e-55
P39276	14 TMSs	2.A.17.1.5	Probable dipeptide and tripeptide ...	490	e-55
P77304	14 TMSs	2.A.17.1.2	Tripeptide permease tppB - Escheri...	463	e-51
P36837	14 TMSs	2.A.17.1.3	Inner membrane transporter yhiP - ...	443	e-48
Q8EKT7	14 TMSs	2.A.17.4.7	Proton/peptide symporter family pr...	296	e-29
P91679	10 TMSs	2.A.17.4.2	OLIGOPEPTIDE TRANSPORTER 1 (YIN PR...	233	e-20
Q9ES07	11 TMSs	2.A.17.4.4	Solute carrier family 15 member 2 ...	231	e-20
Q16348	11 TMSs	2.A.17.4.8	Solute carrier family 15 member 2 ...	218	e-18
O01840	12 TMSs	2.A.17.4.10	Peptide transporter 3 OS=Caenorhab...	210	e-17
P46059	11 TMSs	2.A.17.4.9	Solute carrier family 15 member 1 ...	196	e-16
P51574	11 TMSs	2.A.17.4.1	OLIGOPEPTIDE TRANSPORTER, SMALL IN...	181	e-14
H2DJV9	12 TMSs	2.A.17.4.6	Oligopeptide transporter PEPT1 OS=...	180	e-14
Q2F800	12 TMSs	2.A.17.4.5	Peptide transporter PEPT2 - Danio ...	169	e-12
Q8N697	12 TMSs	2.A.17.3.11	Solute carrier family 15 member 4 ...	163	e-12
Q9FNL7	12 TMSs	2.A.17.3.4	Peptide transporter PTR3-A - Arabi...	152	e-10
P37594	14 TMSs	2.A.1.3.14	Methyl viologen resistance protein...	149	e-10
Q17758	11 TMSs	2.A.17.4.3	Peptide transporter family 2	148	e-10
Q8IY34	12 TMSs	2.A.17.3.9	Solute carrier family 15 member 3 ...	143	e-09
Q05085	12 TMSs	2.A.17.3.1	NITRATE/CHLORATE TRANSPORTER - Ara...	143	e-09
R7W971	12 TMSs	2.A.17.3.22	Peptide transporter PTR3-A OS=Aegi...	141	e-09
Q9M390	11 TMSs	2.A.17.3.7	Peptide transport-like protein OS=...	140	e-09
P46032	11 TMSs	2.A.17.3.2	PEPTIDE TRANSPORTER PTR2-B (HISTID...	139	e-09
Q21219	10 TMSs	2.A.17.4.11	Peptide transporter family 1 OS=Ca...	139	e-09
Q9LQL2	12 TMSs	2.A.17.3.19	Nitrate transporter 1.5 OS=Arabido...	138	e-09
Q56XQ6	13 TMSs	2.A.17.3.24	Protein NRT1/ PTR FAMILY 4.4 OS=Ar...	135	e-08
Q43390	12 TMSs	2.A.17.3.3	RCH2 PROTEIN - Brassica napus (Rap...	134	e-08
Q9SZY4	12 TMSs	2.A.17.3.18	Nitrate transporter 1.4 OS=Arabido...	131	e-08
Q8VZR7	12 TMSs	2.A.17.3.23	Protein NRT1/ PTR FAMILY 5.1 OS=Ar...	131	e-08
M0EYX8	12 TMSs	2.A.1.55.1	Major facilitator superfamily MFS_...	128	e-08
Q1MU37	12 TMSs	2.A.17.2.4	Putative peptide transporter OS=Tr...	120	e-07
Q9LFB8	11 TMSs	2.A.17.3.8	Peptide transporter PTR5	118	e-06
Q8NRB5	12 TMSs	2.A.1.2.24	Permeases of the major facilitator...	114	e-06
E3J3E7	12 TMSs	2.A.1.29.3	Major facilitator superfamily MFS_...	112	e-06
O06151	14 TMSs	2.A.1.3.48	Probable multidrug-efflux transpor...	110	e-05
F6DF77	12 TMSs	2.A.1.51.3	Major facilitator superfamily MFS_...	106	e-05
Q9P380	12 TMSs	2.A.17.2.1	Probable peptide transporter ptr2	107	e-05
QFT34603	11 TMSs	2.A.1.55.5	Major Facilitator Superfamily prot...	106	e-05
L9XUP5	12 TMSs	2.A.1.55.2	Major facilitator superfamily prot...	106	e-05
Q9RSM6	14 TMSs	2.A.1.3.67	Uncharacterized protein OS=Deinoco...	106	e-05
H6RNX6	10 TMSs	2.A.7.3.48	Multidrug resistance efflux transp...	104	e-05
Q59ST2	12 TMSs	2.A.17.2.5	Ptr22p OS=Candida albicans (strain...	102	0.0001
P26171	12 TMSs	2.A.1.41.3	BACTERIOCHLOROPHYLL SYNTHASE 44.5 ...	99	0.0003
U9UKY5	12 TMSs	2.A.17.2.3	Uncharacterized protein OS=Rhizoph...	98	0.0004
Q8H157	12 TMSs	2.A.17.3.16	Nitrate transporter 1.2 OS=Arabido...	98	0.0004
P32901	12 TMSs	2.A.17.2.2	PEPTIDE TRANSPORTER PTR2 (PEPTIDE ...	98	0.0004
M0BSL0	12 TMSs	2.A.1.55.3	Major facilitator superfamily prot...	96	0.0006

FIG S9. The TCDB-BLAST result of OrfL. The BLAST result indicates that OrfL is a homologous enzyme of the nitrate/chloride transporter family.

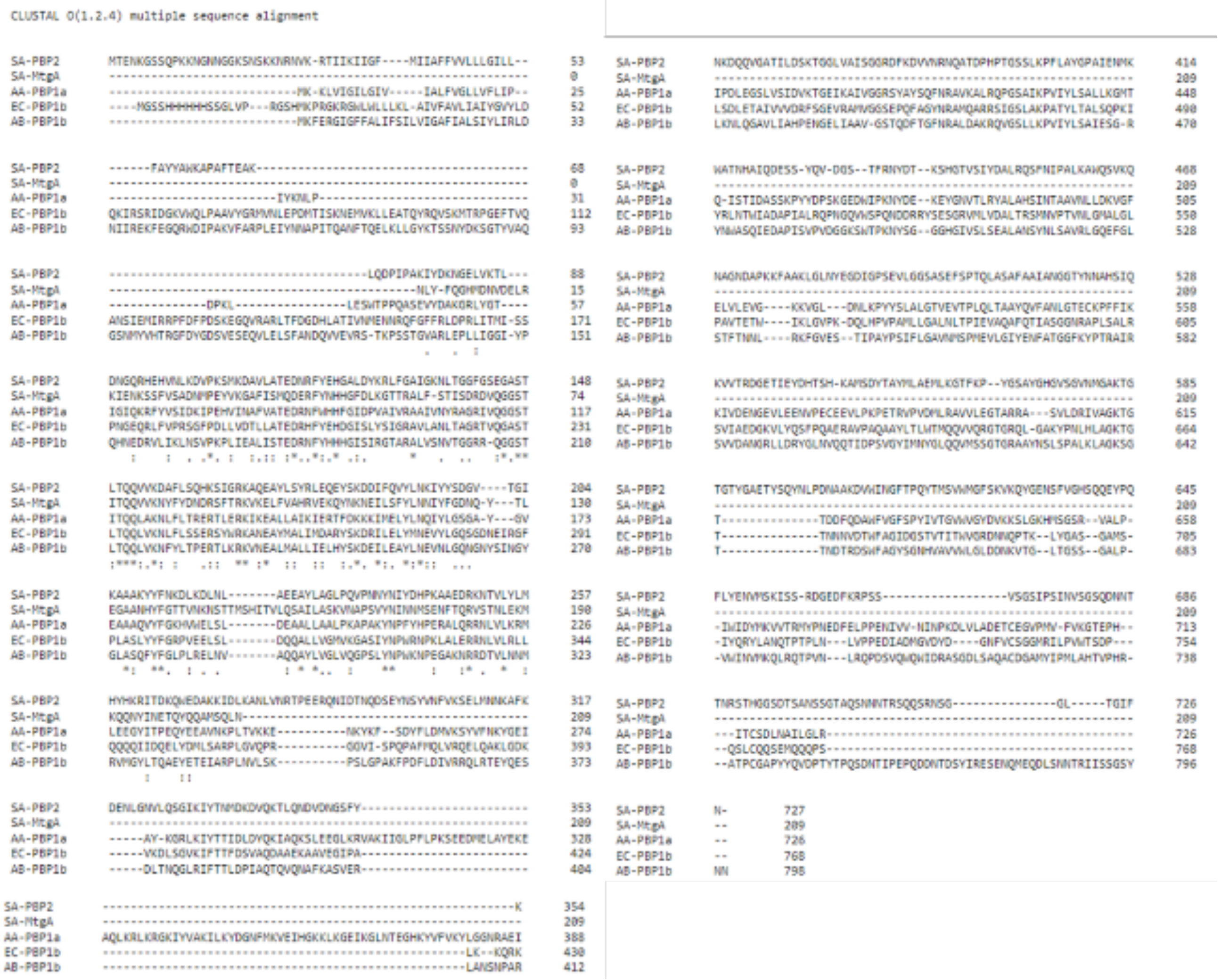

FIG S10. The multi-sequence alignment of the TGase-domains from different TGases, including *S. aureus* MtgA, *S. aureus* PBP2, *Aquifex aeolicus* PBP1a, and *E. coli* PBP1b.

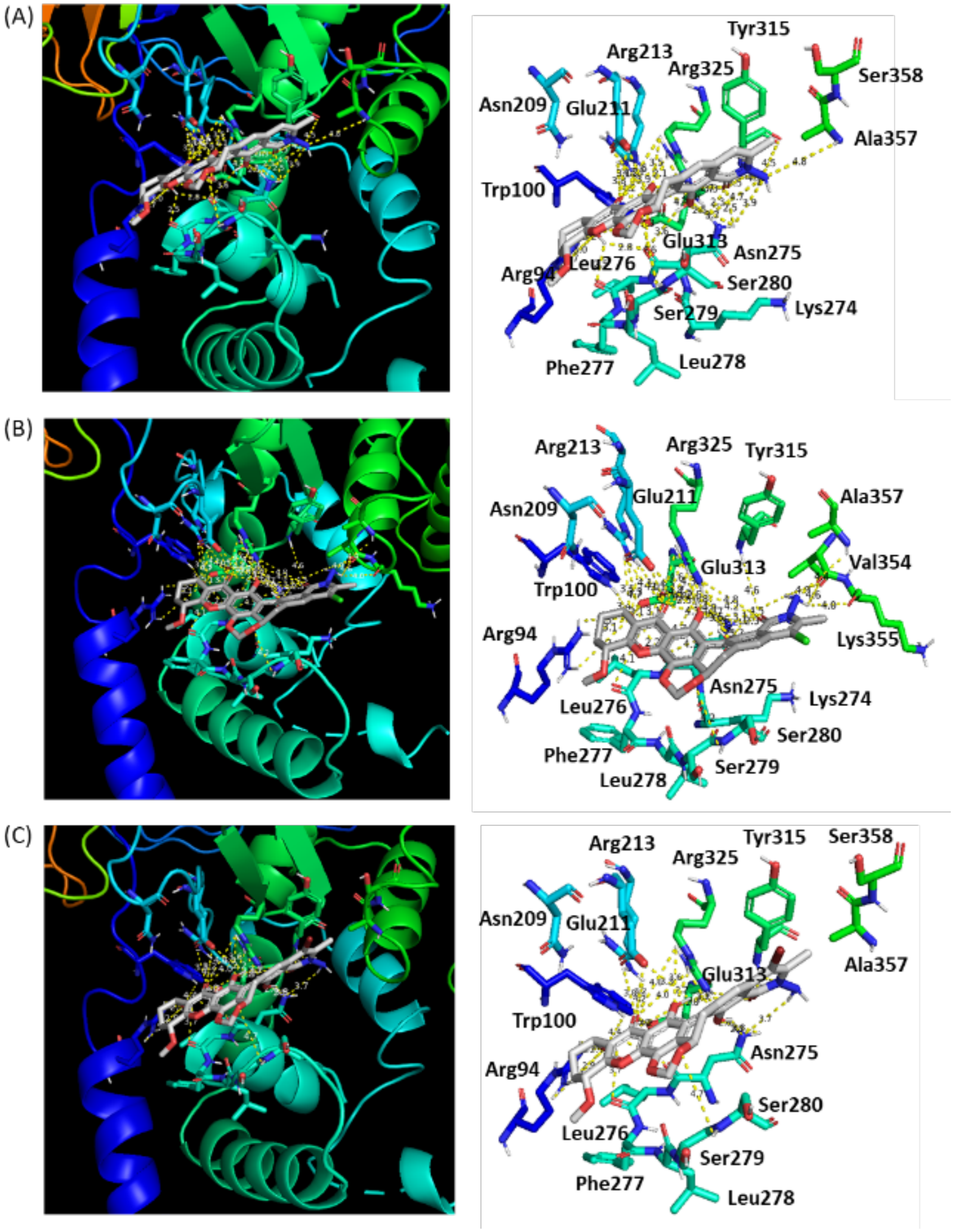

FIG S11. AutoDock simulation of PBP1b and albofungins **1**−**3**. (A)The H-bonds between PBP1b and albofungin (**1**) are observed from the NH_2 and $C{=}NH_2^+$ on the guanidine of **Arg94** to 10-OH within 2.2 (3.9) and 3.0 (4.7) Å; the NH on indole of **Trp100** to 10-OH within 4.4 Å; the δ-C=O of **Glu211**

to the 6-OH within 4.1 Å; the δ-OH of **Glu211** to the 6-OH and O atom of C-8 ketone within 3.1 and 3.1 Å; the C=NH_2^+ on the guanidine of **Arg213** to the O atoms of C-8 ketone within 3.8 (4.2) Å; the C=O of **Asn275** to the 3-OH, 6-OH, and 10-OH within 3.6, 3.3, and 2.8 Å; the γ-NH_2 of **Asn275** to the O atom of C-1 ketone, 3-OH, 6-OH, and N-NH_2 within 2.3 (2.5), 2.5 (3.7), 4.4, and 3.9 (4.7) Å; the C=O of **Leu276** to 10-OH within 2.5 Å; the α-NH of **Ser280** to the O atom of (C-19)-O-(C-28) ether within 4.6 Å; the δ-C=O of **Glu313** to 6-OH within 3.7 Å; the δ-OH of **Glu313** to 10-OH within 4.5 Å; the C=O of **Tyr315** to the N-NH_2 within 4.5 Å; the α-NH_2 of **Tyr315** to the N-NH_2 and C-1 ketone within 4.3 and 3.5 Å; the NH_2 on the guanidine of **Arg325** to the O atom of C-1 ketone, 3-OH, 6-OH, and C-8 ketone within 2.7 (3,3), 2.0 (3.6), 2.7 (4.2), and 4.5 Å; the C=NH_2^+ on the guanidine of **Arg325** to the O atoms of C-1 ketone, 3-OH, 6-OH, and C-8 ketone within 4.2, 3.2, 2.1 (3.5), and 3.9 (4.5) Å, respectively; the α-NH_2 of **Ala357** to the N-NH_2 within 4.8 Å. (B) The H-bonds between PBP1b and chloroalbofungin (**2**) are observed from the NH_2 and C=NH_2^+ on the guanidine of **Arg94** to 10-OH within 3.1 (4.3) and 4.7 Å; the NH on indole of **Trp100** to 10-OH within 3.4 Å; the δ-C=O of **Glu211** to the 10-OH within 4.7 Å; the δ-OH of **Glu211** to the 6-OH and O atom of C-8 ketone within 4.4 and 3.1 Å; the C=NH_2^+ on the guanidine of **Arg213** to the O atoms of C-8 ketone and 10-OH within 4.0 (4.1) and 3.3 (3.35) Å; the C=O of **Asn275** to the 3-OH, 6-OH, and 10-OH within 4.3, 4.7, and 2.2 Å; the γ-NH_2 of **Asn275** to the O atom of C-1 ketone, 3-OH, 6-OH, C-8 ketone, and N-NH_2 within 2.3 (3.1), 2.8 (3.2), 3.2 (4.6), 4.3, and 4.5 Å; the C=O of **Leu276** to 10-OH within 4.1 Å; the α-NH of **Ser280** to the O atom of (C-19)-O-(C-28) ether within 4.2 Å; the δ-C=O of **Glu313** to 6-OH and 10-OH within 4.8 and 3.1 Å; the δ-OH of **Glu313** to 10-OH within 3.1 Å; the α-NH_2 of **Tyr315** to the O atom of C-1 ketone within 4.6 Å; the NH_2 on the guanidine of **Arg325** to the O atom of C-1 ketone, 3-OH, 6-OH, and C-8 ketone within 4.2 (4.8), 3.5 (4.7), 2.1 (3.8), and 2.8 (3.7) Å; the C=NH_2^+ on the guanidine of **Arg325** to the O atoms of 3-OH, 6-OH, and C-8 ketone within 4.9, 2.5 (4.3), and 2.7 (4.5) Å, the δ-NH of **Arg325** to the O atom of C-8 ketone within 4.6 Å; the C=O of **Val354** to N-NH_2 within 4.6 (4.9) Å; the C=O of **Lys355** to N-NH_2 within 4.0 Å; the α-NH_2 of **Ala357** to the N-NH_2 within 4.6 Å. (C) The H-bonds between PBP1b and chloroalbofungin (**2**) are observed from the NH_2 and C=NH_2^+ on the guanidine of **Arg94** to 10-OH within 2.1 (3.7) and 2.9 (4.6) Å; the NH on indole of **Trp100** to 10-OH within 4.4 Å; the δ-C=O of **Glu211** to the 10-OH within 4.4 Å; the δ-OH of **Glu211** to the 6-OH and O atom of C-8 ketone within 3.2 and 3.3 Å; the C=NH_2^+ on the guanidine of **Arg213** to the O atoms of C-8 ketone within 3.8 (4.2) Å; the C=O of **Asn275** to the 6-OH and 10-OH within 4.6 and 3.4 Å; the γ-NH_2 of **Asn275** to the O atom of C-1 ketone, 3-OH, 6-OH, and N-NH_2 within 2.3 (2.6), 2.6 (3.8), 4.4, and 2.9 (4.3) Å; the C=O of **Leu276** to 10-OH within 3.7 Å; the α-NH of **Ser280** to the O atom of (C-19)-O-(C-28) ether within 4.7 Å; the δ-C=O of **Glu313** to 6-OH within 4.9 Å; the δ-OH of **Glu313** to 10-OH within 4.2 Å; the C=O of **Tyr315** to the N-NH_2 within 4.4 Å; the α-NH_2 of **Tyr315** to the N-NH_2 and C-1 ketone within 4.4 and 3.7 Å; the NH_2 on the guanidine of **Arg325** to the O atom of C-1 ketone, 3-OH, 6-OH, and C-8 ketone within 2.6 (3,4), 2.1 (3.7), 2.8 (4.3), and 4.6 Å; the C=NH_2^+ on the guanidine of **Arg325** to the O atoms of C-1 ketone, 3-OH, 6-OH, and C-8 ketone within 4.1, 3.2, 2.2 (3.6), and 4.0 (4.6) Å, respectively.

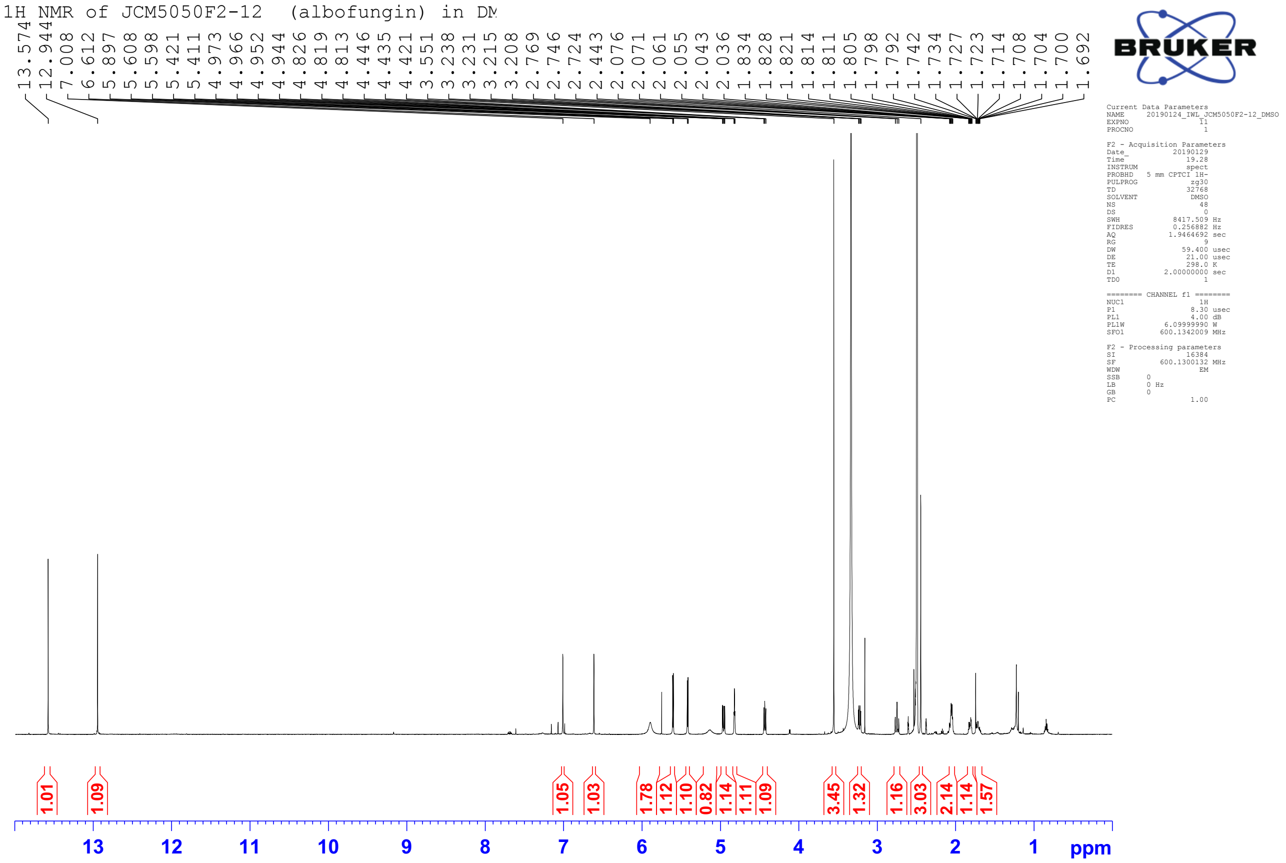

FIG S12. ^{1}H NMR spectrum (600 MHz) of albofungin (**1**) in DMSO-d_6.

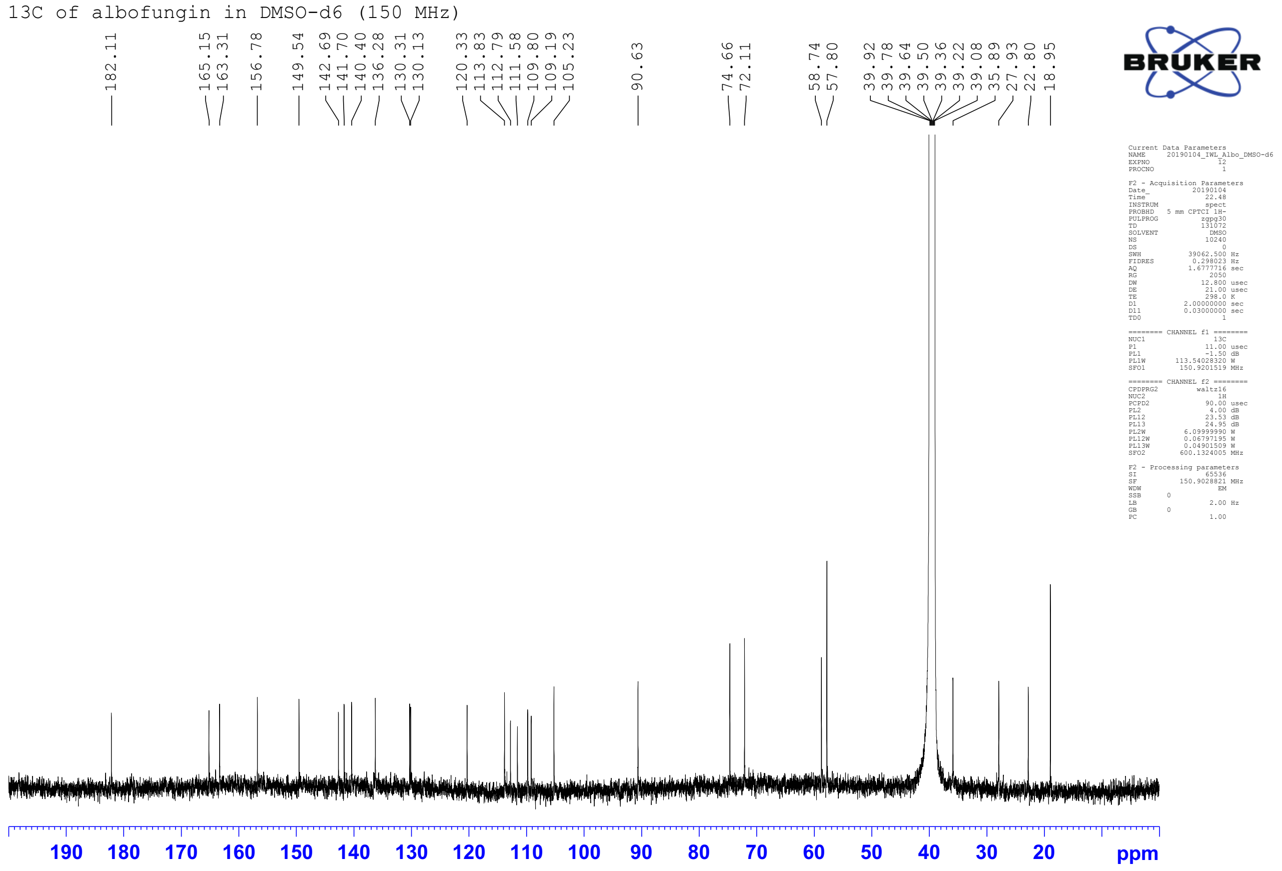

FIG S13. ^{13}C NMR spectrum (150 MHz) of albofungin (**1**) in DMSO-d_6.

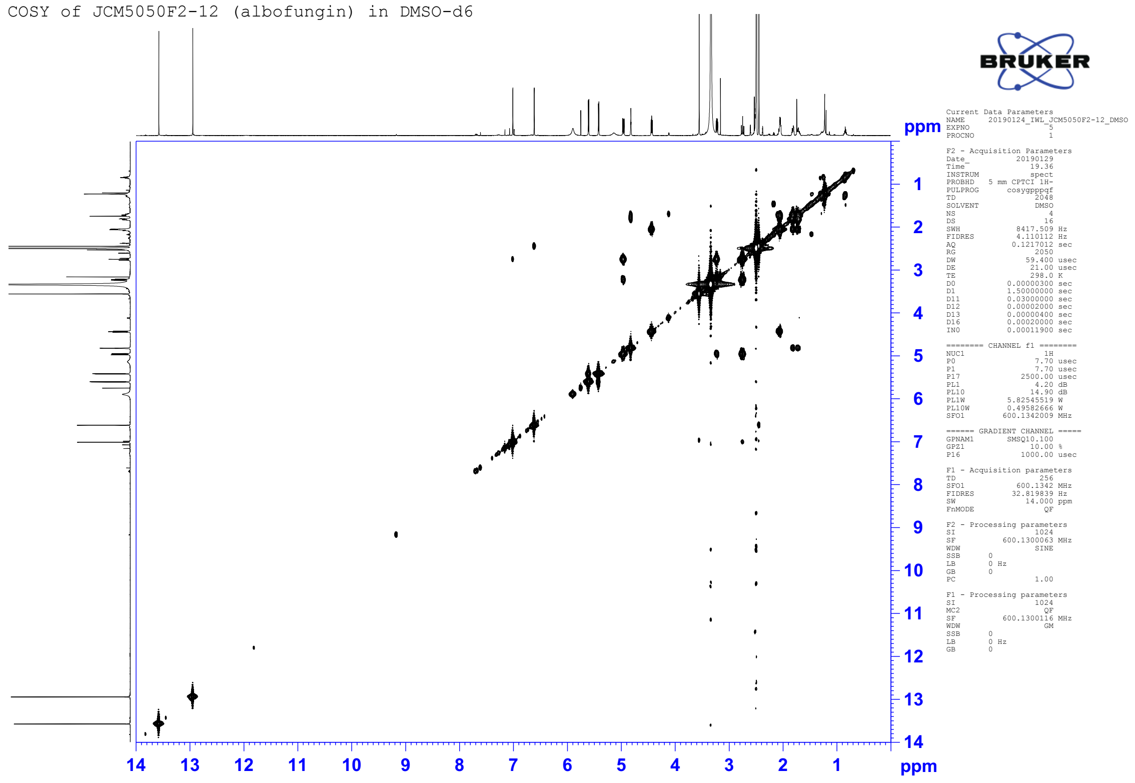

FIG S14. ^{1}H-^{1}H COSY spectrum of albofungin (**1**) in DMSO-d_6.

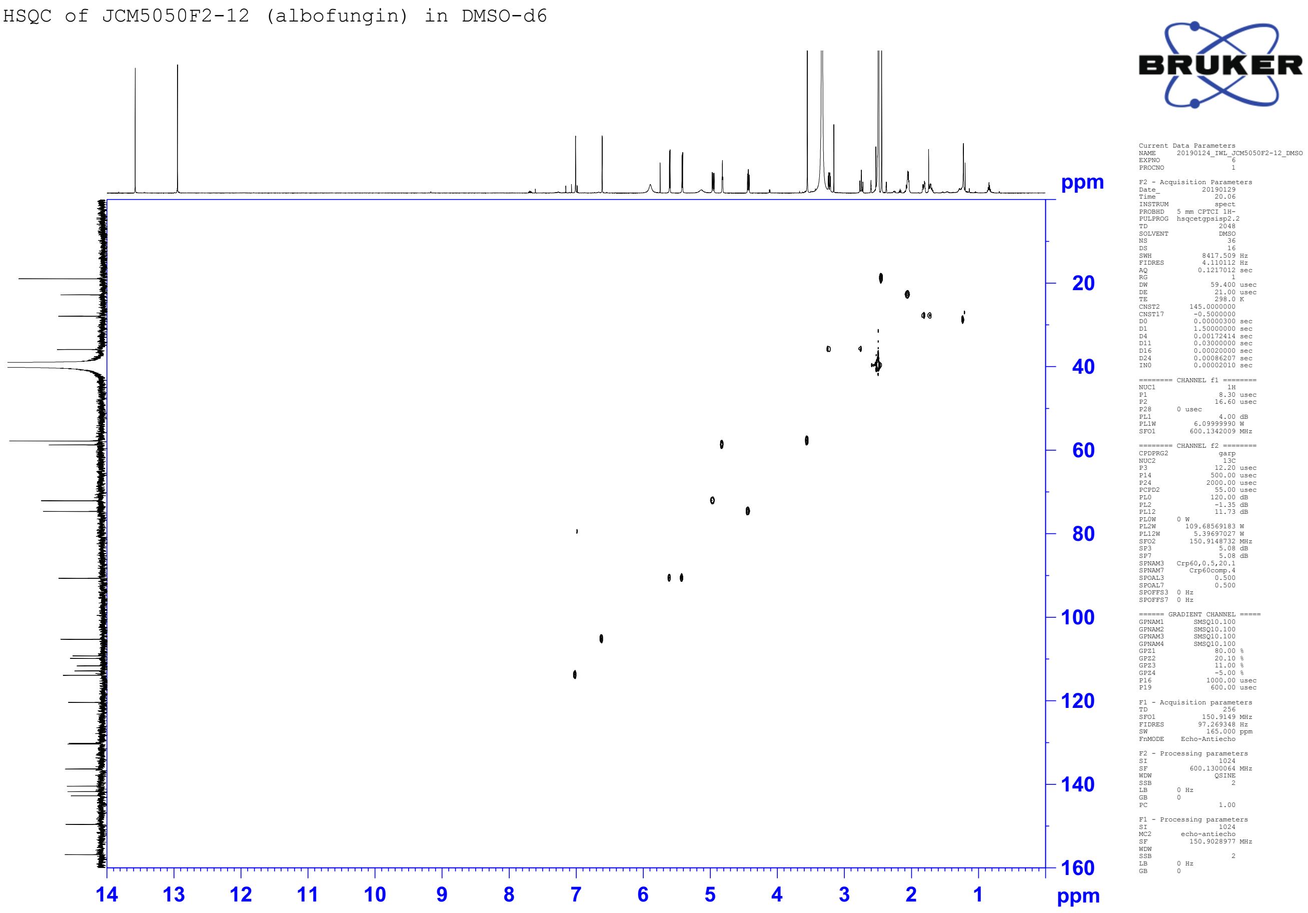

FIG S15. HSQC spectrum of albofungin (**1**) in DMSO-d_6.

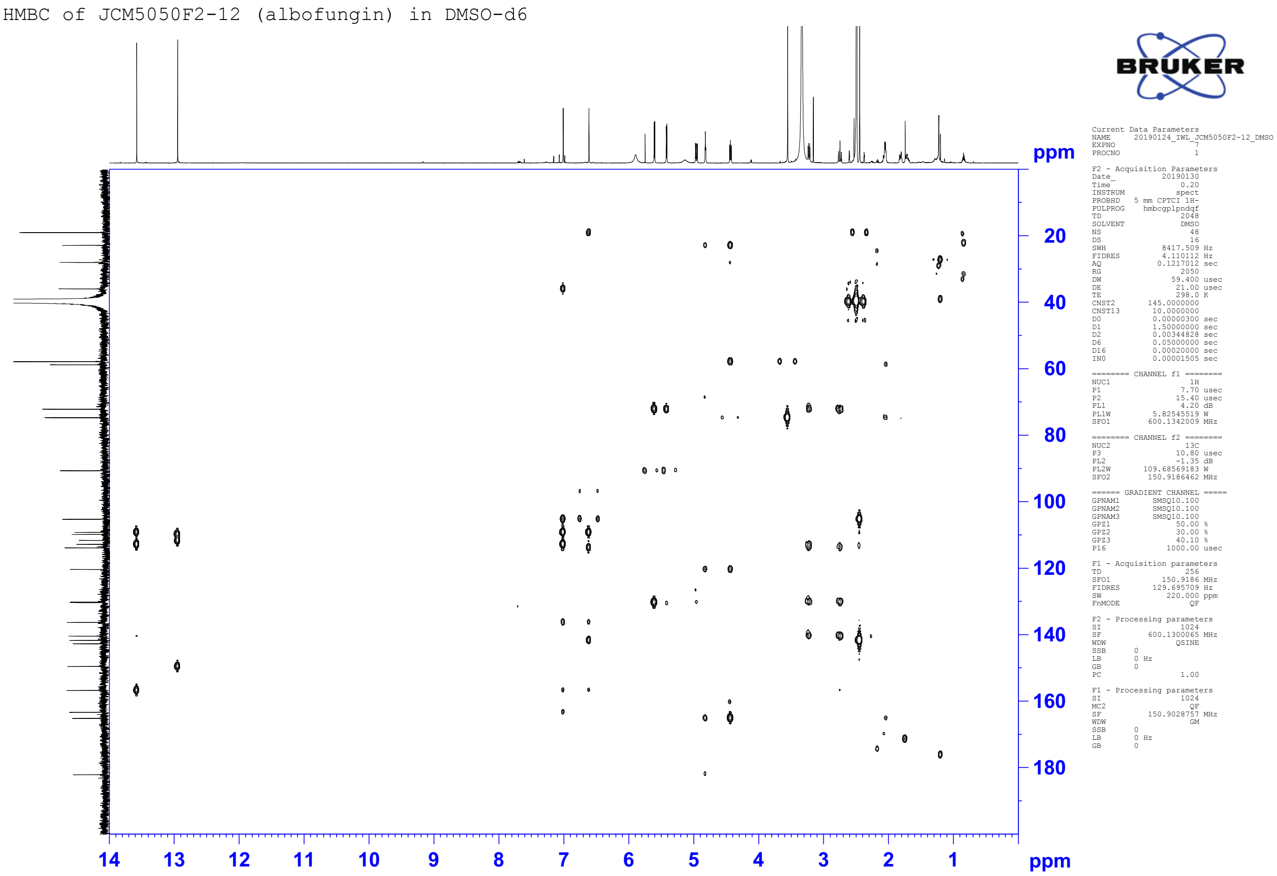

FIG S16. HMBC spectrum of albofungin (**1**) in DMSO-d_6.

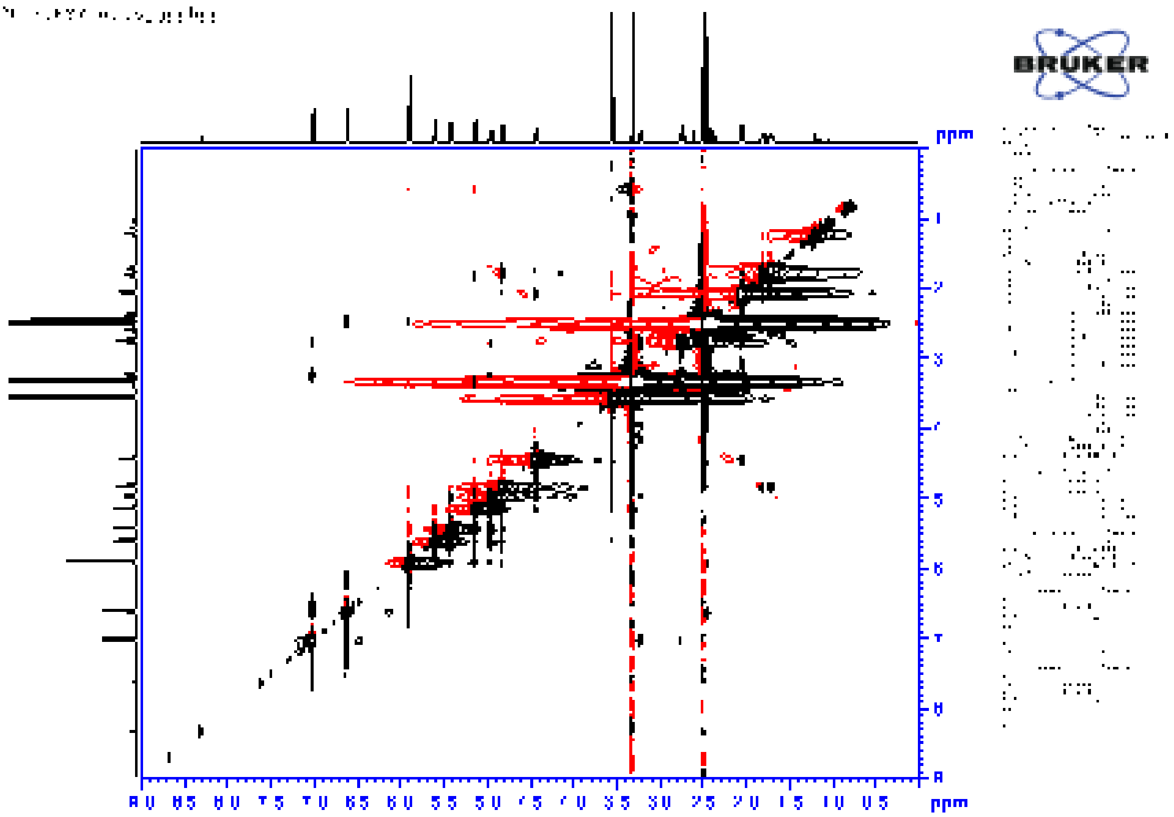

FIG S17. NOESY spectrum of albofungin (**1**) in DMSO-d_6.

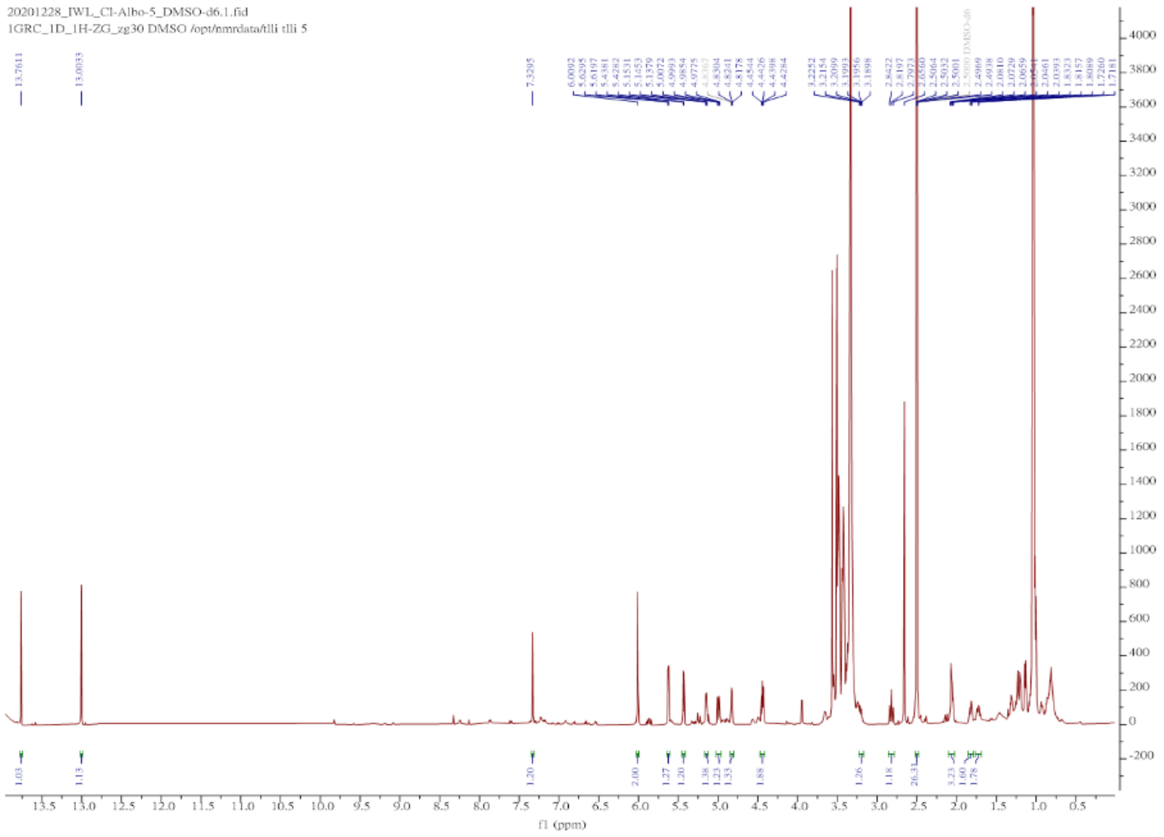

FIG S18. ^{1}H NMR spectrum (600 MHz) of chloroalbofungin (**2**) in DMSO-d_6.

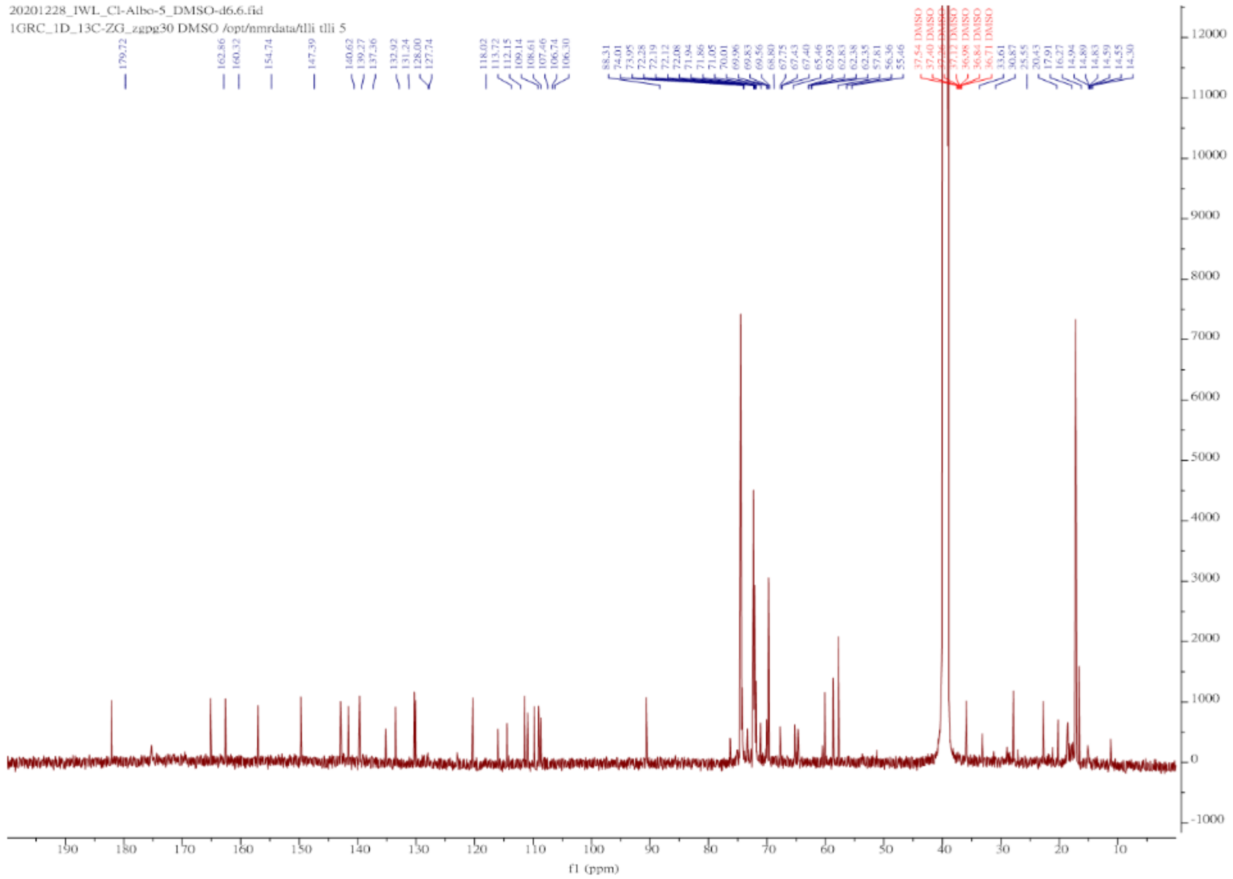

FIG S19. ^{13}C NMR spectrum (150 MHz) of chloroalbofungin (**2**) in DMSO-d_6.

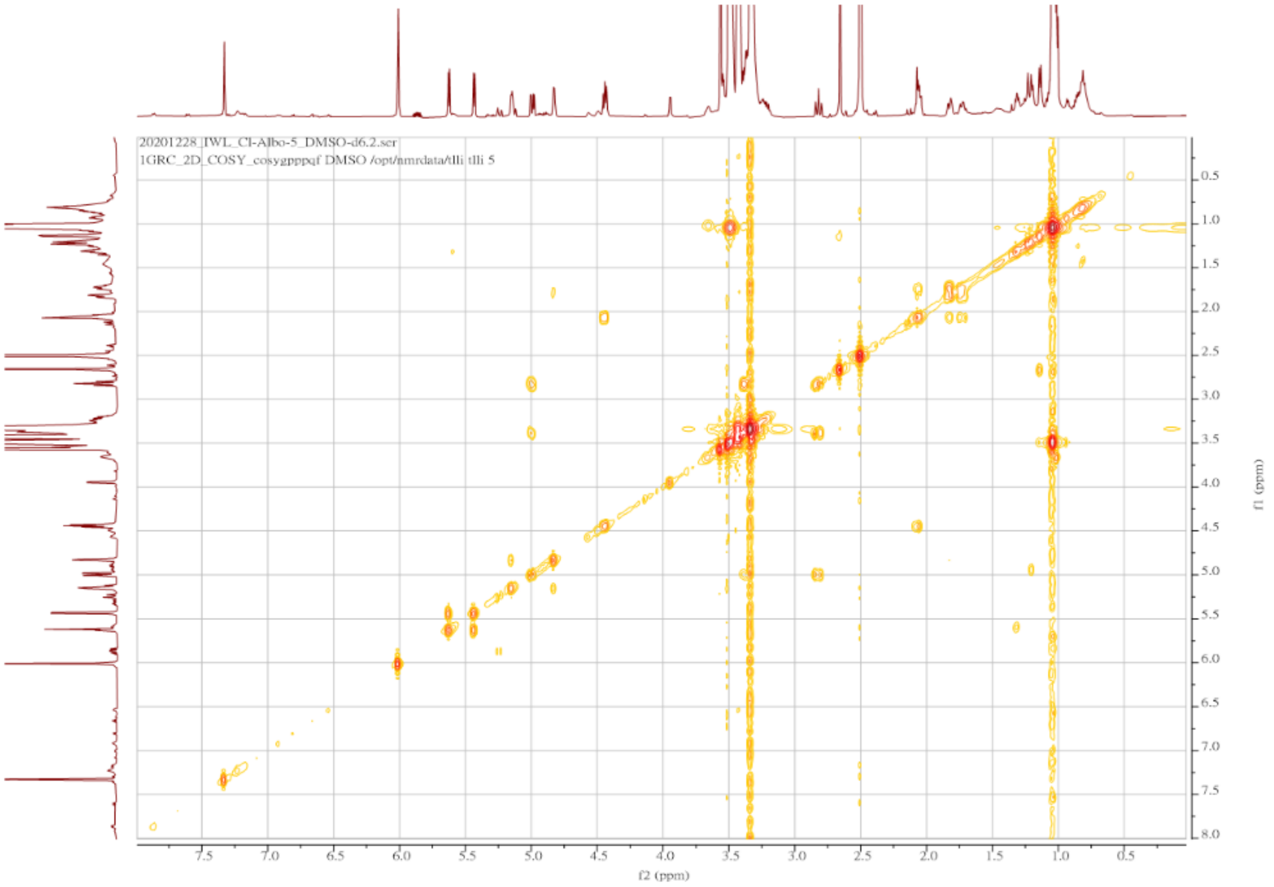

FIG S20. ^{1}H-^{1}H COSY spectrum of chloroalbofungin (**2**) in DMSO-d_6.

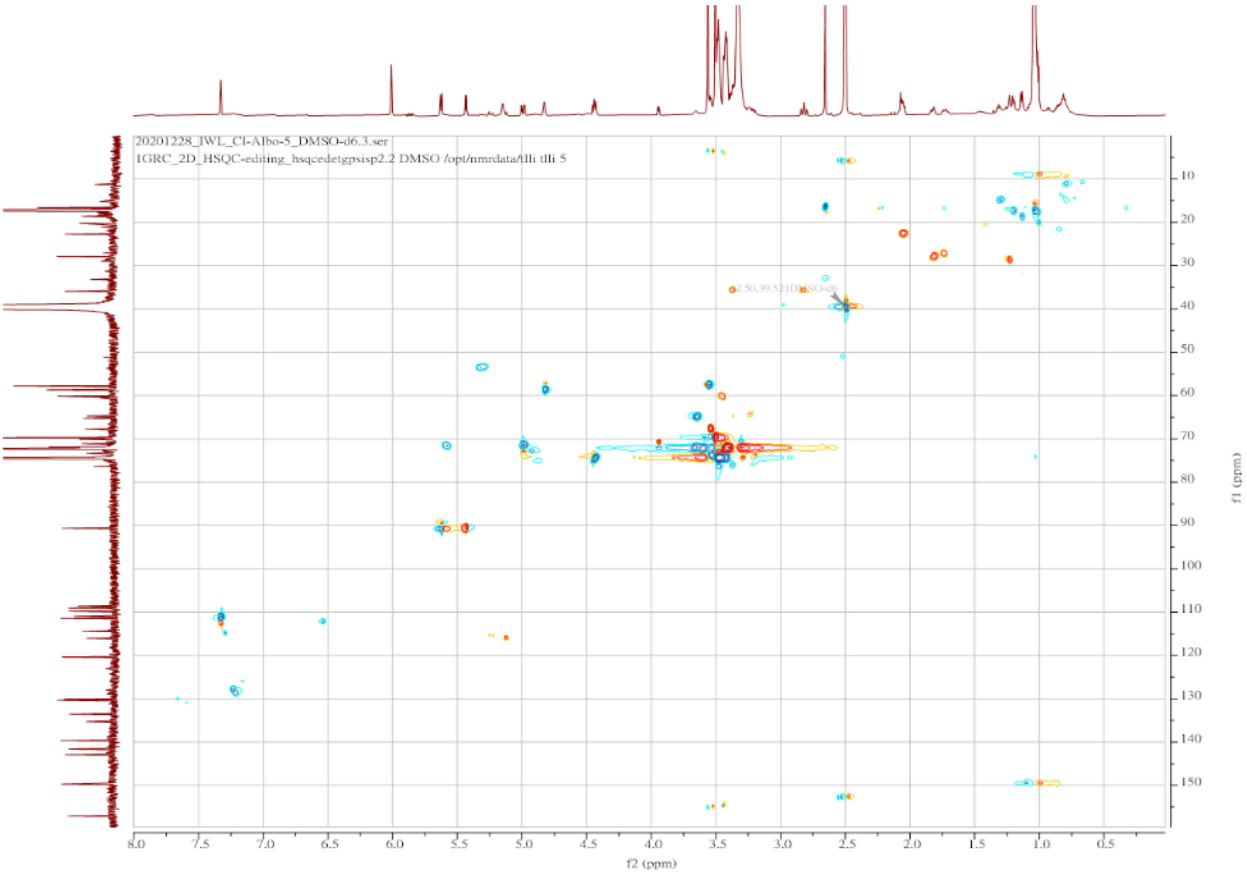

FIG S21. HSQC spectrum of chloroalbofungin (**2**) in DMSO-d_6.

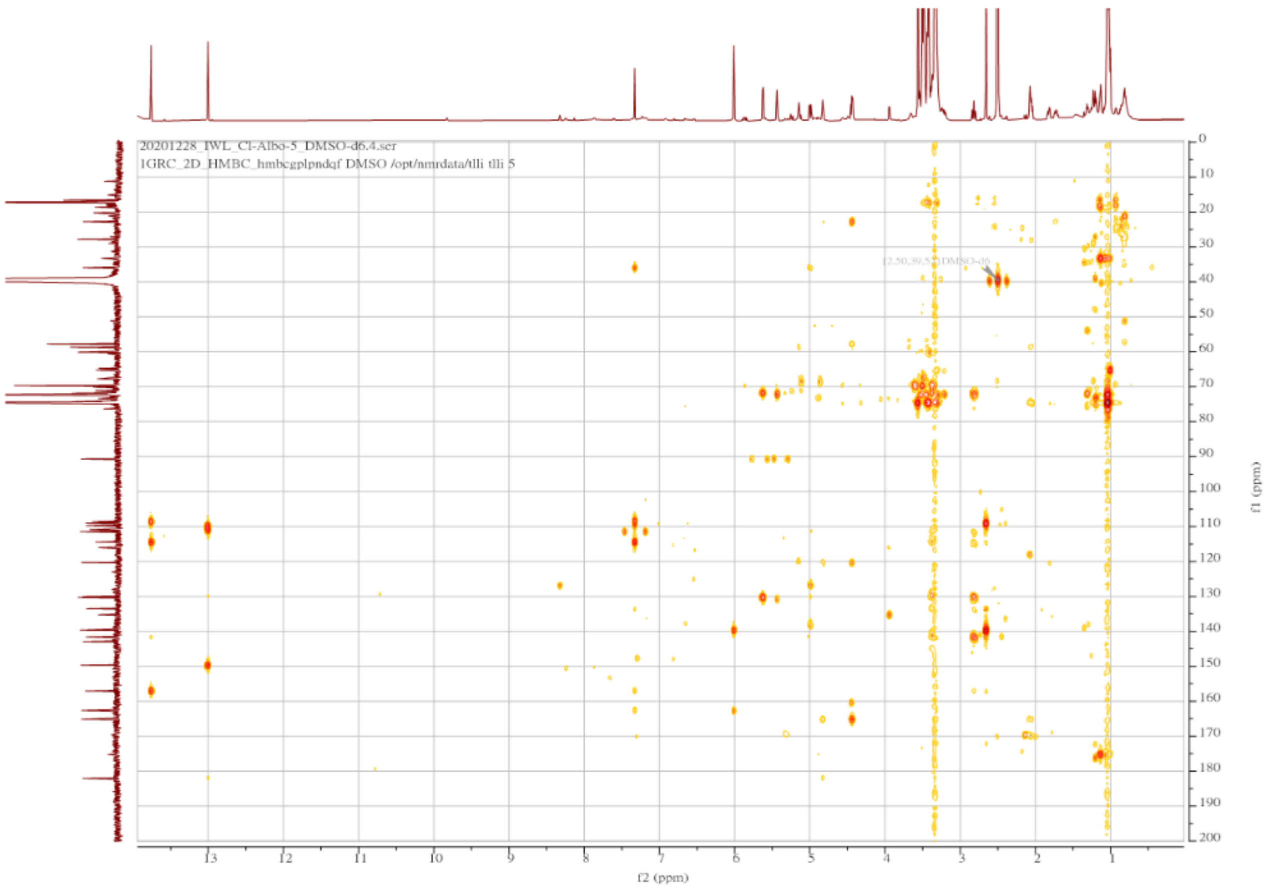

FIG S22. HMBC spectrum of chloroalbofungin (**2**) in DMSO-d_6.

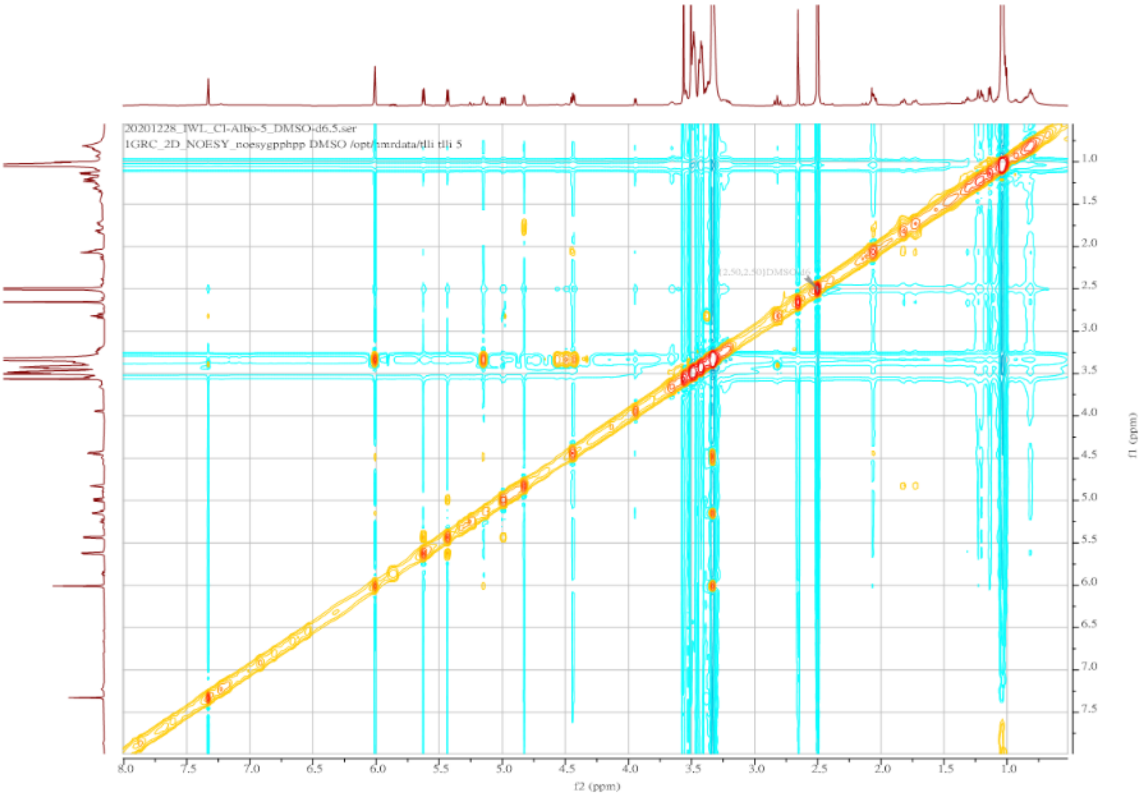

FIG S23. NOESY spectrum of chloroalbofungin (**2**) in DMSO-d_6.

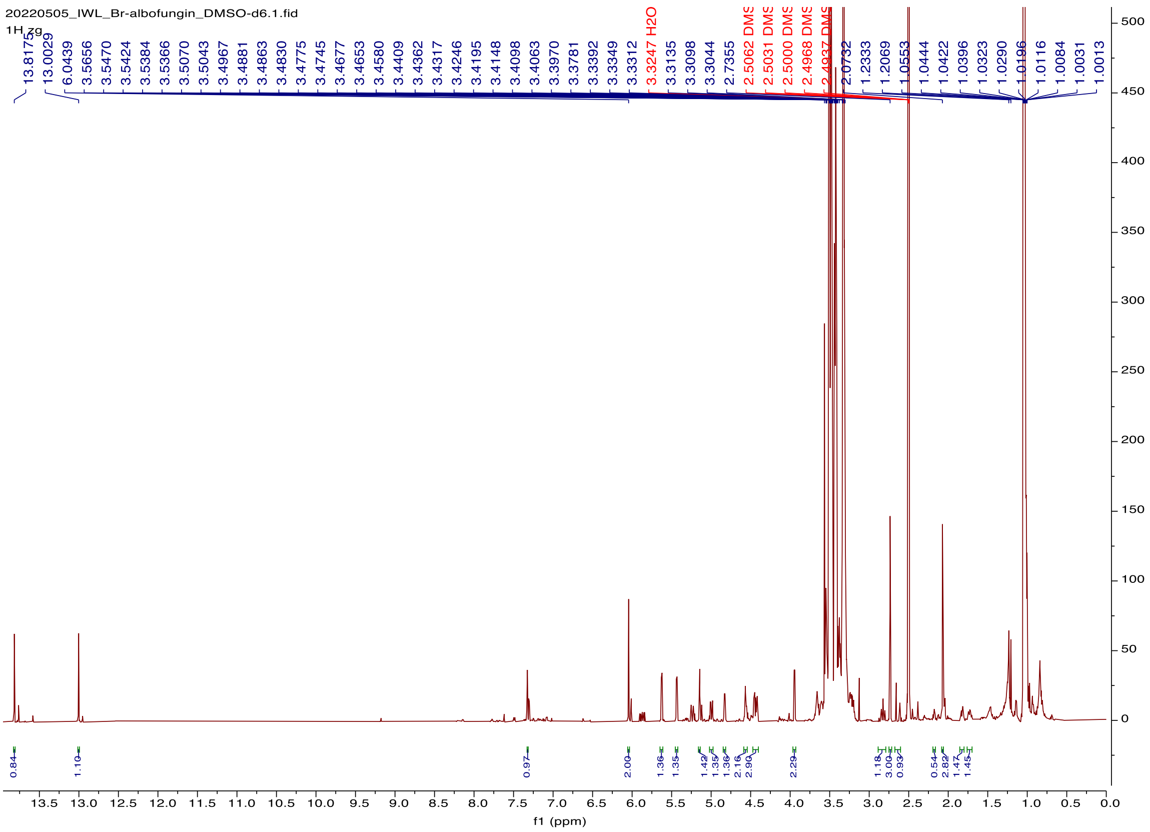

FIG S24. ^{1}H NMR spectrum (600 MHz) of bromoalbofungin (**3**) in DMSO-d_6.

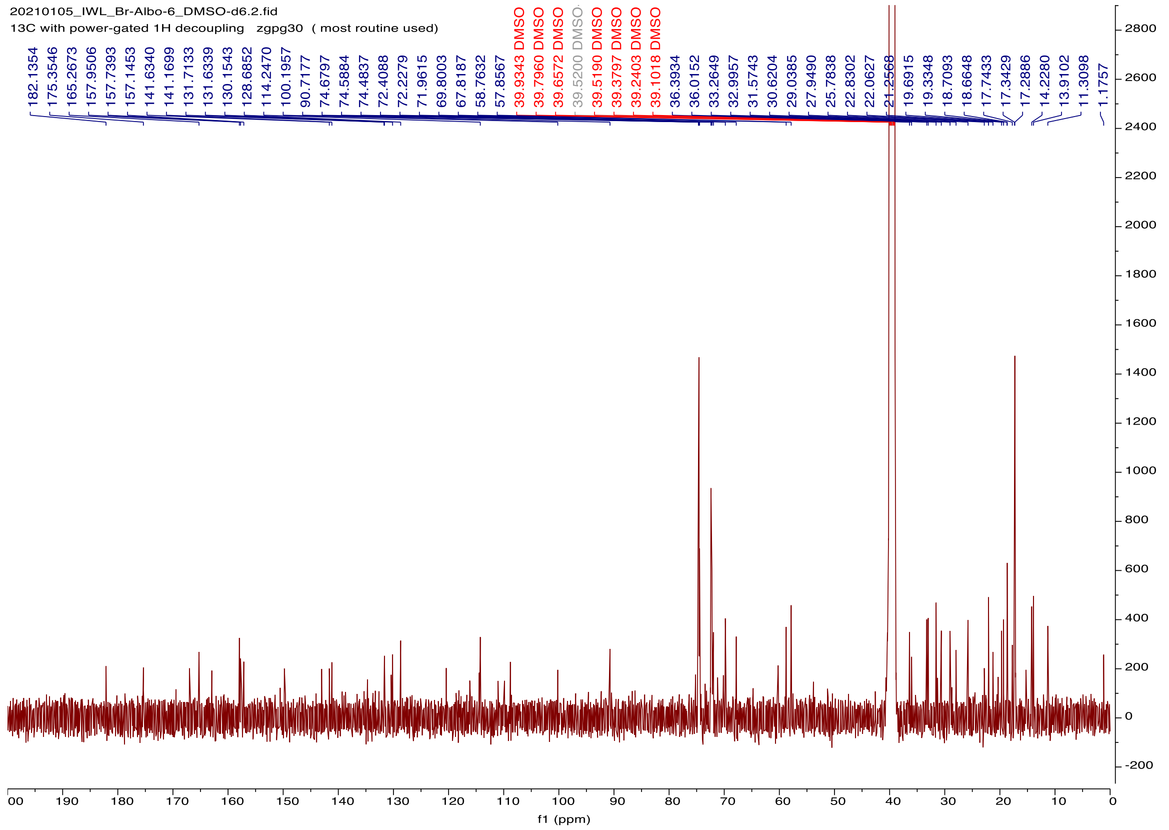

FIG S25. ^{13}C NMR spectrum (150 MHz) of bromoalbofungin (**3**) in DMSO-d_6.

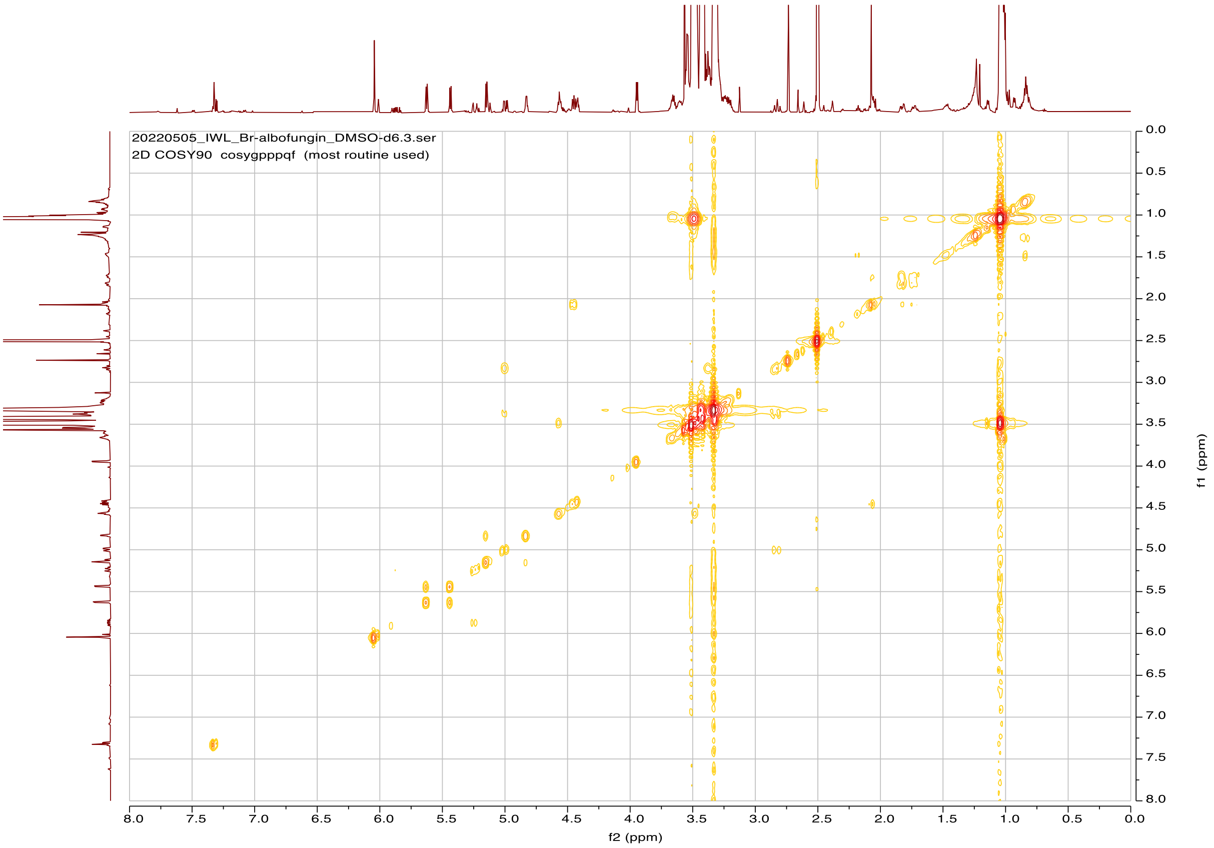

FIG S26. ${}^{1}H$-${}^{1}H$ COSY spectrum of bromoalbofungin (**3**) in DMSO-d_6.

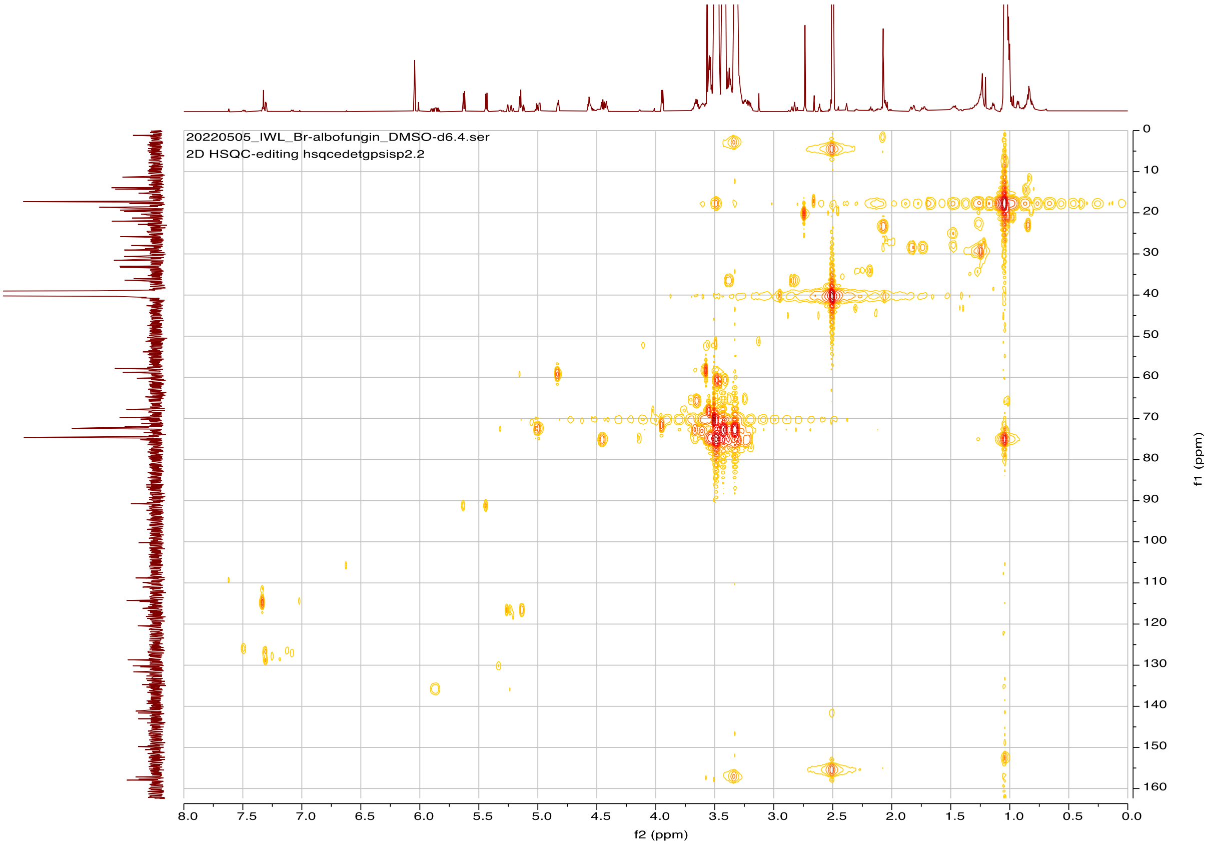

FIG S27. HSQC spectrum of bromoalbofungin (**3**) in DMSO-d_6.

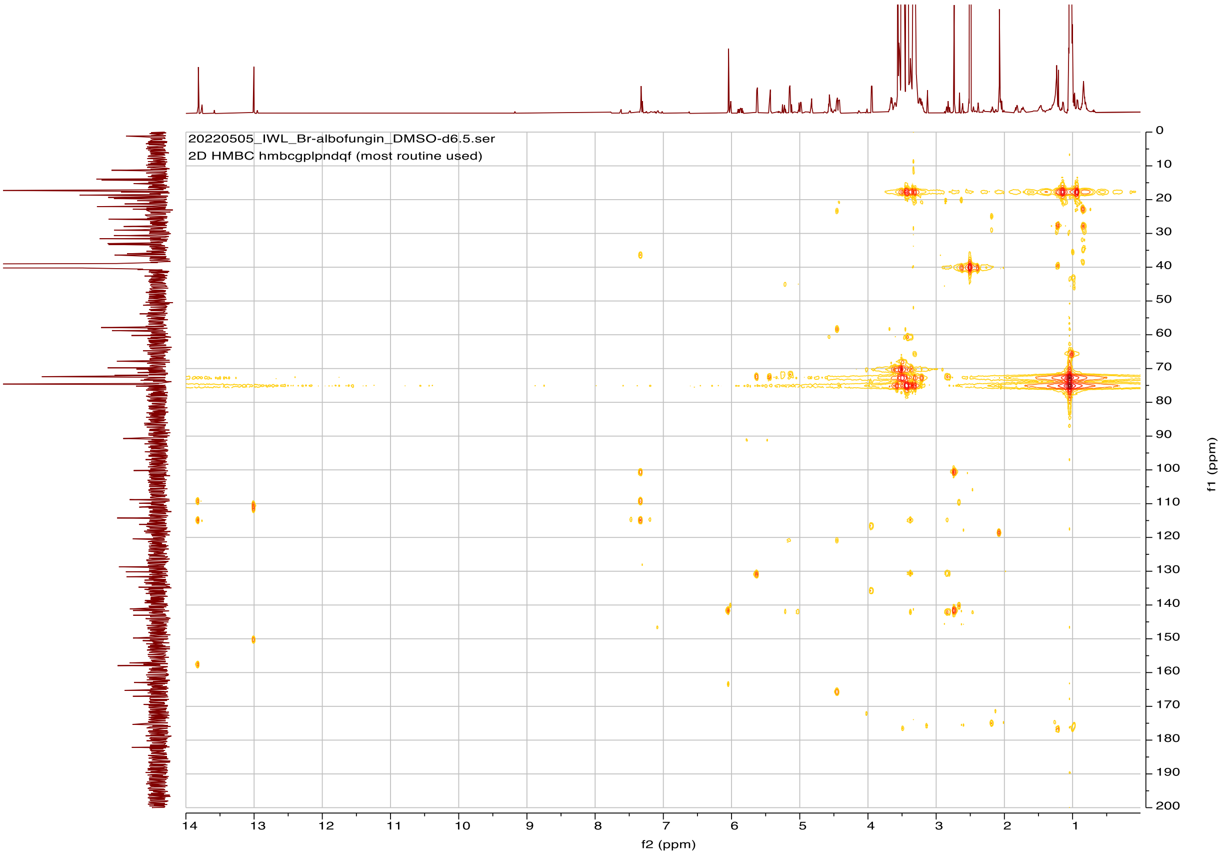

FIG S28. HMBC spectrum of bromoalbofungin (**3**) in DMSO-d_6.

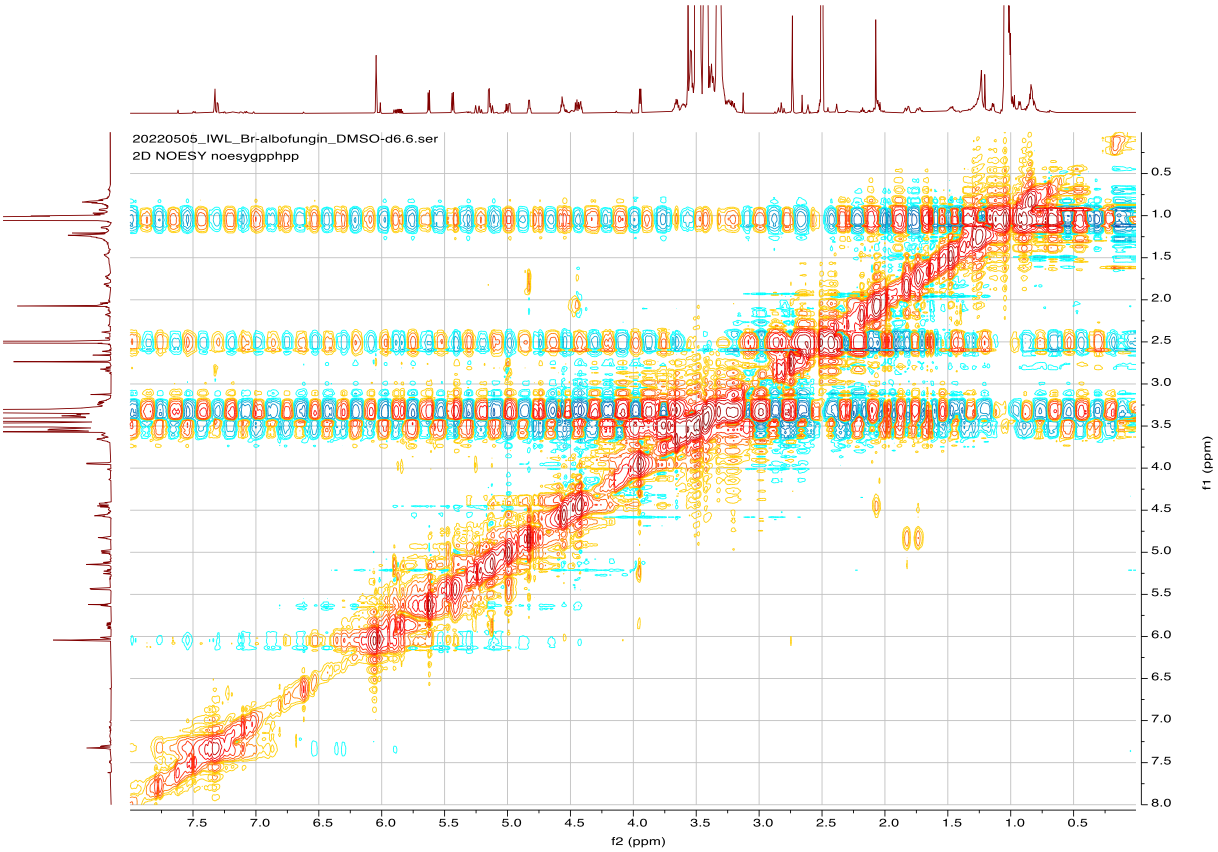

FIG S29. NOESY spectrum of bromoalbofungin (**3**) in DMSO-d_6.

REFERENCES

1. Huang CH, Chen CY, Tsai HH, Chen C, Lin YS, Chen CW. 2003. Linear plasmid SLP2 of Streptomyces lividans is a composite replicon. Mol Microbiol 47:1563-76.
2. Kallifidas D, Jiang G, Ding Y, Luesch H. 2018. Rational engineering of Streptomyces albus J1074 for the overexpression of secondary metabolite gene clusters. Microb Cell Fact 17:25.
3. Wu KM, Li LH, Yan JJ, Tsao N, Liao TL, Tsai HC, Fung CP, Chen HJ, Liu YM, Wang JT, Fang CT, Chang SC, Shu HY, Liu TT, Chen YT, Shiau YR, Lauderdale TL, Su IJ, Kirby R, Tsai SF. 2009. Genome Sequencing and Comparative Analysis of Klebsiella pneumoniae NTUH-K2044, a Strain Causing Liver Abscess and Meningitis. Journal of Bacteriology 191:4492-4501.
4. Horbal L, Fedorenko V, Luzhetskyy A. 2014. Novel and tightly regulated resorcinol and cumate-inducible expression systems for Streptomyces and other actinobacteria. Appl Microbiol Biotechnol 98:8641-55.
5. Sevillano L, Vijgenboom E, van Wezel GP, Diaz M, Santamaria RI. 2016. New approaches to achieve high level enzyme production in Streptomyces lividans. Microb Cell Fact 15:28.
